# Hydroalkylation of Aryl Alkenes with Organohalides Catalyzed by Molybdenum Oxido Based Lewis Pairs

**DOI:** 10.1002/adsc.202000425

**Published:** 2020-06-29

**Authors:** Niklas Zwettler, Antoine Dupé, Sumea Klokić, Angela Milinković, Dado Rodić, Simon Walg, Dmytro Neshchadin, Ferdinand Belaj, Nadia C. Mösch‐Zanetti

**Affiliations:** ^1^ Institute of Chemistry University of Graz Schubertstrasse 1 8010 Graz Austria; ^2^ Institute for Physical and Theoretical Chemistry Graz University of Technology Stremayrgasse 9 8010 Graz Austria

**Keywords:** C−C coupling, Frustrated Lewis pairs, Hydroalkylation, Molybdenum-oxido complexes, Silanes

## Abstract

Three molybdenum(VI) dioxido complexes [MoO_2_(L)_2_] bearing Schiff base ligands were reacted with B(C_6_F_5_)_3_ to afford the corresponding adducts [MoO{OB(C_6_F_5_)_3_}(L)_2_], which were fully characterized. They exhibit Frustrated Lewis‐Pairs reactivity when reacting with silanes. Especially, the [MoO{OB(C_6_F_5_)_3_}(L)_2_] complex with L=2,4‐dimethyl‐6‐((phenylimino)methyl)phenol proved to be active as catalyst for the hydroalkylation of aryl alkenes with organohalides and for the Atom‐Transfer Radical Addition (ATRA) of organohalides to aliphatic alkenes. A series of *gem*‐dichloride and *gem*‐dibromide compounds with potential for further derivatization were synthesized from simple alkenes and organohalides, like chloroform or bromoform, using low catalyst loading.

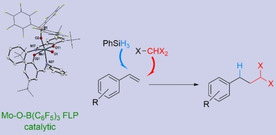

## Introduction

**Scheme 1:**
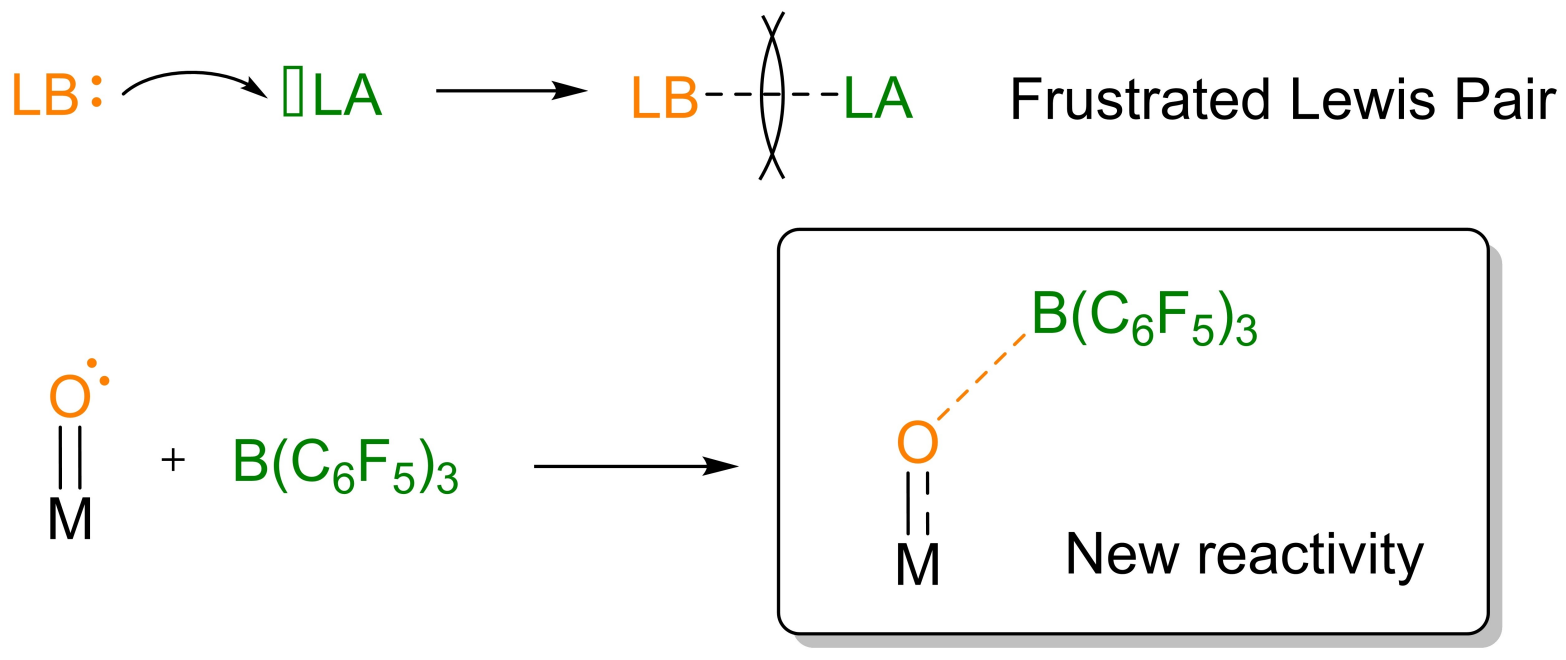
Top: Lewis adduct formation and frustrated Lewis pair; bottom: Lewis adduct formation between M=O and B(C_6_F_5_)_3_.

Since the discovery of their exceptional reactivity by Stephan and Erker,^1^, ^2^ Frustrated Lewis Pairs (FLP) became a tool of choice as catalysts for many chemical transformations, especially in the field of Small Molecules Activation.^2^, ^3^ FLP are Lewis acid‐base pairs that form no or a poor bond between them, for example by deliberately introducing steric hindrance. Among the numerous examples of activation of unreactive molecules by FLP developed over the past ten years, splitting of H_2_ for metal‐free hydrogenation of imines,^4^ carbonyls,^5^ alkenes^6^ or alkynes,^7^ as well as functionalization of CO_2_ 8 are the most prominent examples. In FLP, the role of the Lewis acid is often played by tris(pentafluorophenyl)borane B(C_6_F_5_)_3_, due to its high electrophilicity and steric demand. Besides, B(C_6_F_5_)_3_ is itself a good catalyst for several reactions, especially hydrosilylation reactions.^9^, ^10^ Recently, the concept of FLP was extended to transition metal compounds, where they act as the Lewis acid or base, allowing for further reactivity not observed with main group frustrated Lewis pairs.^11^ In particular, transition metals complexes bearing an oxido ligand could be used as the Lewis base in combination with a Lewis acid such as B(C_6_F_5_)_3_, leading to new reactivity for the complexes, as reported by the group of Schrock^12^ and the group of Ison (Scheme 1).^13^, ^14^

Inspired by these reports, our group became interested in investigating the influence of bulky Lewis acids on oxygen activation and oxidative properties of our previously published molybdenum dioxido and oxido‐imido complexes.^15^, ^16^, ^17^, ^18^ We recently reported the synthesis of a molybdenum oxido‐imido complex that reacted with B(C_6_F_5_)_3_ under formation of an adduct having FLP‐like properties.^19^ With this molybdenum‐oxido based Lewis pair, heterolytic cleavage of silicon‐hydrogen bonds was demonstrated, leading to cationic Mo(VI) species of the formula [Mo(OSiR_3_)(NtBu)L_2_] [HB(C_6_F_5_)_3_] that were isolated and fully characterized. Ultimately, it was shown that such FLP‐like adducts could catalyze the hydrosilylation of benzaldehyde via insertion of the substrate into the boron‐hydrogen bond of the borate.

**Scheme 2:**
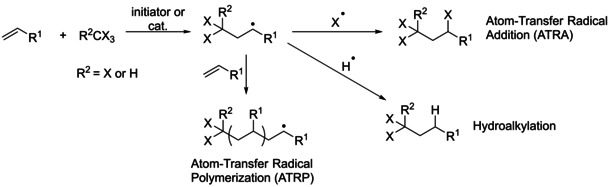
ATRA (Kharasch addition), ATRP and hydroalkylation of alkenes with organohalides.

In this paper, we further investigate the reactivity of these FLP‐like adducts of B(C_6_F_5_)_3_ with molybdenum oxido complexes towards alkenes. We found that an unexpected addition of halides to the alkenes takes place in presence of catalytic amounts of the metal FLP and silanes. The addition of organohalides to alkenes is known as Atom‐Transfer Radical Addition (ATRA) or Kharasch Addition, named after Morris Kharasch, who studied the reaction of HBr, CHCl_3_ and CCl_4_ with unsymmetrical alkenes in presence of peroxides.^20^ The reaction starts with formation of a radical species from the organohalide, generally CX_4_ or CHX_3_, in presence of an initiator or a transition‐metal catalyst (Scheme 2). The generated ^.^CX_3_ or ^.^CHX_2_ radical can then react with the alkene, leading to an anti‐Markovnikov addition intermediate, which can subsequently combine with the other radical ^.^X from the organohalide to afford the final product. Alternatively, the radical intermediate can react with one or several equivalents of the initial alkene to afford a polymer. In this case, the reaction is called Atom‐Transfer Radical Polymerization (ATRP). In a third outcome, the addition intermediate, when sufficiently reactive, can react with a hydrogen donor to lead to the corresponding hydroalkylation product. Such hydroalkylation may also be referred to as a Giese addition.^21^

ATRA reactions are nowadays broadly used for the synthesis of halogenated functionalized molecules.^22^ Ruthenium^23^ or copper^24^ catalysts bearing ligands that could photo‐ or thermo‐initiate the formation of the radical are usually needed to perform ATRA reactions, but examples using molybdenum complexes were also reported.^25^, ^26^ The molybdenum dioxido‐B(C_6_F_5_)_3_ compounds presented in this paper can not only catalyze the ATRA reaction when aliphatic alkenes are used as substrates, but more importantly the hydroalkylation of aryl alkenes since these ones form more reactive intermediates. Hence, simple organohalides like chloroform or bromoform reacted with a variety of alkenes that were reduced forming new *gem*‐dichloride and *gem*‐dibromide compounds that are of interest for further reactivity and functionalization.

## Results and Discussion

**Scheme 3:**
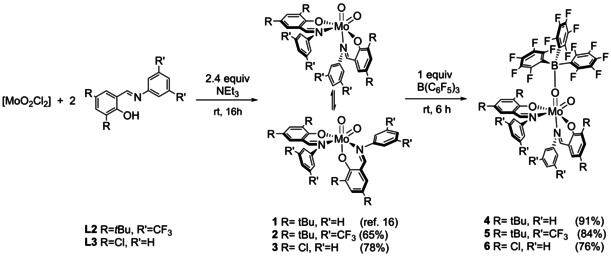
Synthesis of molybdenum dioxido complexes **1**–**3** and molybdenum oxido Lewis adducts **4**–**6**.

**Synthesis of Dioxido Complexes**. Three molybdenum dioxido complexes bearing iminophenolate ligands with different steric and electronic properties were synthesized in order to study their reaction with B(C_6_F_5_)_3_ (Scheme 3). Complex [MoO_2_(**L1**)_2_] (**1**) was synthesized following a previously published procedure^16^ and complexes [MoO_2_(**L2**)_2_] (**2**) and [MoO_2_(**L3**)_2_] (**3**) were synthesized using similar protocols: two equiv of ligands **HL2** 27 or **HL3** 28 were reacted with one equiv of [MoO_2_Cl_2_] in presence of excess NEt_3_ in an appropriate solvent using Schlenk techniques. After filtration and purification, the three molybdenum dioxido complexes were isolated as orange or yellow solids in good yields (65%–78%, Scheme 3). As reported for **1** in our previous publication,^16^ complex **2** exhibits a dynamic isomerism in solution, reflected by two distinct sets of resonances with different intensity in the ^1^H and ^19^F NMR spectra. Complex **3** is very poorly soluble in organic solvents and reliable NMR spectroscopy data could be only obtained using DMSO. For this complex, only one set of signals is clearly visible, while formation of a second isomer might be the cause of the other broad signals (see SI). As confirmed by X‐ray crystallography, one of the two isomers for each complex has nitrogen atoms of both ligands *trans* to the oxido group (N,N isomer), whereas the N,O isomer forms as well but did not crystallize in our attempts. The three complexes are slightly sensitive towards moisture and only partially soluble in acetonitrile.

**Synthesis of Molybdenum Oxido Lewis Adducts**. In a similar procedure to a previous publication,^19^ addition of one equiv of the Lewis acid B(C_6_F_5_)_3_ to the yellow suspension of the molybdenum(VI) dioxido precursors **1**–**3** in pentane led to an immediate color change to deep red and subsequent formation of the Lewis adducts [MoO{OB(C_6_F_5_)_3_}(**L**)_2_] (**L1**–**L3**, **4**–**6**) as deep red crystalline precipitates. Compounds **4**–**6** were isolated as red to dark red solids in very good yields after purification (Scheme 3).

Compounds **4**–**6** are highly sensitive to moisture and soluble in most polar organic solvents, but only sparingly soluble in benzene and toluene. Like previous observations for the related oxido imido borane adduct,^19^ NMR spectroscopy reveals that compounds **4**–**6** exist as single isomers in solution, which is in contrast to the isomeric equilibrium observed for the dioxido molybdenum complexes **1**–**3**. The ^1^H NMR spectra feature two distinct signal sets for the ligands, indicating coordination at only one Mo=O moiety. The coordination of the Lewis acid is confirmed by a new set of signals corresponding to the *meta*, *ortho* and *para* fluorines of B(C_6_F_5_)_3_, observable in ^19^F NMR spectroscopy. Especially, the pronounced shift of the *para*‐F resonance (−158.8 ppm, C_6_D_6_) compared to free borane (−142.3 ppm, C_6_D_6_) is characteristic of such coordination.^14^, ^19^ The *para*‐F shift is less pronounced in both adducts **5** (−148.2 ppm, C_6_D_6_) and **6** (−157.6 ppm, C_6_D_6_). Due to the quadrupolar nature of ^11^B nucleus, ^11^B NMR spectroscopy gave no meaningful data for the complexes **4**–**6**, but IR spectroscopy was used to confirm Mo=O−B coordination, with a characteristic strong signal at around 980 cm^−1^ for each complex.^19^

**Figure 1:**
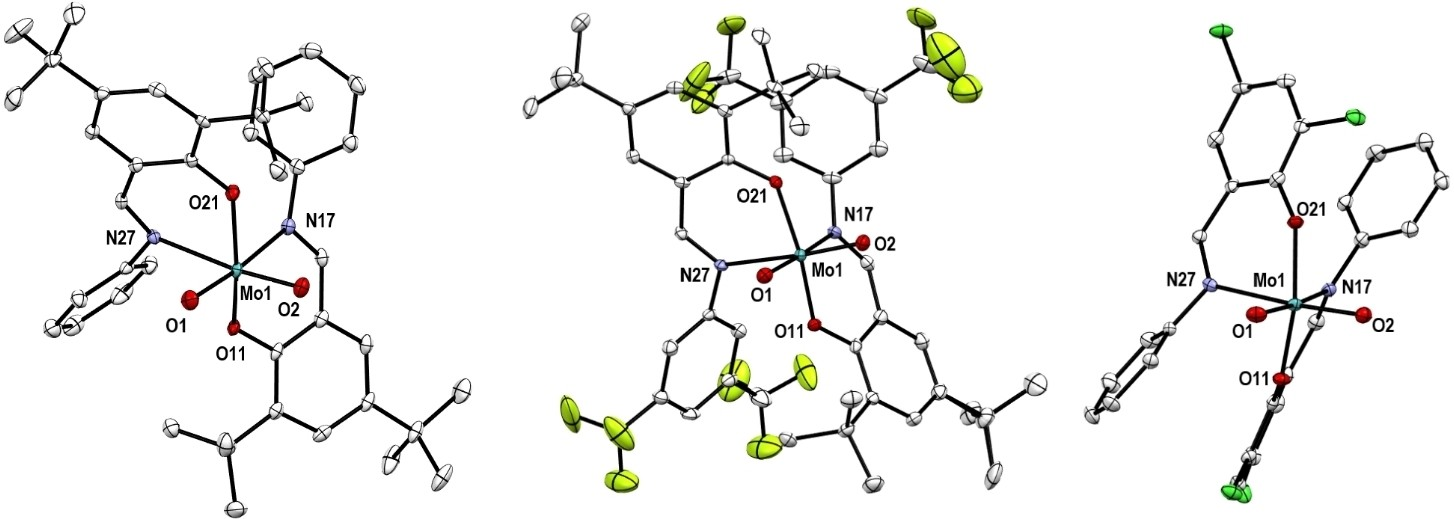
Molecular views (50% probability level) of **1**–**3** (from left to right); hydrogen atoms as well as solvent molecules are omitted for clarity. For disordered fragments, only atoms with the higher site occupation factors are depicted.

**Figure 2:**
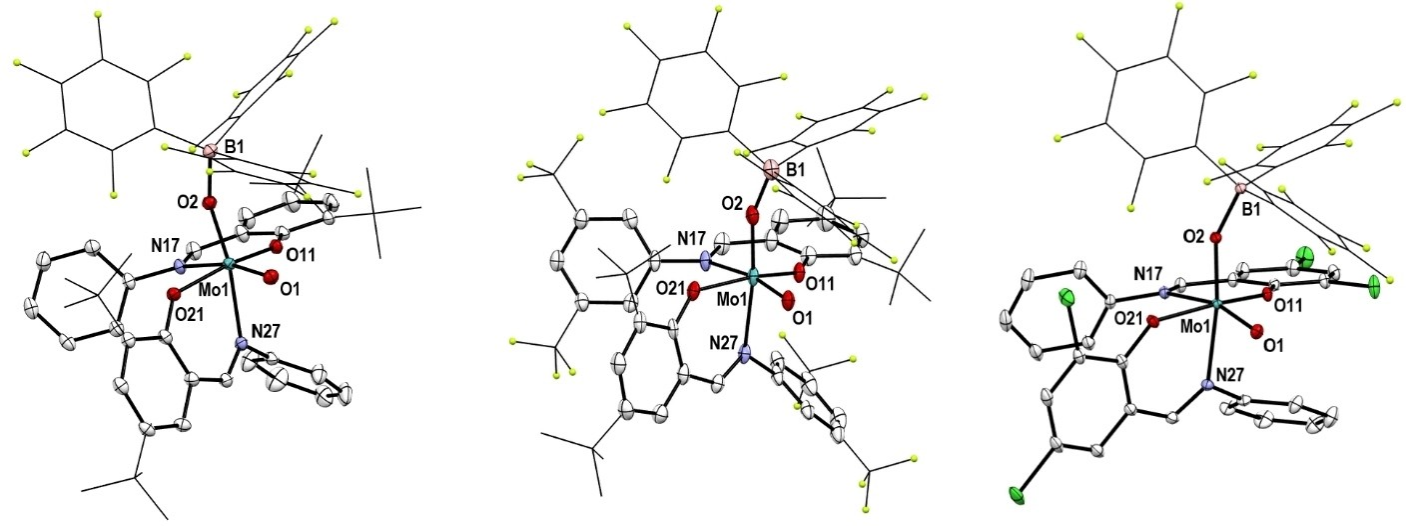
Molecular views (50% probability level) of **4**–**6** (from top to bottom); hydrogen atoms as well as solvent molecules are omitted for clarity. For disordered fragments, only atoms with the higher site occupation factors are depicted.

**Molecular Structures**. The molecular structures of complexes **1**–**3** and **4**–**6** were determined by single crystal X‐ray diffraction analyses. The molecular views of complexes **1**–**3** are shown in Figure 1 and of **4**–**6** in Figure 2. Selected bond lengths and angles are shown in Table 1 and full crystallographic details are provided in the supporting information. The dioxido complexes **1**–**3** show similar structures, as already reported for **1** 16 and for other published molybdenum dioxido complexes.^15^, ^18^ As reported for similar oxido‐imido complexes forming Lewis adducts,^19^ the Mo=O bond that interacts with B(C_6_F_5_)_3_ in **4**–**6** becomes elongated in comparison to the parent dioxido complexes **1**–**3**. Thus, the Mo−N bond trans to the oxido‐borane moiety is shortened for all three compounds when compared with Mo−N bond from dioxido complexes. Overall, the structures of compounds **4**–**6** are similar in terms of bond lengths, with the Mo1−O2 bond ranging from 1.783(2) Å in **5** to 1.7900(10) Å in **4** and the O2−B1 bond ranging from 1.530(2) Å in **4** to 1.5371(15) Å in **6**. The major difference between complex **4** and **5**–**6** is the angle B1−O2−Mo1 being larger in the case of **4** [159.08(9)°] compared to 155.8(2)° for **5** and 153.13(8)° for **6**, due to the presence of *tert*‐butyl and phenyl groups at the ligand.

**Table 1:** Selected bond lengths [Å] and angles [°] for complexes **1**–**3** and **4**–**6**.

	**1**	**2**	**3**
Mo1−O1	1.6983(15)	1.6989(17)	1.705(4)
Mo1−O2	1.6984(13)	1.7048(17)	1.712(3)
Mo1−O11	1.9608(13)	1.9404(16)	1.950(3)
Mo1−O21	1.9409(13)	1.9412(16)	1.946(4)
Mo1−N17	2.3896(16)	2.395(2)	2.407(4)
Mo1−N27	2.3898(15)	2.358(2)	2.382(4)
O1−Mo1−O2	105.22(7)	105.97(9)	104.04(18)
O11−Mo1−O21	152.82(5)	154.66(7)	154.52(14)
O1−Mo1−N17	169.72(6)	167.87(8)	167.46(16)
O2−Mo1−N27	170.77(6)	167.74(8)	165.53(15)
N17−Mo1−N27	86.27(5)	81.84(7)	77.19(14)

	**4**	**5**	**6**
Mo1−O1	1.6909(11)	1.680(2)	1.6892(9)
Mo1−O2	1.7900(10)	1.783(2)	1.7839(8)
Mo1−O11	1.9008(10)	1.8937(19)	1.9121(9)
Mo1−O21	1.9229(10)	1.922(2)	1.9072(9)
Mo1−N17	2.3569(12)	2.381(3)	2.3950(10)
Mo1−N27	2.2967(13)	2.312(3)	2.3084(10)
O2−B1	1.530(2)	1.535(4)	1.5371(15)
O1−Mo1−O2	104.47(5)	103.85(10)	104.37(4)
O11−Mo1−O21	160.52(5)	157.21(9)	157.99(4)
O1−Mo1−N17	163.63(5)	165.94(10)	159.94(4)
O2−Mo1−N27	169.59(4)	171.60(10)	169.94(4)
N17−Mo1−N27	79.02(4)	82.21(9)	74.80(3)
B1−O2−Mo1	159.08(9)	155.8(2)	153.13(8)

**Scheme 4:**
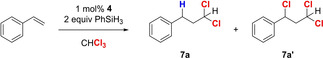
Observed catalytic transformation of styrene into 3,3‐dichloropropyl benzene (**7 a**) and 1,3,3‐trichloropropylbenzene (**7 a′**).

**Reactivity in ATRA and Hydroalkylation of alkenes**. During initial attempts to use complex **4** as catalyst in olefin hydrosilylation with phenylsilane, we noticed unexpected reactivity upon use of CHCl_3_ as reaction solvent. Thus, using styrene as substrate, we did not observe the hydrosilylated product, but it was rather converted to 3,3‐dichloropropyl benzene (**7 a**) and to 1,3,3‐trichloropropylbenzene (**7 a’**) (Scheme 4). This unexpected observation prompted us to investigate the generalizability and scope of this reaction in terms of catalyst, olefinic substrate, organohalide and silane. In this reaction, 1,3,3‐trichloropropylbenzene (**7 a’**) is formed by addition of chloroform to styrene, representing an ATRA reaction, which does typically require no silane nor borane. Such catalytic transformation was already reported to be catalyzed by molybdenum(VI) complexes in presence of triethylamine using CCl_4_ as organohalide.^26^ We envisioned that formation of 3,3‐dichloropropyl benzene (**7 a**) most likely occurs due to the presence of silane (vide infra).

With this knowledge at hand, we performed a series of experiments in order to evaluate which of the components of the reaction shown in Scheme 4 are needed for the catalytic conversion. The results are summarized in Table 2. Styrene was fully converted to **7 a** in 91% yield and **7 a’** in 9% yield using 1 mol% catalyst **4** with 2 equiv PhSiH_3_ after 2 h at 50 °C (entry 1). Using 1 equiv PhSiH_3_ also afforded **7 a** with almost complete conversion of the substrate, albeit slower (entry 2). Blank and control experiments revealed no conversion of styrene in absence of complex **4** (entry 3), in absence of silane or in absence of borane (using the dioxido complex **1**, entry 4). The latter was corroborated using commercially available [MoO_2_(acac)_2_], with which also no reactivity was observed (entry 5). Furthermore, while the use of B(C_6_F_5_)_3_ did lead to a partial consumption of styrene, no formation of the chlorinated products was observed. Instead, using B(C_6_F_5_)_3_ led to a mixture of unidentified products accompanied by small quantities of the hydrosilylation product (entry 6). It is also interesting to note that the use of the two other borane adducts **5** or **6**, or the presence of [MoO_2_(acac)_2_] and B(C_6_F_5_)_3_ together in the reaction mixture, although showing slow conversion of styrene, lead to no formation or in a small amount of chlorinated products (entry 7 to 9).

**Table 2:** Control experiments for the addition of CHCl_3_ to styrene in presence of silane.

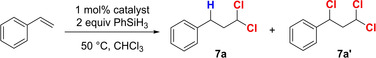						
Entry	Catalyst (1 mol%)	PhSiH_3_ (equiv)	Reaction time	Conversion (%)	Selectivity for **7 a** (%)	Selectivity for **7 a’** (%)
1	**4**	2	2 h	>98	91	9
2	**4**	1	2 h/5 h	95/>98	77/80	23/16
3	none	2	20 h	0	0	0
4	**1**	2	20 h	0	0	0
5	[MoO_2_(acac)_2_]	2	20 h	0	0	0
6	B(C_6_F_5_)_3_	2	20 h	5	0	0
7	**5**	2	2 h/20 h	56/95	0/7	0/2
8	**6**	2	2 h/20 h	11/61	0/0	0/0
9	[MoO_2_(acac)_2_]+B(C_6_F_5_)_3_	2	20 h	83	0	0

General conditions: 1 or 2 equiv PhSiH_3_, 50 °C, 0.5 mL CHCl_3_ as solvent. Conversion of styrene as determined by GC‐MS and selectivity as determined by ^1^H NMR spectroscopy.

Thus, exclusively the combination of the oxido‐adduct **4** with silane as shown in Scheme 4 leads to catalytic hydroalkylation of styrene.

With catalyst **4**, reaction optimization in toluene with varying amounts of CHCl_3_ was performed revealing optimal conversion and selectivity for **7 a** with 5 equiv of chloroform (Table 3, entries 2–4). Solvent screening with 5 equiv of CHCl_3_ showed 93% conversion of styrene in chlorobenzene were after 2 h (entry 5), while no consumption of styrene took place in acetonitrile as it coordinates to B(C_6_F_5_)_3_ (entry 6).

**Table 3:** Solvent screening for the addition of CHCl_3_ to styrene in presence of silane.

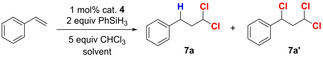					
Entry	Solvent	Equiv CHCl_3_	Conv. (%)	Select. for **7 a** (%)	Select.for **7 a’** (%)
1	CHCl_3_	–	>98	91	9
2	Tol.	1	0	0	0
3	Tol.	2	60	73	0
4	Tol.	5	77	75	7
5	PhCl	5	93	92	8
6	MeCN	5	0	0	0

General conditions: 1 mol% cat **4**, 2 equiv PhSiH_3_, 50 °C, 2 h. Conversion of styrene as determined by GC‐MS and selectivity as determined by ^1^H NMR spectroscopy.

As the selectivity toward formation of 3,3‐dichloropropyl benzene is very high in chlorobenzene, where only the ATRA product was observed as a side product, the latter was used as preferred solvent for the reaction.

In order to evaluate the scope of applicable chlorinated substrates, styrene was reacted with various organochlorides and organobromides. Results are summarized in Table 4. Chloroform and carbon tetrachloride react straightforward with styrene using 1 mol% of **4**, affording the corresponding hydroalkylation products with good selectivity (Table 4, entry 1 and 2).

**Table 4:** Hydroalkylation of styrene with different organohalides catalyzed by **4** in presence of phenylsilane.

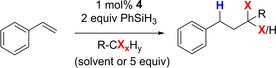						
Entry	R−CX_x_H_y_	solvent	Time (h)	Cat. loading (mol%)	Conversion (%)	Selectivity for hydroalkylation product (%)
1	CHCl_3_	–	2	1	>98	91
2	CCl_4_	PhCl	20	1	100	70
3	CH_2_Cl_2_	–	20	1	0	0
4	CHBr_3_	PhCl	1	1	100	>98
5	CBr_4_	PhCl	3	1	100	25
6	CHI_3_	PhCl	20	1	0	0
7	MeCCl_3_	PhCl	20	2	0	0
8	PhCCl_3_	PhCl	20	2	0	0
9	NCCCl_3_	toluene	20	1	0	0
10	(EtO_2_C)_2_CHBr	PhCl	20	2	0	0

General conditions: 2 equiv PhSiH_3_, 50 °C, 0.5 mL solvent. Conversion of styrene as determined by GC‐MS and selectivity as determined by ^1^H NMR spectroscopy.

It is noteworthy that the main side product in these reactions is the ATRA product, where the vicinal carbon of the aryl group is substituted by a chlorine atom. Bromoform is the most reactive organohalide, leading to 100% conversion and almost selective formation of the hydroalkylation product after only 1 hour (Table 4, entry 4). Despite full conversion of styrene after 3 h, the reaction with carbon tetrabromide resulted in only a small amount of brominated product, possibly due to the instability of CBr_4_ (Table 4, entry 5). Bulkier CHI_3_ or substituted organohalides such as MeCCl_3_, PhCCl_3_, NCCCl_3_ or diethyl bromomalonate did not react under the reaction conditions (entry 6 to 10). Dichloromethane proved unreactive as well (entry 3).

**Figure 3:**
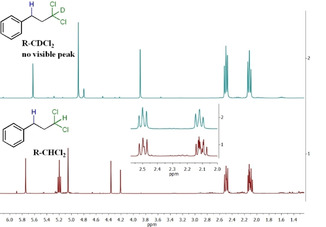
^1^H NMR spectra of hydroalkylation of styrene using CDCl_3_ (top) or CHCl_3_ in C_6_D_6_ (bottom) as the organohalide.

The fact that the reaction with CCl_4_ lead mainly to formation of the hydroalkylation product is a proof that the silane is the source of the hydrogen atom added to the double bond. In order to confirm this, an experiment using CDCl_3_ as the organohalide in the hydroalkylation of styrene was performed, then the ^1^H NMR spectrum in CDCl_3_ of the obtained product was compared to the one when CHCl_3_ is used in C_6_D_6_ (Figure 3).

Disappearance of the triplet signal at 5.2 ppm could be observed and the integration for the two signals corresponding to the two CH_2_ groups remained the same, indicating that the CDCl_2_ moiety is incorporated to the product and no C−H bond from the organohalide is cleaved during the reaction. This ^1^H NMR spectroscopy study of the reaction of styrene with CHCl_3_ in C_6_D_6_ in presence of PhSiH_3_ and **4** confirmed the formation of PhSiH_2_Cl and PhSiHCl_2_ as side‐products from the reaction. Furthermore, the hydroalkylation of styrene takes place and affords **7 a** not only with phenylsilane but also with secondary and tertiary silanes, albeit slower (Table 5).

**Table 5:** Silane screening for the addition of CHCl_3_ to styrene in toluene.

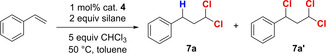					
	Silane	Time	Conv. (%)	Select. for **7 a** (%)	Select.for **7 a′** (%)
1	PhSiH_3_	2 h	77	96/>98	4/0
2	PhMeSiH_2_	2 h/20 h	72/>98	76/>98	0/0
3	Et_3_SiH	2 h/20 h	0/51	0/88	0/0
4	PhMe_2_SiH	20 h	44	0	0

General conditions: 1 mol% cat **4**, 2 equiv Silane, 5 equiv CHCl_3_, 50 °C in toluene. Conversion of styrene as determined by GC‐MS and selectivity as determined by ^1^H NMR spectroscopy.

**Figure 4:**
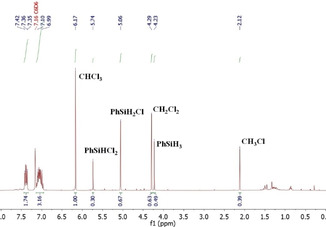
^1^H NMR spectrum in C_6_D_6_ of the chlorination of PhSiH_3_ with CHCl_3_ in presence of **4** without radical scavenger.

The observation of two different products in the here described system is in contrast to a classical ATRA, where the halide of the organohalide is incorporated to the alkene substrate. Since the ATRA and the hydroalkylation reactions involve formation of radical species, we investigated if indeed our catalytic reaction proceeds in a similar fashion. Addition of the free radical‐scavenger galvinoxyl to the typical reaction mixture for the hydroalkylation of styrene (1 mol% **4**, 2 equiv PhSiH_3_, 5 equiv CHBr_3_) shut down the reactivity and styrene remained intact, providing evidence that the organohalide is added to the alkene via formation of radical species. Furthermore, a ^1^H NMR spectroscopy study of the reaction of PhSiH_3_ with CHCl_3_ in C_6_D_6_ in presence of **4**, without substrate, confirmed the formation of radical species (Figure 4). In these conditions, PhSiH_3_ reacts with CHCl_3_ to form a mixture of PhSiH_2_Cl, PhSiHCl_2_, CH_2_Cl_2_ and CH_3_Cl, similar to the chlorination of hydrosilanes reported by Chulsky and Dobrovetsky.^10^

When the reaction is run with a stoichiometric amount of galvinoxyl, the ^1^H NMR spectrum showed no exchange between PhSiH_3_ and CHCl_3_. This indicates that PhSiH_3_ reacts with the organohalide in presence of borane adduct **4** generating a radical species. This exchange reaction with PhSiH_3_ is very fast for CHBr_3_, but surprisingly does not proceed when using CHI_3_.

**Scheme 5:**
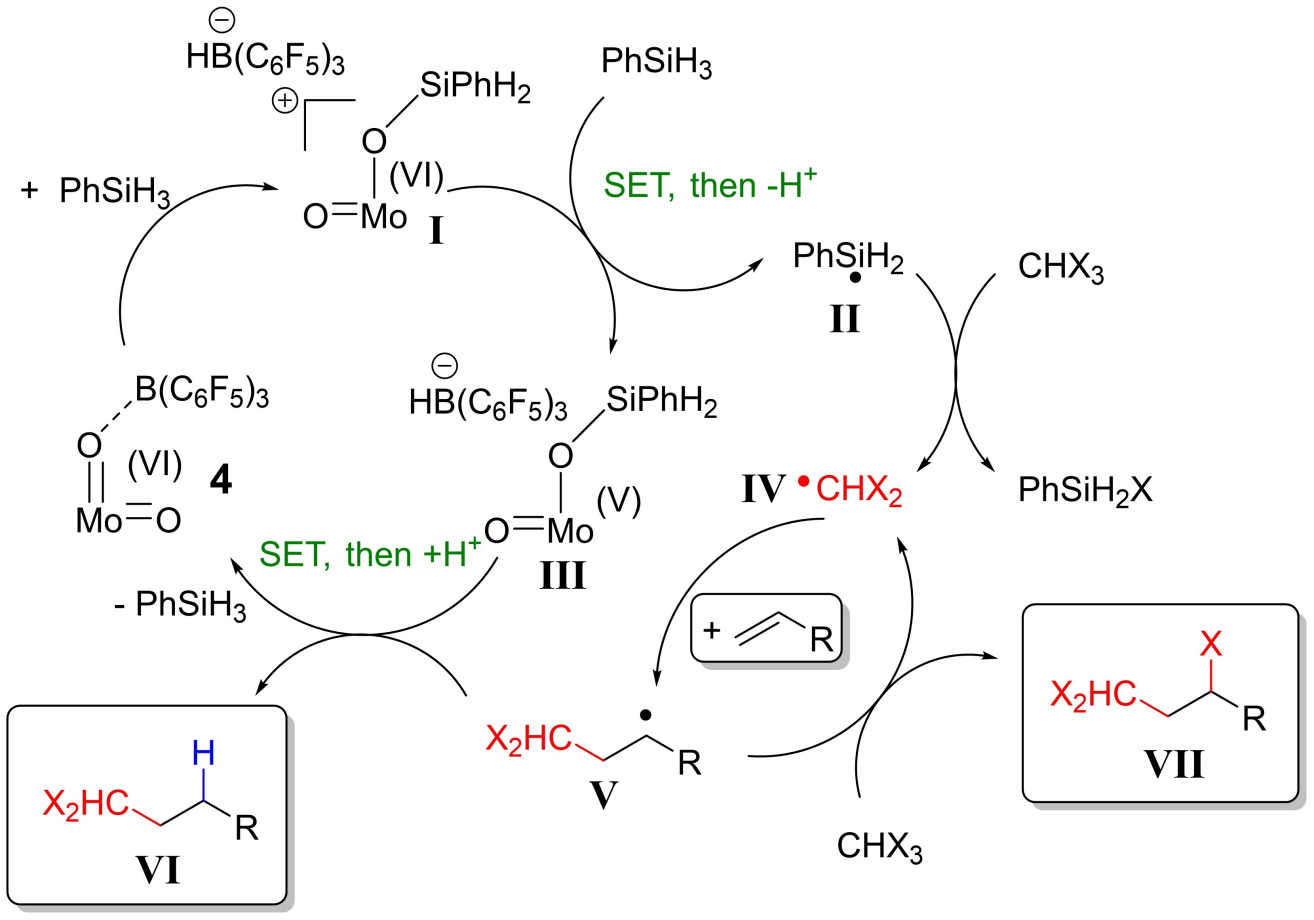
Plausible mechanism for the hydroalkylation or ATRA of an alkene with an organohalide in presence of **4** and PhSiH_3_.

Besides, an EPR measurement of the reaction of complex **4** with 1 equiv PhSiH_3_ in C_6_D_6_ without substrate or organohalide was performed and showed that the reaction led to formation of a Mo(V) intermediate (see Supporting Information). Although this intermediate might not be the catalytically active species, this result shows that reduction of the molybdenum center occurs during the reaction. The reactivity of complexes **5** and **6** was also tested without substrate to see if this chlorination of PhSiH_3_ takes place. We observed the same formation of PhSiH_2_Cl and CH_2_Cl_2_, but only in trace amounts and the reaction is slower than for **4**. This lack of reactivity of PhSiH_3_ with CHCl_3_, after reacting with the FLP adducts **5** or **6**, could be explained by the presence of electron‐withdrawing substituents at the ligands. The Mo(V) intermediate arising from the reaction of PhSiH_3_ with **5** or **6** would be stable enough to react with the radical species or other molecules present in the mixture, leading to deactivation of the catalyst, while for complex **4**, the Mo(V) intermediate is probably a transient species, allowing reaction of H_2_PhSi^.^ with CHCl_3_. These results led us to consider a plausible mechanism for the reaction similar to the mechanism reported by Hell et al. for the silyl radical‐mediated activation of sulfamoyl chlorides (Scheme 5).21d

In the proposed mechanism, PhSiH_3_ reacts at first with the FLP complex **4** to form the corresponding Lewis Pair **I**, that undergoes Single Electron‐Transfer (SET) to activate another PhSiH_3_ molecule, leading to the radical H_2_PhSi^.^ **II**, a Mo(V) intermediate **III** and a free proton. H_2_PhSi^.^ then abstracts a halogen atom of the organohalide, forming the side‐product PhSiH_2_X and the radical ^.^CHX_2_ **IV**. This radical adds to the alkene, forming a new reactive radical intermediate **V**. The catalytic cycle is closed by recombination of this radical with HB(C_6_F_5_), affording the final product **VI**, and a SET regenerating the Mo(VI) complex **4**. In the case of aliphatic alkenes, the radical intermediate **V** is less reactive and has time to react with the organohalide, leading to ATRA product **VII**.

**Scheme 6:**
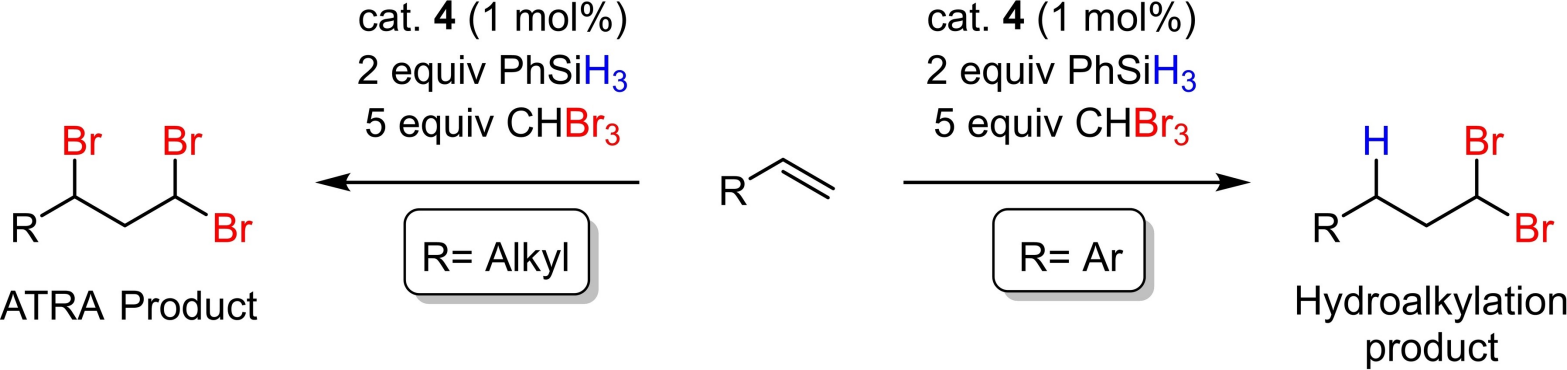
Different outcome of the reaction of various alkenes with CHBr_3_ in presence of catalyst **4** and phenylsilane.

As reactions with bromoform lead exclusively to the hydroalkylation product, the experiments to extent the scope of the olefinic substrates as summarized in Table 6 were performed using CHBr_3_ as the organohalide. The most striking observation concerns the substrate structure, where a phenyl group adjacent to the double bond led to quick conversion into the hydroalkylation product (3,3‐dibromo derivatives). Aliphatic substrates with terminal double bonds reacted much slower and formed the ATRA product (1,3,3‐tribromo derivatives) exclusively (Scheme 6). The reaction of cyclooctene with CHBr_3_, although showing conversion of the substrate, did not lead to formation of brominated product. However, reaction of cyclooctene with CCl_4_ in chlorobenzene using 1 mol% of catalyst **4** shows full conversion of the substrate to a mixture of 1,2‐ and 1,4‐addition products, as reported in previous publications (Table 6, entry 3).^29^

**Table 6:** Products scope of the reaction of various alkenes with organohalides in presence of catalyst **4** and phenylsilane.

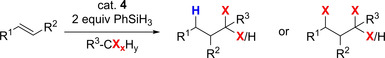							
Entry	Substrate/Conversion (%)		Organohalide	Product/Isolated yield (%)		Catalyst **4** loading (mol%)	Time (h)
1	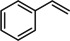	>98	CHCl_3_	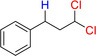	**7 a**, 86 (GC)	1	2
2	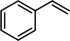	>98	CHBr_3_	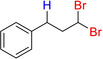	**7 b**, 86	1	3
3	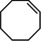	100	CCl_4_	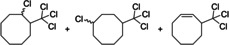		1	2
4	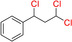	79	CHCl_3_	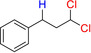	**7 a**, 79	1	20
5	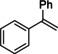	100	CHBr_3_	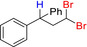	**7 c**, 90	1	1
6	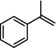	100	CHBr_3_	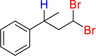	**7 d**, 96	2	1
7	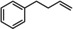	57	CHBr_3_	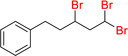	**7 e**, 42	2	24
8	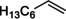	46	CHBr_3_	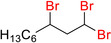	**7 f**, 41	2	24
9	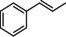	95	CHBr_3_	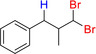	**7 g**, 76	3	24
10	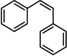	36	CHBr_3_	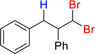	**7 h**, 18	2	24
11	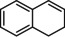	71	CHBr_3_	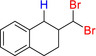	**7 i**, 46	1	24
12	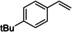	100	CHBr_3_	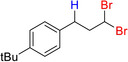	**7 j**, 90	1	3
13	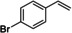	100	CHBr_3_	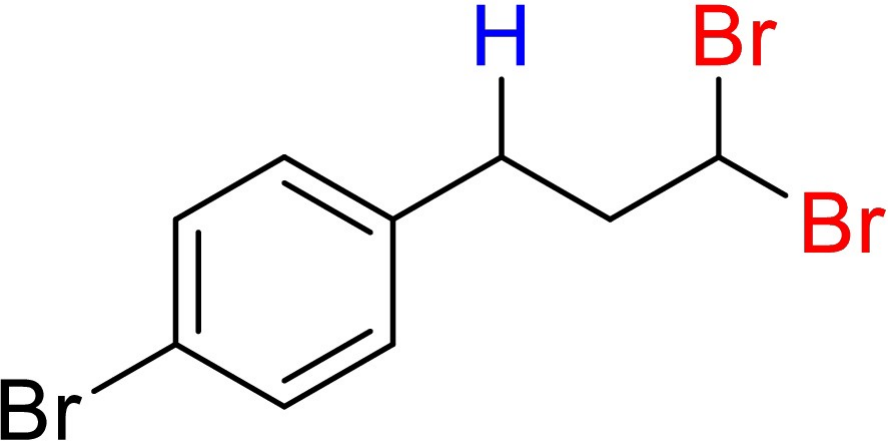	**7 k**, 90	1	3
14	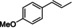	100	CHBr_3_	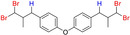	**7 l**, 18	2	5
15	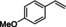	100	CHBr_3_	unidentified		1	24
16	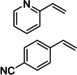	0	CHBr_3_	–		1	24

General conditions: 1 mmol substrate, 2 equiv PhSiH_3_, 5 equiv organohalide, 50 °C in chlorobenzene. Conversion of substrate as determined by GC‐MS.

Formation of the ATRA product as side product was also observed for styrene when using CCl_4_ or CHCl_3_, but it is interesting to see that these ATRA products can react in the reaction conditions to form the hydroalkylation product (entry 4). Furthermore, alkenes with a phenyl substituent proved to be significantly more reactive if terminal rather than internal. Hence, aryl‐substituted alkenes such as styrene, 1,1‐diphenylethene or α‐methylstyrene react quickly with CHBr_3_ with low catalyst loading to afford the corresponding hydroalkylation products **7 a**–**d** in excellent isolated yields (entry 2, 5 and 6). On the other hand, reaction of 4‐phenyl‐1‐butene or 1‐octene with bromoform using 2 mol% of complex **4** in presence of phenylsilane lead to the isolation of Kharasch addition products (3,5,5‐tribromopentyl)benzene **7 e** and 1,3,3‐tribromononane **7 f** in 42% and 41% yield, respectively (entry 7 and 8). Substrates that possess an aryl group adjacent to an internal double bond, such as prop‐1‐en‐1‐ylbenzene react with CHBr_3_ to afford the hydroalkylation products **7 g**–**i**, albeit requiring longer reaction time and higher catalyst loading (entry 9–11). We also investigated the influence of substituents at the aryl group onto the activity of the catalyst. Using 1‐bromo‐4‐vinylbenzene or 1‐(*tert*‐butyl)‐4‐vinylbenzene as substrate lead to excellent isolated yields, 90% for both products **7 j** and **7 k**, after 3 h using 1 mol% catalyst (entry 12–13). Having a methoxy group trans to the vinyl functionality lead to formation of numerous side products by reaction with the silane. No halogenation product was observed in the case of 1‐methoxy‐4‐vinylbenzene, and the hydroalkylation product **7 l** of the reaction with *trans*‐anethole appears to involve etherification of the anethole (entry 14 and 15). However, reaction with 4‐vinylbenzonitrile or 2‐vinylpyridine did not lead to conversion of the substrate, possibly because of the interaction of the donor atom with the borane moiety (entry 16).

## Conclusion

In summary, we synthesized and characterized a molybdenum‐oxido Frustrated Lewis Pair adduct [MoO{OB(C_6_F_5_)_3_}(L)_2_] (L=2,4‐dimethyl‐6‐((phenylimino)methyl)phenol) which is able to react with phenylsilane to catalyze the hydroalkylation of various aryl alkenes with organohalides and the ATRA of organohalides to aliphatic alkenes. Examples of such catalytic hydroalkylation of alkene using simple chlorine and bromine derivatives, like bromoform, are scarce. This catalytic system leads to formation of *gem‐*dichloride and *gem‐*dibromide derivatives in very good yields using low catalyst loadings.

## Experimental Section

**General Informations**. If not otherwise noted, reactions were carried out under N_2_ atmosphere, using standard Schlenk‐techniques or a N_2_‐filled glovebox. The substrates were purchased from commercial sources and used as received. Solvents were purified via a Pure‐Solv MD‐4‐EN solvent purification system from Innovative Technology, Inc. CHCl_3_, CCl_4_ and chlorobenzene were purchased from commercial sources and distilled prior to use. The complex [MoO_2_(**L1**)_2_],^16^ the Schiff base ligands **HL2** 27 and **HL3** 28 as well as B(C_6_F_5_)_3_ 30 were synthesized according to previously published literature. The ^1^H, ^11^B, ^13^C and ^19^F NMR spectra were recorded on a Bruker Optics instrument at 300/96/75/282 MHz. Peaks are denoted as singlet (s) doublet (d), doublet of doublets (dd), triplet (t), quartet (q) and multiplet (m), broad peaks are denoted (br) and all peaks are referenced to the solvent residual signal. Shifts in ^11^B and ^19^F NMR spectra are referenced to external standards (BF_3_ ⋅ Et_2_O and CFCl_3_, respectively). Used solvents and peak assignment are mentioned at the specific data sets. GC‐MS analyses were performed with an Agilent 7890A GC system with an Agilent 19091J‐433 column coupled to a 5975C inert XL EI/CI mass selective detector (MSD). IR spectra were measured as solid samples on a Bruker Alpha‐P Diamond FTIR‐ATR spectrometer. Elemental analyses were carried out using a Heraeus Vario Elementar automatic analyzer at the Institute of Inorganic Chemistry at the Graz University of Technology.

**Synthesis of Complex 2 [MoO_2_(L2)_2_]**. [MoO_2_Cl_2_] (1 equiv, 0.12 g, 0.60 mmol) was added to a solution of **HL2** (2.1 equiv, 0.56 g, 1.26 mmol) and NEt_3_ (2.4 equiv, 0.2 mL, 1.43 mmol) in acetonitrile (5 mL) under stirring. The addition was accompanied by a color change from bright yellow to orange‐red. The reaction mixture was subsequently stirred overnight at room temperature, whereupon a yellow precipitate had formed. The precipitate was filtered off, washed with cold acetonitrile (3×2 mL) and dried in vacuo to obtain complex **2** as bright yellow solid (0.394 g, 65%).^1^H NMR (300 MHz, C_6_D_6_): δ=7.61 (d, 2H, ArH), 7.60 (s, 2H, CH=N), 7.53 (br s, 2H, Ph), 7.34 (br s, 4H, ArH), 7.06 (d, 2H, ArH), 1.31 (s, 18H, *tert‐*Bu), 1.07 (s, 18H, *tert‐*Bu). ^13^C NMR (75 MHz, C_6_D_6_): δ=171.30 (C=N), 161.08 (Ar−O), 154.53, 143.47, 139.85 (q‐C), 133.01, 132.57 (CF_3_), 130.54 (ArH), 125.14 (q‐C), 123.50 (br, 2x Ph), 121.52 (br, Ph), 121.06 (q‐C), 35.20, 34.47, 31.35, 29.24 (*tert‐*Bu). ^19^F NMR (282 MHz, C_6_D_6_): δ=−62.75 (s, 6F, CF_3_). IR (ATR, cm^−1^): υ=2960 (m), 1598 (s, C=N), 1475 (s), 1364 (s), 1276 (s), 1179 (s), 1134 (s), 935 (m), 916 (m), 893 (s, Mo=O), 847 (s), 682 (m), 555 (m, Mo−O). Anal. calcd for C_46_H_48_F_12_MoN_2_O_4_: C, 54.34; H, 4.76; N, 2.76; Found: C, 54.21; H, 4.97; N, 2.72.

**Synthesis of Complex 3 [MoO_2_(L3)_2_]**. [MoO_2_Cl_2_] (1 equiv, 0.52 g, 2.63 mmol) was suspended in acetonitrile (20 mL), whereupon **HL3** (2.1 equiv, 1.40 g, 5.26 mmol) and NEt_3_ (2.4 equiv, 0.88 mL, 6.31 mmol) were added under stirring. The addition was accompanied by a color change from bright yellow to deep red. The reaction mixture was subsequently stirred overnight at room temperature, whereupon a beige precipitate had formed. The precipitate was filtered off, washed with cold acetonitrile (3×15 mL) and pentane (2×10 mL) and dried in vacuo to obtain **3** as bright yellow solid (1.34 g, 78%). Single crystals of **3** suitable for X‐ray diffraction analysis were obtained via slow evaporation from a concentrated solution of **3** in dichloromethane layered with n‐heptane. ^1^H NMR (300 MHz, (CD_3_)_2_SO): δ=8.51 (s, 2H, CH=N), 7.88–7.11 (m, 14H, ArH+Ph). ^13^C NMR (300 MHz, (CD_3_)_2_SO): δ=167.41, 152.88, 150.33, 134.10, 132.71, 132.26, 130.72, 129.65, 128.81, 128.32, 126.66, 124.70, 123.90, 123.32, 123.06, 122.58, 121.53. IR (ATR, cm^−1^): υ=1613 (s, C=N), 1444 (s), 1375 (m), 1279 (s), 1177 (s), 916 (s), 900 (s, Mo=O), 873 (s), 857 (s), 783 (s), 733 (s), 699 (s), 608 (s), 543 (s, Mo−O), 509 (s), 483 (s), 463 (s). Anal. calcd for C_26_H_16_Cl_4_MoN_2_O_4_ ⋅ 0.2 C_5_H_12_: C, 48.21; H, 2.76; N, 4.16; Found: C, 48.48; H, 2.80; N, 4.16.

**Synthesis of Complex 4 [MoO{OB(C_6_F_5_)_3_}(L1)_2_]**. A solution of B(C_6_F_5_)_3_ (1 equiv, 0.068 g, 0.13 mmol) in dry pentane (2 mL) was added to a suspension of complex **1** (1 equiv, 0.1 g, 0.13 mmol) in the same solvent (3 mL). The addition was accompanied by an immediate color change of the suspension from yellow to dark red. The reaction mixture was subsequently stirred at room temperature for 6 h, whereupon a large quantity of a dark red precipitate had formed. The precipitate was subsequently filtered off, washed thoroughly with cold pentane (3×5 mL) and dried in vacuo to yield complex **4** as a dark red solid (0.153 g, 91%). Single crystals suitable for X‐ray diffraction analysis were obtained via vapor diffusion of pentane into a saturated toluene solution of **4** at room temperature. ^1^H NMR (300 MHz, C_6_D_6_): δ=7.76 (s, 1H, CH=N), 7.56 (d+s, 2H, ArH+CH=N), 7.44 (d, 1H, ArH), 6.95–6.82 (m, 5H, Ph), 6.78 (d, 1H, ArH), 6.75 (d, 1H, ArH), 6.62–6.50 (m, 5H, Ph), 1.23 (s, 9H, *tert‐*Bu), 1.10 (s, 9H, *tert‐*Bu), 1.06 (s, 9H, *tert‐*Bu), 1.03 (s, 9H, *tert‐*Bu). ^13^C NMR (75 MHz, C_6_D_6_): δ=171.64, 169.14 (C=N), 159.36, 155.36 (Ar−O), 152.46, 151.20 (q‐C), 149.97, 146.81 (C_6_F_5_), 146.48, 145.18 (q‐C), 141.84 (C_6_F_5_), 139.35, 139.17 (q‐C), 135.74 (C_6_F_5_), 133.06, 132.92 130.48, 129.89 (ArH), 128.84, 128.73, 127.36, 127.24, 124.04, 123.65 (Ph), 123.46, 121.69 (q‐C), 35.46, 35.22, 34.50, 34.36 (q‐*tert‐*Bu), 31.21, 31.16, 30.74, 29.56 (*tert‐*Bu). ^19^F NMR (282 MHz, C_6_D_6_): δ=−130.25 (dd, 6F, *o*‐F), −158.78 (t, 3F, *p*‐F), ‐165.03 (m, 6F, *m*‐F). IR (ATR, cm^−1^): υ=2962 (m, C−H), 1606 (w, C=N), 1514 (m), 1467 (s), 1235 (m), 1094 (s), 977 (s), 880 (s), 843 (s), 765 (s), 555 (s, Mo−O). Anal. calcd for C_60_H_52_BF_15_MoN_2_O_4_: C, 57.34; H, 4.17; N, 2.23; Found: C, 57.14; H, 4.08; N, 2.23.

**Synthesis of Complex 5 [Mo{OB(C_6_F_5_)_3_}O(L2)_2_]**. A solution of B(C_6_F_5_)_3_ (1 equiv, 0.025 g, 0.05 mmol) in dry pentane (1 mL) was added to a suspension of complex **2** (1 equiv, 0.05 g, 0.05 mmol) in the same solvent (2 mL). The addition was accompanied by an immediate color change of the suspension from yellow to deep red and by the formation of a dark red precipitate. The reaction mixture was subsequently stirred at room temperature for 6 h, the precipitate was filtered off, washed thoroughly with cold pentane (3×5 mL) and dried in vacuo to yield complex **5** as a dark red‐brownish solid (0.063 g, 84%). Single crystals suitable for X‐ray diffraction analysis were obtained from a saturated pentane solution of **5** at −35 °C. ^1^H NMR (300 MHz, C_6_D_6_): δ =7.58 (d, 2H, ArH), 7.54 s, 2H, CH=N), 7.51 (br s, 2H, Ph), 7.26 (br s, 4H, Ph), 7.03 (d, 2H, ArH), 1.26 (s, 18H, *tert‐*Bu), 0.91 (s, 18H, *tert‐*Bu). ^13^C NMR (75 MHz, C_6_D_6_, C_6_F_5_ obscured): δ=171.99 (C=N), 159.22 (Ar−O), 153.85, 145.45, 139.63 (q‐C), 134.05 (ArH), 133.07 (q, ^1^J_C‐F_=33.9 Hz, CF_3_), 130.98 (ArH), 124.90 (q‐C), 123.53 (br, 2 Ph), 121.28 (q‐C), 121.16 (Ph), 35.09, 34.55 (q‐*tert‐*Bu), 31.13, 29.33 (*tert‐*Bu). ^19^F NMR (282 MHz, C_6_D_6_): δ=−62.81 (s, 12F, CF_3_), −132.31 (d, 6F, *o*‐F), −148.23 (br s, 3F, *p*‐F), −161.03 (m, 6F, *m*‐F). IR (ATR, cm^−1^): υ=2962 (m, C−H), 1596 (w, C=N), 1517 (m), 1467 (s), 1365 (s), 1278 (m), 1176 (m), 1141 (s), 1094 (s), 978 (s), 880 (s), 847 (s), 762 (m), 682 (s), 560 (s, Mo−O). Anal. calcd for C_64_H_48_BF_27_MoN_2_O_4_ ⋅ 0.5 C_5_H_12_: C, 51.04; H, 3.48; N, 1.79; Found: C, 50.99; H, 3.84; N, 1.75.

**Synthesis of Complex 6 [Mo{OB(C_6_F_5_)_3_}O(L3)_2_]**. A solution of B(C_6_F_5_)_3_ (1 equiv, 0.2 g, 0.39 mmol) in dry pentane (10 mL) was added to a suspension of complex **3** (1 equiv, 0.26 g, 0.39 mmol) in the same solvent (10 mL). The addition was accompanied by an immediate color change from yellow to deep red and by the formation of a red precipitate. The reaction mixture was subsequently stirred at room temperature overnight, the precipitate was filtered off, washed thrice with cold pentane (3×10 mL) and dried in vacuo to yield **6** as a brick red solid (0.35 g, 76%). Single crystals suitable for X‐ray diffraction analysis were obtained from a concentrated benzene solution of **6** at room temperature or a concentrated toluene solution at −35 °C. ^1^H NMR (300 MHz, CD_2_Cl_2_): δ=8.24 (s, 1H, CH=N), 8.09 (s, 1H, CH=N), 7.40–7.35 (m, 2H, ArH), 7.27–7.07 (br m, 10H, Ph), 6.72–6.69 (m, 2H, ArH). ^13^C NMR (75 MHz, CD_2_Cl_2_): δ=166.71, 166.46, 153.10, 152.40, 150.38, 150.06, 146.77, 141.95, 139.32, 138.47, 137.54, 136.08, 135.88, 135.57, 132.57, 132.29, 129.76, 129.52, 128.90, 128.50, 128.04, 126.15, 125.98, 123.83, 123.41, 123.02, 122.38. ^19^F NMR (282 MHz, CD_2_Cl_2_): δ=−132.0 (d, 6F, *o*‐F), −158.97 (t, 3F, *p*‐F), −164.93 (m, 6F, *m*‐F). IR (ATR, cm^−1^): υ=1610 (m, C=N), 1546 (m), 1465 (s), 1446 (s), 1269 (s), 1093 (s), 976 (s), 882 (s), 790 (s), 732 (m), 701 (m), 671 (m), 609(m), 551 (s, Mo−O), 520 (m). Anal. calcd for C_44_H_16_BCl_4_F_15_MoN_2_O_4_: C, 45.16; H, 1.38; N, 2.39; Found: C, 45.07; H, 1.42; N, 2.58.

**Procedure for Catalytic Runs**. All catalytic experiments for determination of conversion using Gas Chromatography were performed under inert conditions (N_2_ atmosphere, exclusion of moisture) in Mininert® reaction vessels. In a typical experiment, an aliquot of a chlorobenzene stock solution of the respective catalyst was added to 0.5 mL of chlorobenzene containing 0.1 mmol of the substrate, two equivalents of silane and five equivalents of the respective halide. 0.1 mmol of mesitylene was used as internal standard. Samples for GC‐MS measurements were withdrawn at given time intervals with a microliter syringe (10 μL), quenched with Na_2_CO_3_ and diluted by a factor of 50 with HPLC grade ethyl acetate. A 0 h sample was withdrawn before addition of the silane. All catalytic experiments for determination of isolated yields of products **7 a**–**7 l** were performed under inert conditions using Schlenk techniques. 0.01–0.03 mmol of the catalyst was weighted in the glovebox in a Schlenk flask and dissolved in 5 mL chlorobenzene, then 1 mmol of the substrate, 2 mmol PhSiH_3_ and 5 mmol of the respective halide were added. After stirring at 50 °C for the corresponding reaction time, the reaction was quenched with aqueous sodium carbonate, the solvent and volatiles were removed under vacuum, the residue re‐dissolved in dichloromethane and filtered over a plug of silica. Isolated yields were obtained by purifying the products using column chromatography over silica with a Biotage Isolera Four equipment, using cyclohexane/ethyl acetate mixtures (10:1) as the eluent.

**Analytical data for (3,3‐dichloropropyl)benzene 7 a**. Using 0.1 g **7 a’** as substrate, **7 a** was isolated in 79% yield (0.067 g). NMR data are supported by previous publication.^31^

^1^H NMR (300 MHz, CDCl_3_): δ=7.34–7.20 (m, 5H, ArH), 5.66 (t, 1H, ^1^J_C‐H_=6.1 Hz, −CHCl_2_), 2.91–2.86 (m, 2H, Ar−CH_2_), 2.55–2.48 (m, 2H, −CH_2_).

**Analytical data for (3,3‐dibromopropyl)benzene 7 b**. Using 0.1 g styrene as substrate, **7 b** was isolated in 86% yield (0.23 g). NMR data are supported by previous publication.^32^ ^1^H NMR (300 MHz, CDCl_3_): δ=7.35–7.20 (m, 5H, ArH), 5.60 (t, 1H, ^1^J_C‐H_=6.3 Hz, −CHBr_2_), 2.89–2.85 (m, 2H, Ar−CH_2_), 2.74–2.67 (m, 2H, −CH_2_).

**Analytical data for (3,3‐dibromopropane‐1,1‐diyl)dibenzene 7 c**. Using 0.18 g 1,1‐diphenylethylene as substrate, **7 c** was isolated in 90% yield (0.318 g). ^1^H NMR (300 MHz, CDCl_3_): δ=7.36–7.22 (m, 10H, ArH), 5.30 (t, 1H, ^1^J_C‐H_=6.9 Hz, −CHBr_2_), 4.28 (t, 1H, ^1^J_C‐H_=7.7 Hz, Ar_2_CH), 3.16–3.11 (dd, 2H, ^1^J_C‐H_=6.9 Hz, ^1^J_C‐H_=7.7 Hz, −CH_2_). ^13^C NMR (75 MHz, CDCl_3_): δ=142.20, 129.00, 127.91, 127.07, 51.05, 49.95, 44.38.

**Analytical data for (4,4‐dibromobutan‐2‐yl)benzene 7 d**. Using 0.118 g α‐methylstyrene as substrate, **7 d** was isolated in 96% yield (0.278 g). ^1^H NMR (300 MHz, CDCl_3_): δ=7.35–7.20 (m, 5H, ArH), 5.27–5.22 (m, 1H, −CHBr_2_), 3.11–2.99 (m, 1H, ArCHMe), 2.69–2.63 (m, 2H, −CH_2_), 1.31 (d, 1H, ^1^J_C‐H_=7.0 Hz, −CH_3_). ^13^C NMR (75 MHz, CDCl_3_): 144.05, 128.98, 127.13, 126.99, 53.72, 44.72, 39.29, 21.80.

**Analytical data for (3,5,5‐tribromopentyl)benzene 7 e**. Using 0.132 g 4‐phenyl‐1‐butene as substrate, **7 e** was isolated in 42% yield (0.16 g). ^1^H NMR (300 MHz, CDCl_3_): δ=7.34–7.21 (m, 5H, ArH), 5.93–5.89 (m, 1H, −CHBr_2_), 4.16–4.07 (m, 1H, CHBr), 2.98–2.74 (m, 4H, −CH_2_), 2.22–2.13 (m, 2H, −CH_2_). ^13^C NMR (75 MHz, CDCl_3_): 140.34, 128.75, 128.59, 126.48, 53.69, 53.52, 43.35, 40.21, 33.55.

**Analytical data for 1,1,3‐tribromononane 7 f**. Using 0.112 g 1‐octene as substrate, **7 f** was isolated in 41% yield (0.149 g). NMR data are supported by previous publication.^33^ ^1^H NMR (300 MHz, CDCl_3_): δ=5.92–5.88 (m, 1H, −CHBr_2_), 4.16–4.08 (m, 1H, CHBr), 2.84–2.76 (m, 2H, −CH_2_), 1.89–1.84 (m, 2H,−CH_2_), 1.30 (bs, 8H, −CH_2_), 0.89 (t, 3H, ^1^J_C‐H_=6.6 Hz, −CH_3_).

**Analytical data for (3,3‐dibromo‐2‐methylpropyl)benzene 7 g**. Using 0.118 g *trans*‐β‐methylstyrene as substrate, **7 g** was isolated in 76% yield (0.22 g). ^1^H NMR (300 MHz, CDCl_3_): δ=7.36–7.20 (m, 5H, ArH), 5.73 (d, 1H, ^1^J_C‐H_=2.6 Hz, −CHBr_2_), 2.88–2.81 (dd, 1H, ^1^J_C‐H_=13.7, 7.3 Hz, −CH_2_), 2.63 (dd, 1H, ^1^J_C‐H_=13.7, 7.3 Hz, −CH_2_), 2.44–2.32 (m, 1H, −CHMe), 1.20 (d, 3H, ^1^J_C‐H_=6.5 Hz, −CH_3_). ^13^C NMR (75 MHz, CDCl_3_): δ=138.85, 129.17, 128.78, 126.73, 54.53, 46.83, 40.62, 16.58.

**Analytical data for (3,3‐dibromopropane‐1,2‐diyl)dibenzene 7 h**. Using 0.18 g cis‐stilbene as substrate, **7 h** was isolated in 18% yield (0.064 g). ^1^H NMR (300 MHz, CDCl_3_): δ=7.41–7.10 (m, 10H, ArH), 5.85 (d, 1H, ^1^J_C‐H_=4.5 Hz, −CHBr_2_), 3.60–3.56 (m, 1H, −CHPh), 3.48–3.41 (m, 1H, −CH_2_), 3.17–3.10 (m, 1H, −CH_2_). ^13^C NMR (75 MHz, CDCl_3_): δ=138.61, 138.58, 129.40, 129.16, 128.61, 128.31, 128.01, 126.65, 58.14, 51.64, 38.87.

**Analytical data for 2‐(dibromomethyl)‐1,2,3,4‐tetrahydronaphthalene 7 i**. Using 0.13 g 1,2‐dihydronaphtalene as substrate, **7 i** was isolated in 46% yield (0.14 g). ^1^H NMR (300 MHz, CDCl_3_): δ=7.14–7.12 (m, 4H, ArH), 5.87 (d, 1H, ^1^J_C‐H_=4.2 Hz, −CHBr_2_), 3.12–2.82 (m, 4H, −CH_2_), 2.45–2.32 (m, 1H, CH_2_), 2.21–2.12 (m, 1H, CH_2_), 1.80–1.65 (m, 1H, CH). ^13^C NMR (75 MHz, CDCl_3_): δ=135.87, 134.79, 129.38, 128.83, 126.16, 126.09, 53.12, 46.18, 32.97, 28.92, 27.60.

**Analytical data for 1‐(*tert*‐butyl)‐4‐(3,3‐dibromopropyl)benzene 7 j**. Using 0.16 g 4‐*tert‐*butylstyrene as substrate, **7 j** was isolated in 90% yield (0.3 g). ^1^H NMR (300 MHz, CDCl_3_): δ=7.35–7.32 (m, 2H, Ar), 7.16–7.13 (m, 2H, Ar), 5.60 (t, 1H, ^1^J_C‐H_=6.4 Hz, −CHBr_2_), 2.86–2.81 (m, 2H, CH_2_), 2.73–2.66 (m, 2H, CH_2_), 1.32 (s, 9H, *tert‐*Bu). ^13^C NMR (75 MHz, CDCl_3_): δ=149.56, 136.11, 128.35, 125.73, 46.90, 45.57, 34.58, 33.70, 31.51.

**Analytical data for 1‐bromo‐4‐(3,3‐dibromopropyl)benzene 7 k**. Using 0.183 g 4‐bromostyrene as substrate, **7 k** was isolated in 90% yield (0.32 g). ^1^H NMR (300 MHz, CDCl_3_): δ=7.45–7.42 (m, 2H, Ar), 7.10–7.07 (m, 2H, Ar), 5.58 (t, 1H, ^1^J_C‐H_=6.2 Hz, −CHBr_2_), 2.85–2.80 (m, 2H, CH_2_), 2.70–2.63 (m, 2H, CH_2_). ^13^C NMR (75 MHz, CDCl_3_): δ=138.18, 131.92, 130.42, 120.52, 46.55, 44.82, 33.64.

**Analytical data for 4,4′‐oxybis((3,3‐dibromo‐2‐methylpropyl)benzene) 7 l**. Using 0.148 g anethole as substrate, **7 l** was isolated in 18% yield (0.107 g). ^1^H NMR (300 MHz, CDCl_3_): δ=7.08–7.05 (m, 4H, ArH), 6.80–6.77 (m, 4H, ArH), 5.71 (d, 2H, ^1^J_C‐H_=2.6 Hz, −CHBr_2_), 2.77–2.70 (dd, 2H, ^1^J_C‐H_=13.8, 7.3 Hz, −CH_2_), 2.58–2.51 (dd, 2H, ^1^J_C‐H_=13.8, 7.3 Hz, −CH_2_), 2.35–2.23 (m, 2H, −CHMe), 1.17 (d, 6H, ^1^J_C‐H_=6.5 Hz, −CH_3_). ^13^C NMR (75 MHz, CDCl_3_): 154.30, 131.01, 130.32, 115.60, 54.60, 46.97, 39.76, 16.48. ESI‐MS (135 V): *m/z*=592.8 [M−H]^−^.

**Crystallographic Data for Complexes 1–6**. The X‐ray data collections were performed with a Bruker AXS SMART APEX‐II CCD diffractometer at 100 K with Mo‐*K*_α_ radiation (λ=0.71073 Å) from an Incoatec microfocus sealed tube equipped with a multilayer monochromator. Absorption corrections were made semi‐empirically from equivalents. The structures were solved by direct methods (SHELXS‐97) and refined by full‐matrix least‐squares techniques against *F* ^2^ (SHELXL‐2014/6). A weighting scheme of w=1/[σ^2^(F_o_^2^)+(aP)^2^+bP] where P=(F_o_^2^+2F_c_^2^)/3 was used. The absolute configuration of **3** was established by anomalous dispersion effects in the diffraction measurements of the crystal. The non‐hydrogen atoms of **1**, **3**, **4**, and **6** were refined with anisotropic displacement parameters without any constraints. The H atoms of the phenyl rings including any adjacent CH=N groups were put at the external bisectors of the C−C−C angles at C−H distances of 0.95 Å and common isotropic displacement parameters were refined for the H atoms of the same ring. The H atoms of the *tert*‐butyl groups were refined with common isotropic displacement parameters for the H atoms of the same group and idealized geometries with tetrahedral angles, enabling rotations around the C−C bonds, and C−H distances of 0.98 Å. Crystallographic data for the structures of compounds **1**–**6** have been deposited with the Cambridge Crystallographic Data Center (CCDC 1940999 to CCDC 1941004 for **1** to **6**).

## Supporting information

As a service to our authors and readers, this journal provides supporting information supplied by the authors. Such materials are peer reviewed and may be re‐organized for online delivery, but are not copy‐edited or typeset. Technical support issues arising from supporting information (other than missing files) should be addressed to the authors.

Supplementary
