# Supplementary material for: Hydroalkylation of Aryl Alkenes with Organohalides Catalyzed by Molybdenum Oxido Based Lewis Pairs
Source: Adv Synth Catal. 2020 Jun 29;362(15):3170–82. doi: 10.1002/adsc.202000425 (PMC7497237; doi:10.1002/adsc.202000425)
Supplement: Supplementary file 1 — Supplementary [file ADSC-362-3170-s001.pdf]

Supporting Information

Hydroalkylation of Aryl Alkenes with Organohalides Catalyzed by Molybdenum Oxido Based Lewis Pairs

Niklas Zwettler, Antoine Dupé, Sumea Klokić, Angela Milinković, Dado Rodić, Simon Walg, Dmytro Neshchadin, Ferdinand Belaj, and Nadia C. Mösch-Zanetti*© 2020 The Authors. Published by Wiley-VCH Verlag GmbH & Co. KGaA. This is an open access article under the terms of the Creative Commons Attribution License, which permits use, distribution and reproduction in any medium, provided the original work is properly cited.

SUPPORTING INFORMATION

Hydroalkylation of Aryl Alkenes with Organohalides Catalyzed by Molybdenum-Oxido Based Lewis Pairs

*Niklas Zwettler,[a] Antoine Dupé,[a] Sumea Klokić,[a] Angela Milinković,[a] Dado Rodić,[a] Simon Walg,[a] Dmytro Neshchadin,[b] Ferdinand Belaj[a] and Nadia C. Mösch-Zanetti[a]**

a) Institute of Chemistry, University of Graz, Schubertstrasse 1, 8010 Graz, Austria

b) Institute for Physical and Theoretical Chemistry, Graz University of Technology, Stremayrgasse 9, 8010 Graz, Austria

* nadia.moesch@uni-graz.at

TABLE OF CONTENT

Figure S1. ^{1}H **NMR spectra of complex 2 in** C_6D_6 .... **S4**
Figure S2. ^{13}C **NMR spectra of complex 2 in** C_6D_6. .... **S4**
Figure S3. ^{19}F **NMR spectra of complex 2 in** C_6D_6. .... **S5**
Figure S4. ^{1}H **NMR spectra of complex 3 in** $(CD_3)_2SO$ **S5**
Figure S5. ^{13}C **NMR spectra of complex 3 in** $(CD_3)_2SO$. **S6**
Figure S6. ^{1}H **NMR spectra of complex 4 in** C_6D_6 .... **S6**
Figure S7. ^{13}C **NMR spectra of complex 4 in** C_6D_6. .... **S7**
Figure S8. ^{19}F **NMR spectra of complex 4 in** C_6D_6. .... **S7**
Figure S9. ^{1}H **NMR spectra of complex 5 in** C_6D_6 .... **S8**
Figure S10. ^{13}C **NMR spectra of complex 5 in** C_6D_6. .... **S8**
Figure S11. ^{19}F **NMR spectra of complex 5 in** C_6D_6. .... **S9**
Figure S12. ^{1}H **NMR spectra of complex 6 in** CD_2Cl_2 .... **S9**
Figure S13. ^{13}C **NMR spectra of complex 6 in** CD_2Cl_2. .... **S10**
Figure S14. ^{19}F **NMR spectra of complex 6 in** CD_2Cl_2. .... **S10**
Figure S15. ^{1}H **NMR spectra of 7a in** $CDCl_3$. **S11**
Figure S16. ^{1}H **NMR spectra of 7b in** $CDCl_3$. **S11**
Figure S17. ^{1}H **NMR spectra of 7c in** $CDCl_3$. **S12**
Figure S18. ^{13}C **NMR spectra of 7c in** $CDCl_3$ **S12**
Figure S19. ^{1}H **NMR spectra of 7d in** $CDCl_3$. **S13**
Figure S20. ^{13}C **NMR spectra of 7d in** $CDCl_3$. **S13**
Figure S21. ^{1}H **NMR spectra of 7e in** $CDCl_3$ **S14**
Figure S22. ^{13}C **NMR spectra of 7e in** $CDCl_3$ **S14**
Figure S23. ^{1}H **NMR spectra of 7f in** $CDCl_3$. **S15**
Figure S24. ^{1}H **NMR spectra of 7g in** $CDCl_3$. **S15**
Figure S25. ^{13}C **NMR spectra of 7g in** $CDCl_3$. **S16**
Figure S26. ^{1}H **NMR spectra of 7h in** $CDCl_3$. **S16**
Figure S27. ^{13}C **NMR spectra of 7h in** $CDCl_3$. **S17**
Figure S28. ^{1}H **NMR spectra of 7i in** $CDCl_3$. **S17**
Figure S29. ^{13}C **NMR spectra of 7i in** $CDCl_3$. **S18**
Figure S30. ^{1}H **NMR spectra of 7j in** $CDCl_3$ **S18**
Figure S31. ^{13}C **NMR spectra of 7j in** $CDCl_3$. **S19**
Figure S32. ^{1}H **NMR spectra of 7k in** $CDCl_3$. **S19**
Figure S33. ^{13}C **NMR spectra of 7k in** $CDCl_3$. **S20**
Figure S34. ^{1}H **NMR spectra of 7l in** $CDCl_3$. **S20**
Figure S35. ^{13}C **NMR spectra of 7l in** $CDCl_3$. **S21**

Figure S36. HSQC NMR spectra of 7l in $CDCl_3$. **S21**
Figure S37. X-band EPR spectrum of the reaction of complex 4 with $PhSiH_3$. **S22**
Crystallographic data for complexes 1-6. .... **S23**
Table S1. Crystal data and structure refinement details for 1-3 .... **S24**
Table S2. Crystal data and structure refinement details for 4-6 .... **S25**
Figure S37. Stereoscopic ORTEP plot of 1 .... **S26**
Table S3. Selected bond lengths [Å] and angles [°] for 1 .... **S26**
Figure S38. Stereoscopic ORTEP plot of 2 .... **S27**
Table S4. Selected bond lengths [Å] and angles [°] for 2 .... **S27**
Figure S39. Stereoscopic ORTEP plot of 3 .... **S28**
Table S5. Selected bond lengths [Å] and angles [°] for 3 .... **S28**
Figure S40. Stereoscopic ORTEP plot of 4 .... **S29**
Table S6. Selected bond lengths [Å] and angles [°] for 4 .... **S29**
Figure S41. Stereoscopic ORTEP plot of 5 .... **S30**
Table S7. Selected bond lengths [Å] and angles [°] for 5 .... **S30**
Figure S42. Stereoscopic ORTEP plot of 6 .... **S31**
Table S8. Selected bond lengths [Å] and angles [°] for 6 .... **S31**
REFERENCES **S32**

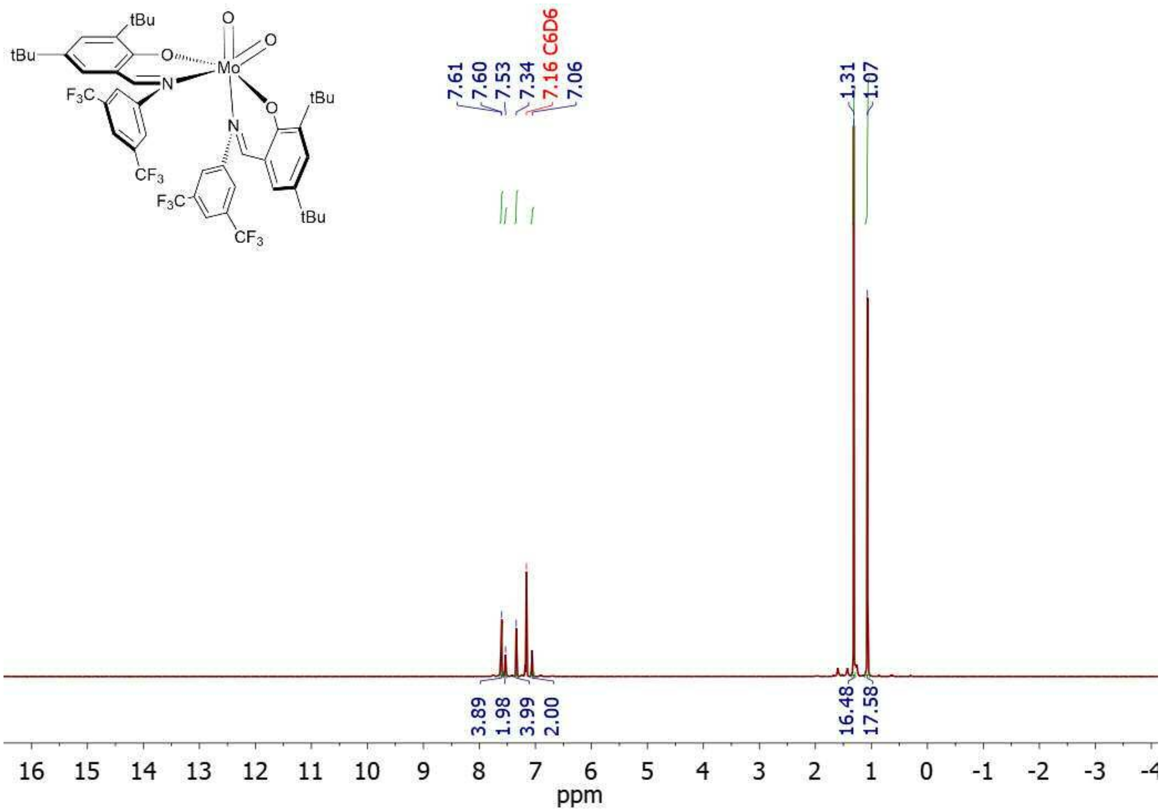

Figure S1. ^{1}H NMR spectra of complex **2** in C_6D_6.

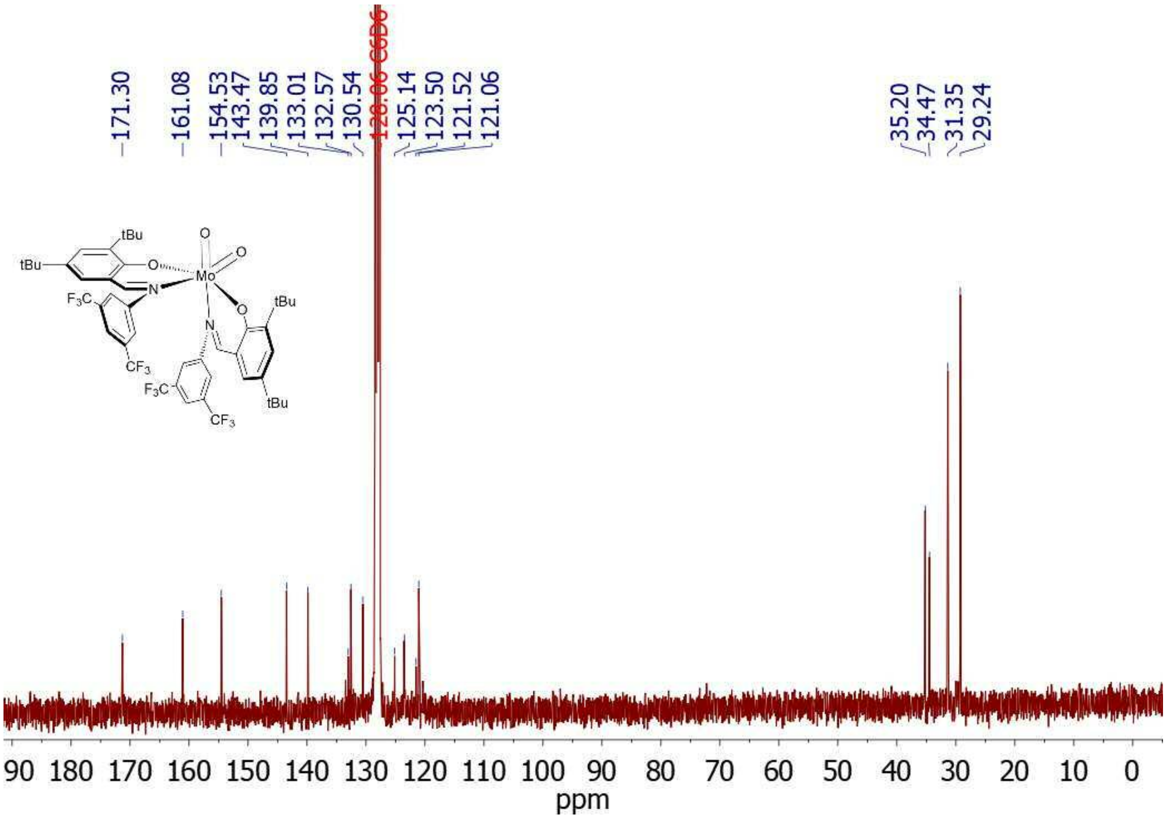

Figure S2. ^{13}C NMR spectra of complex **2** in C_6D_6.

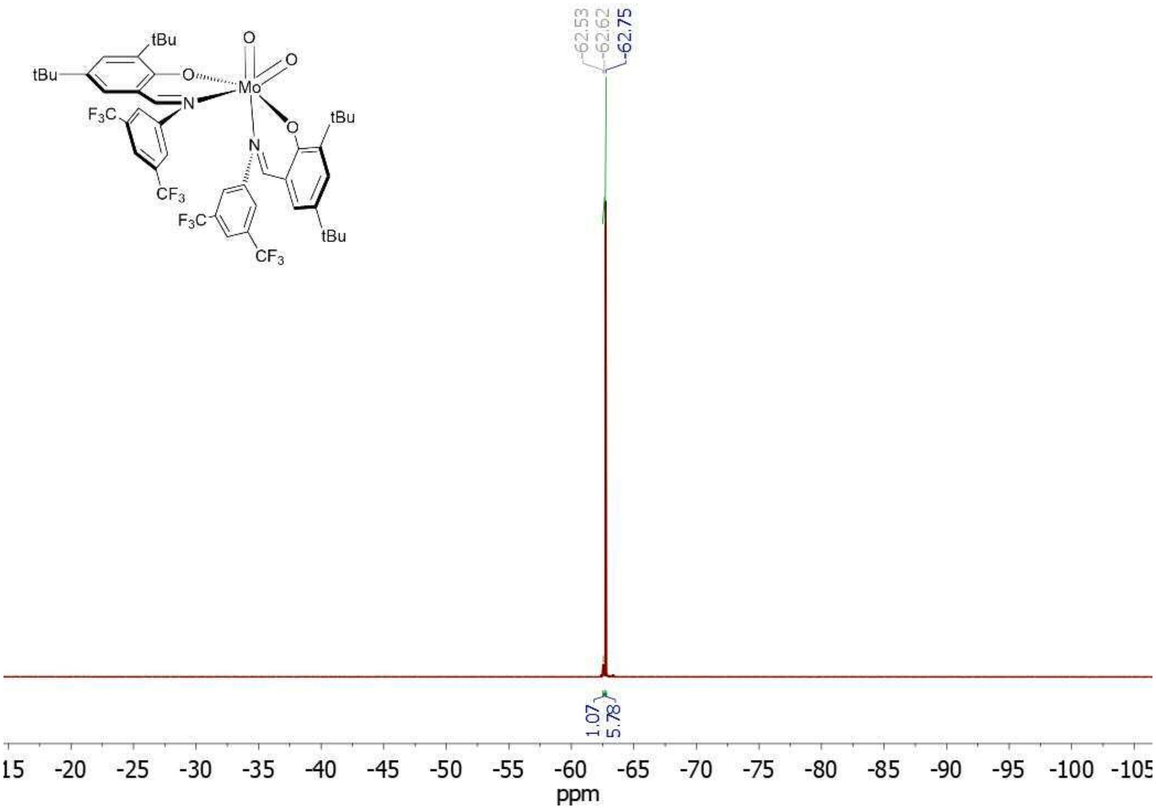

Figure S3. ^{19}F NMR spectra of complex **2** in C_6D_6.

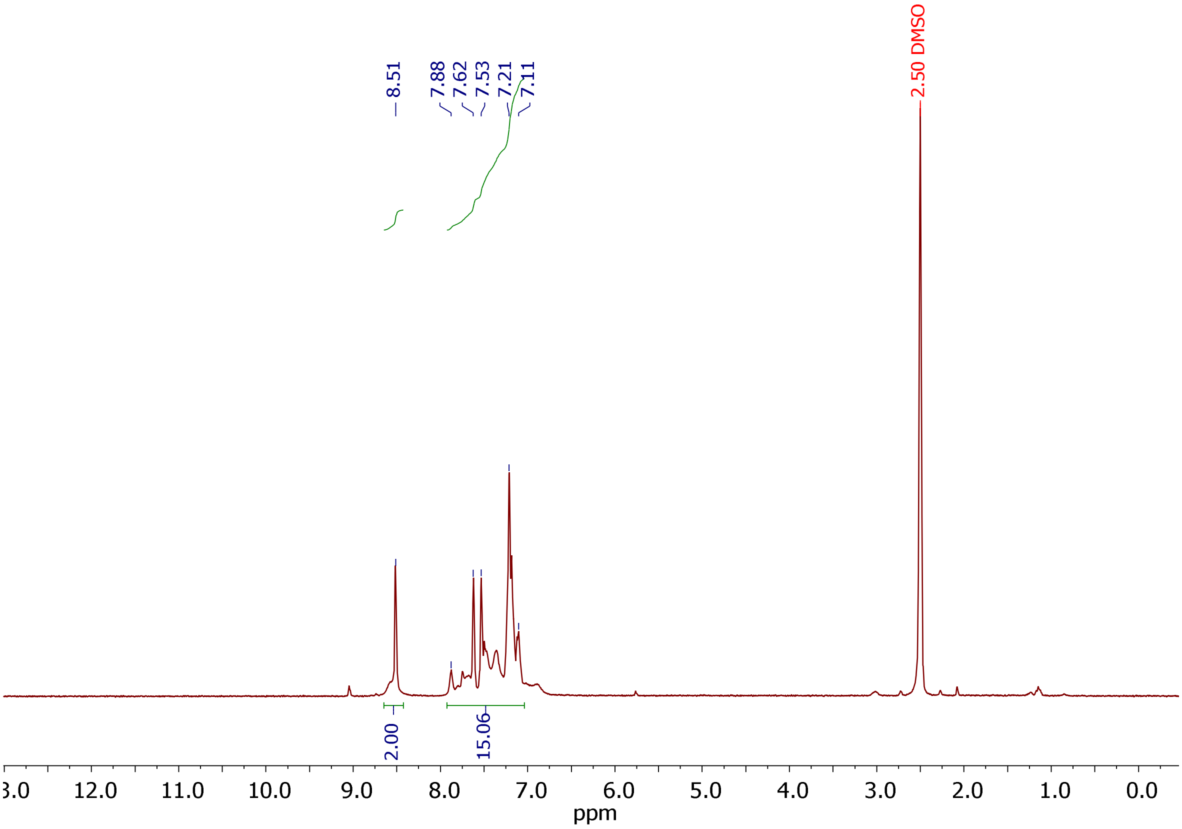

Figure S4. ^{1}H NMR spectra of complex **3** in $(CD_3)_2SO$.

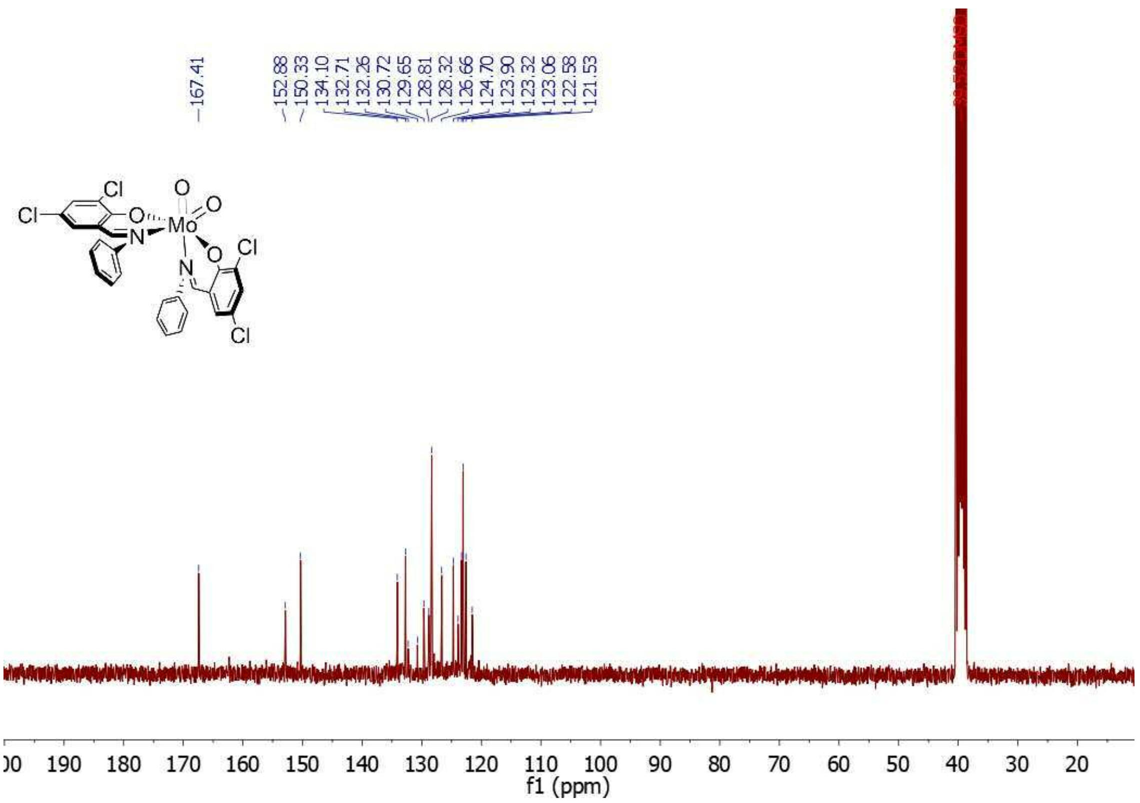

Figure S5. ^{13}C NMR spectra of complex **3** in $(CD_3)_2SO$.

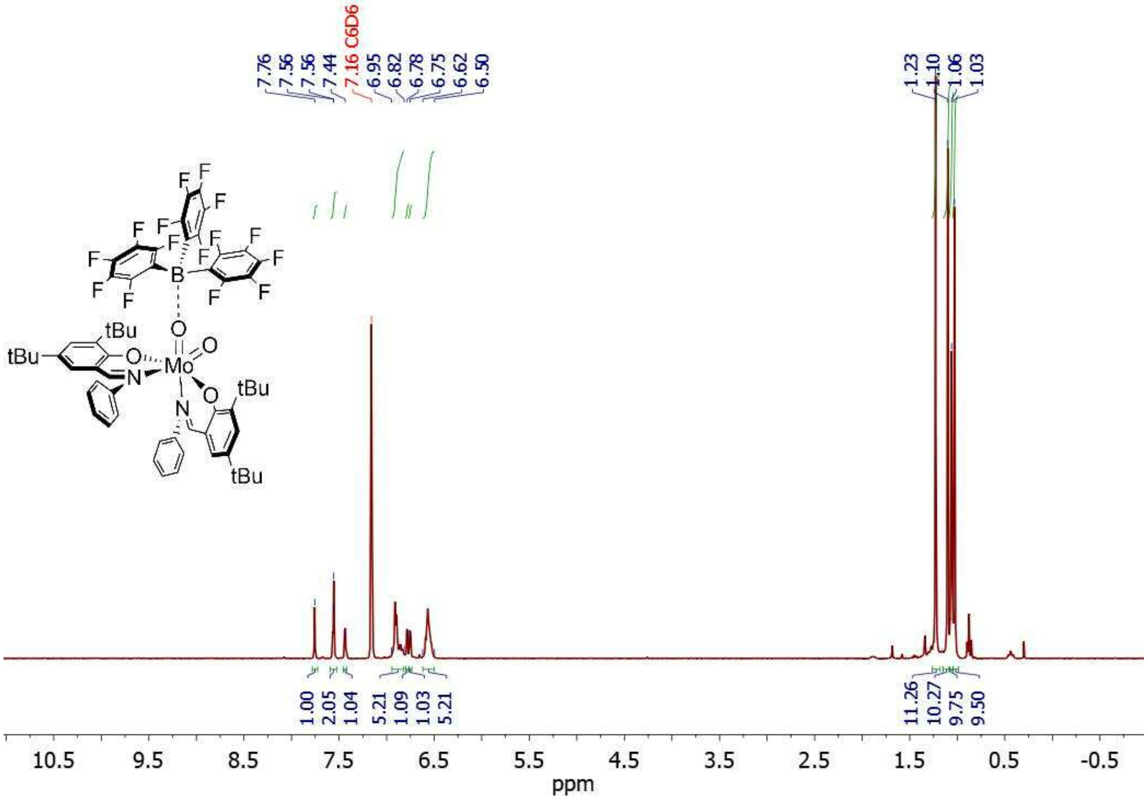

Figure S6. ^{1}H NMR spectra of complex **4** in C_6D_6.

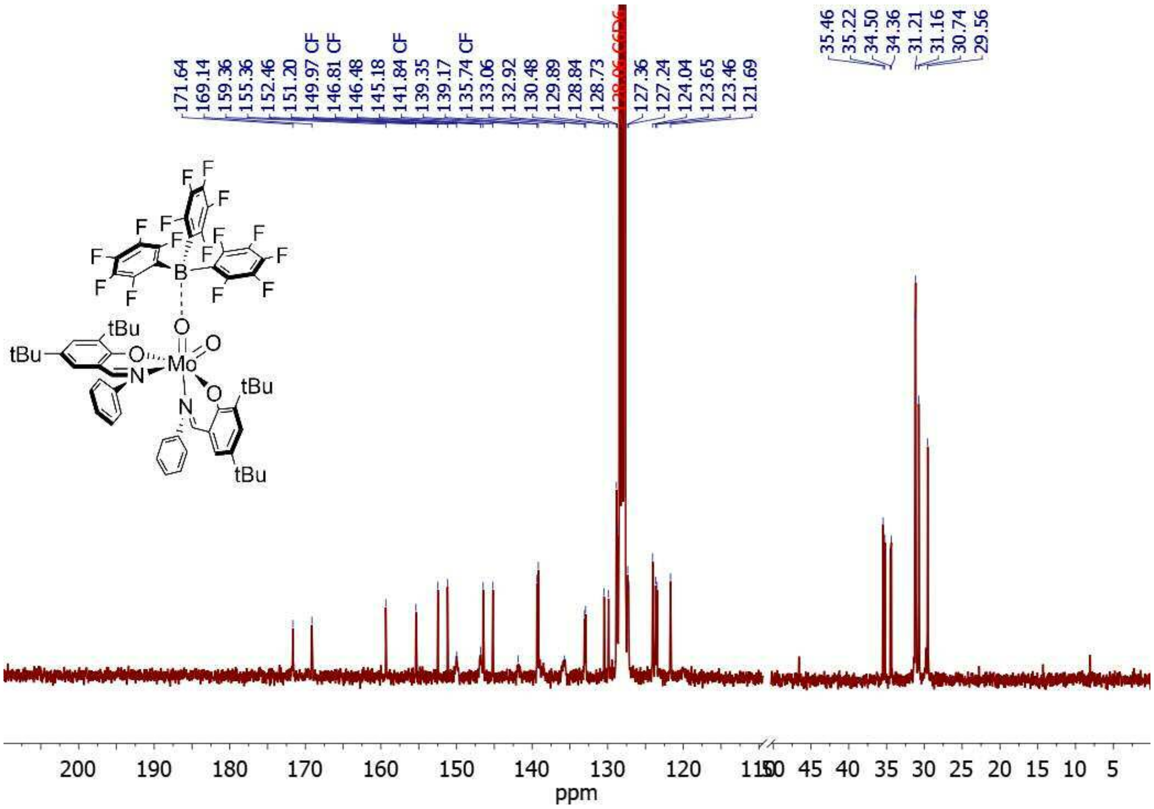

Figure S7. ^{13}C NMR spectra of complex **4** in C_6D_6.

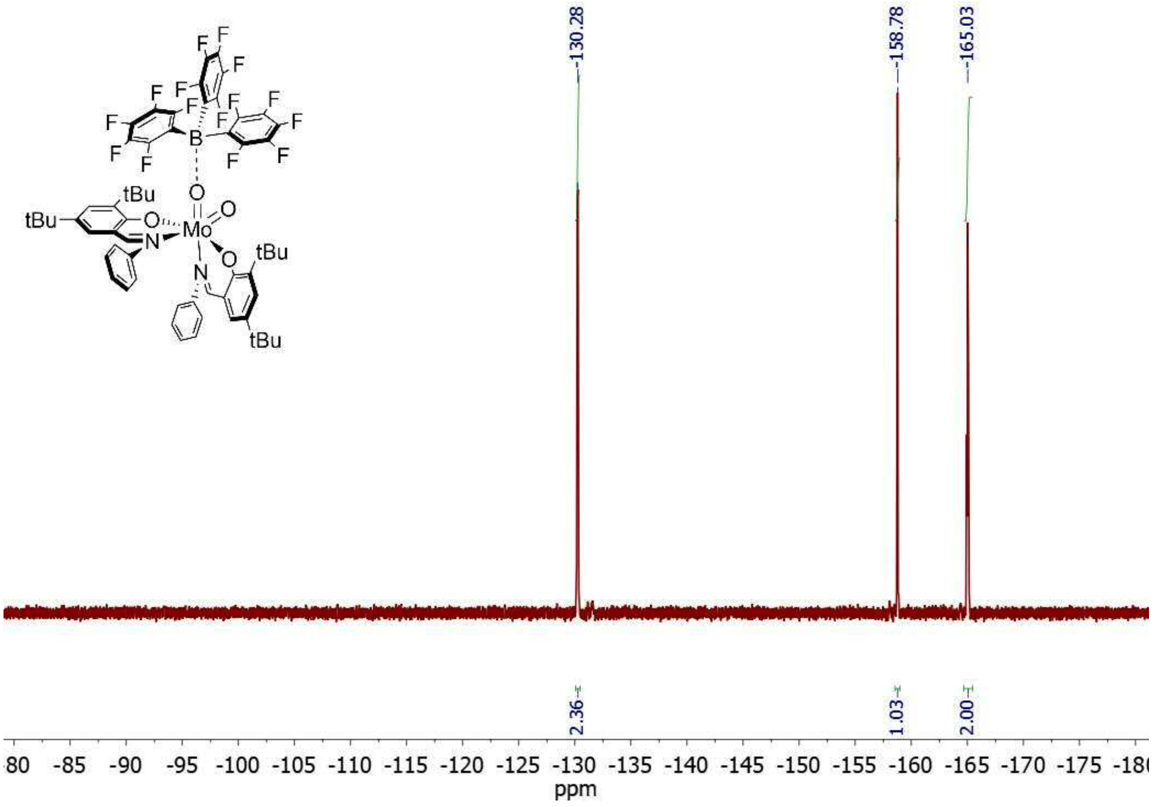

Figure S8. ^{19}F NMR spectra of complex **4** in C_6D_6.

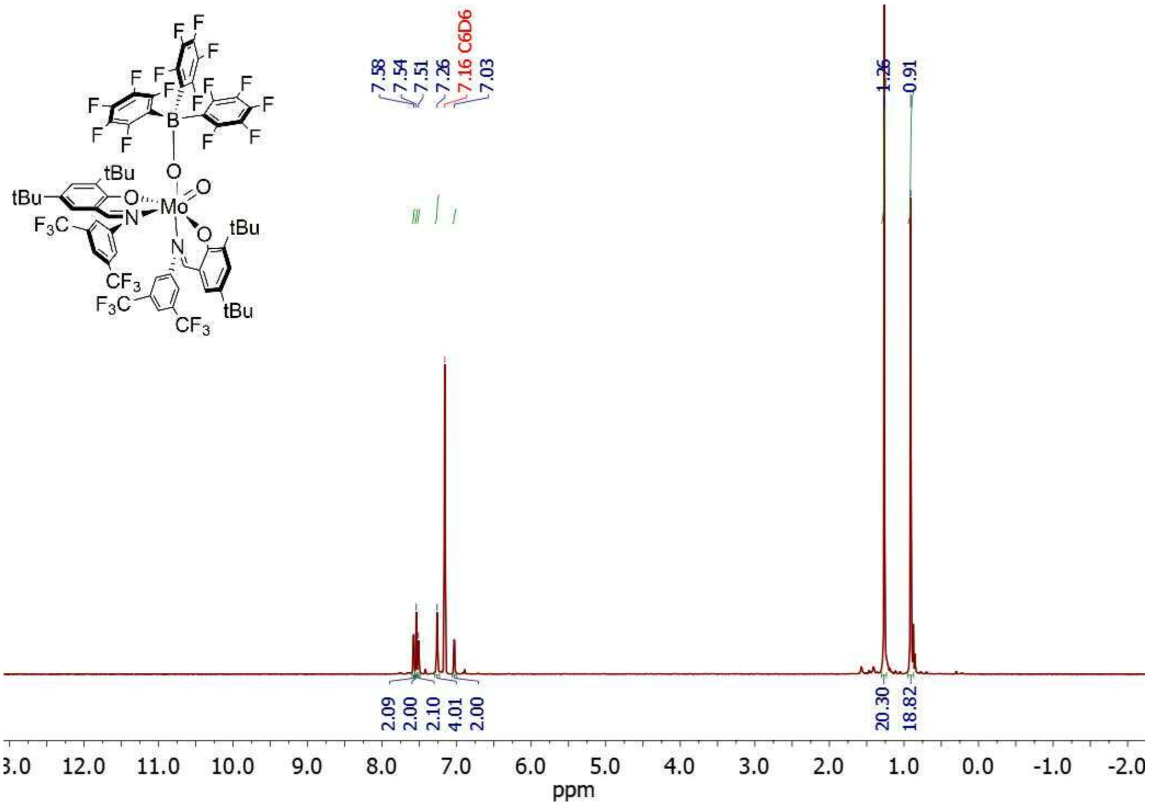

Figure S9. ^{1}H NMR spectra of complex **5** in C_6D_6.

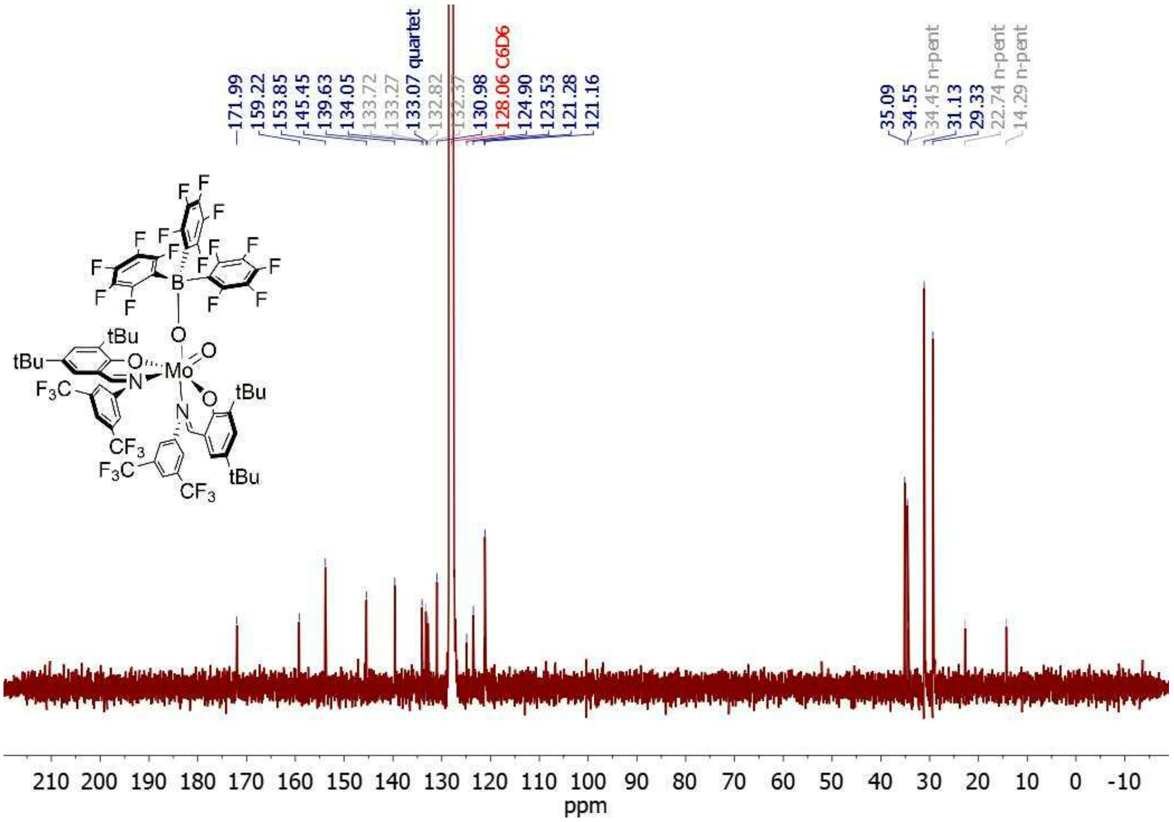

Figure S10. ^{13}C NMR spectra of complex **5** in C_6D_6.

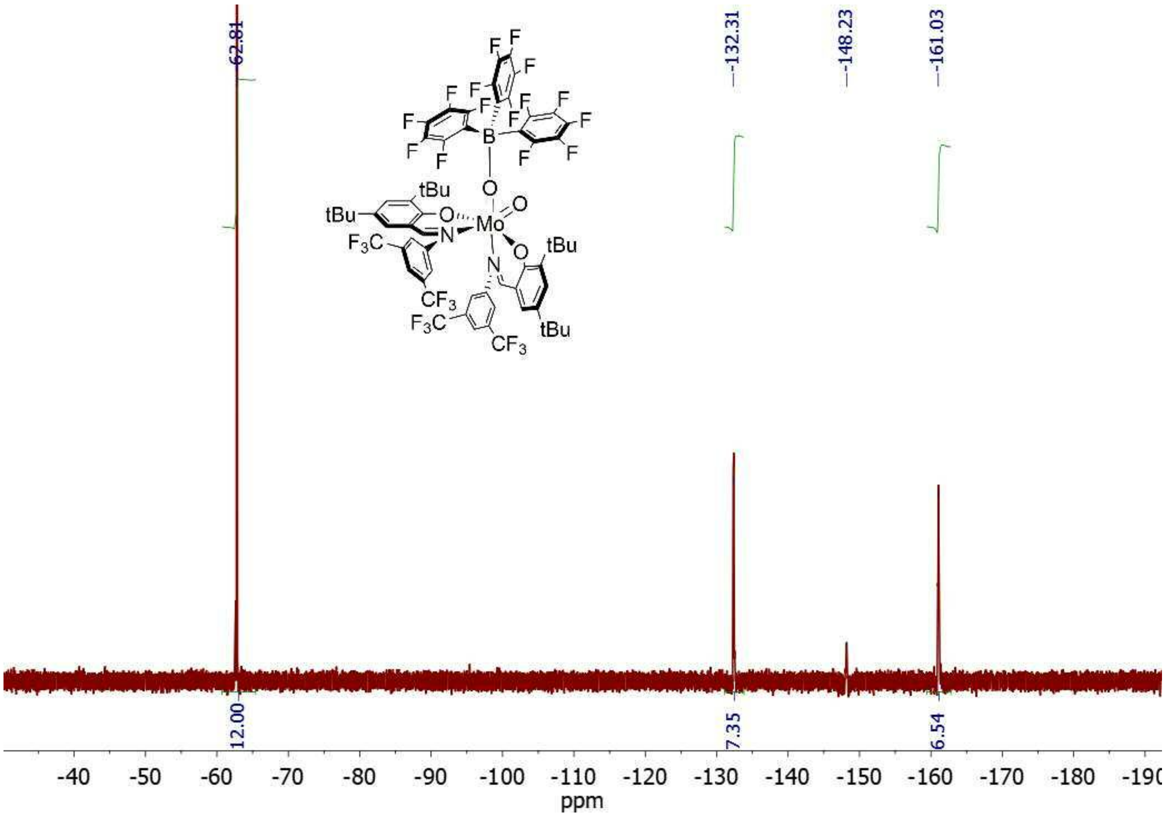

Figure S11. ${}^{19}F$ NMR spectra of complex **5** in C_6D_6.

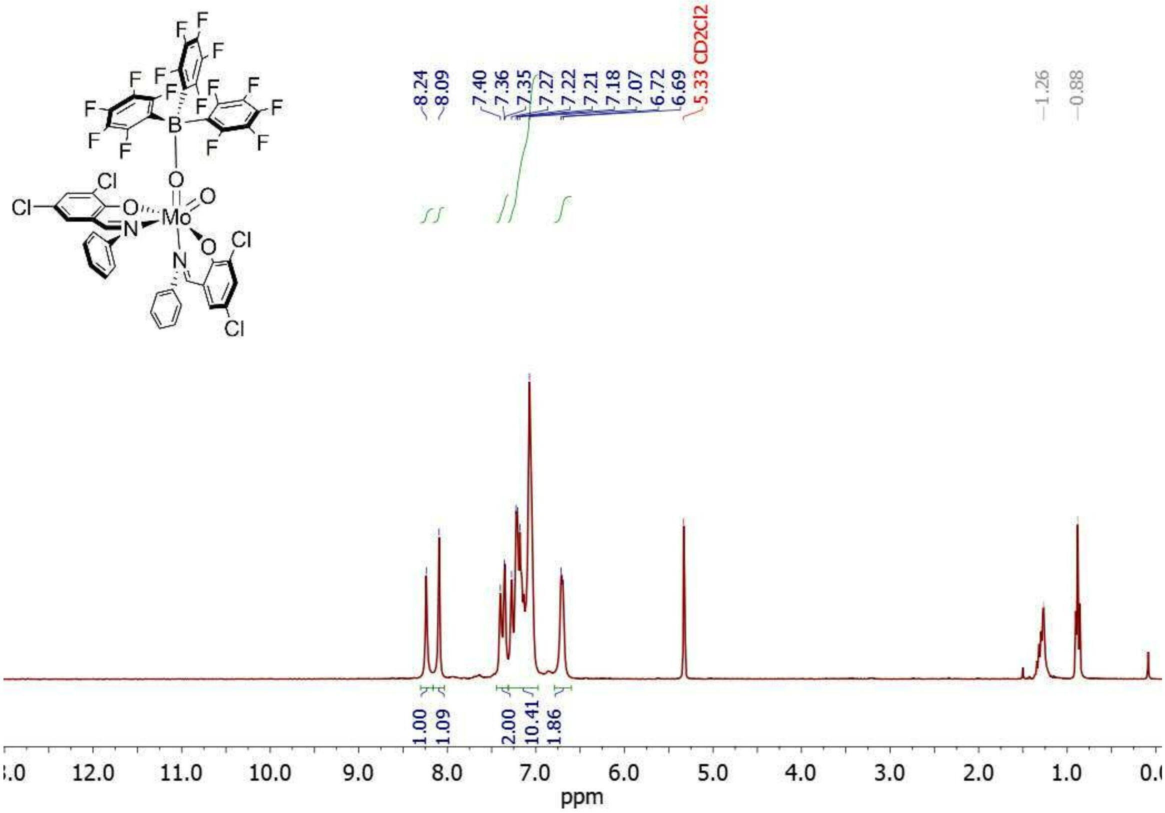

Figure S12. ${}^{1}H$ NMR spectra of complex **6** in CD_2Cl_2.

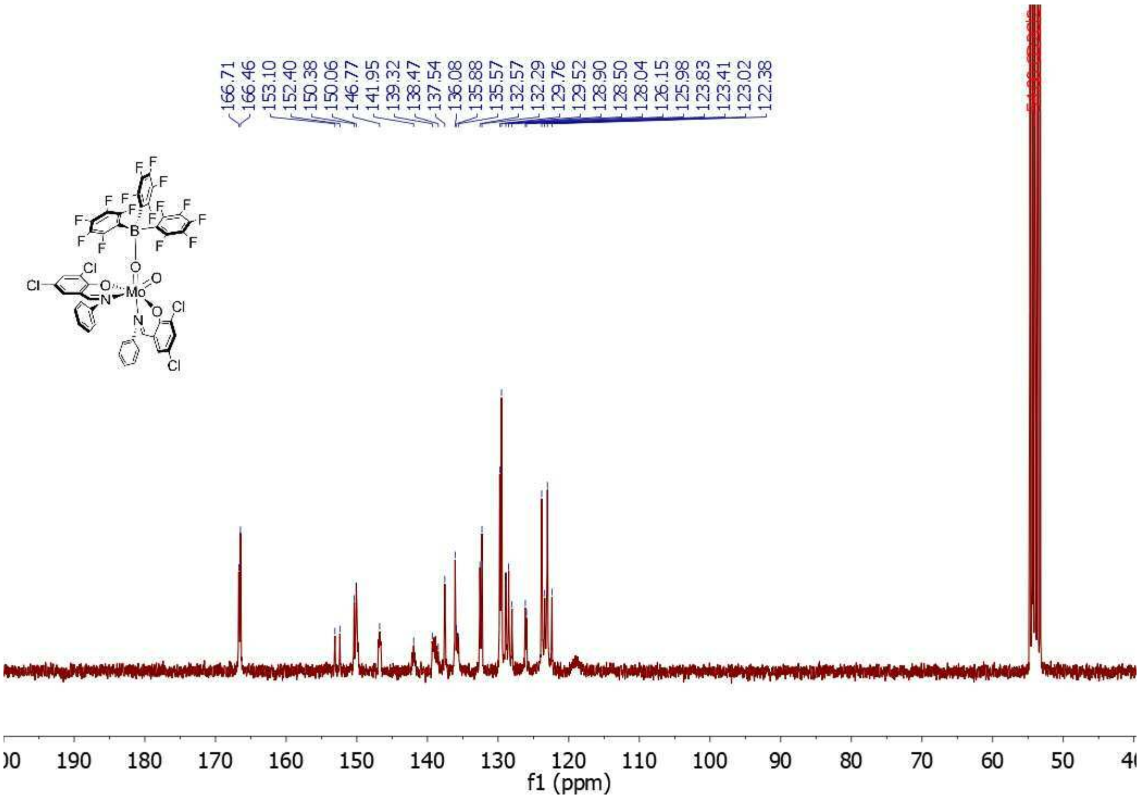

Figure S13. ^{13}C NMR spectra of complex **6** in CD_2Cl_2.

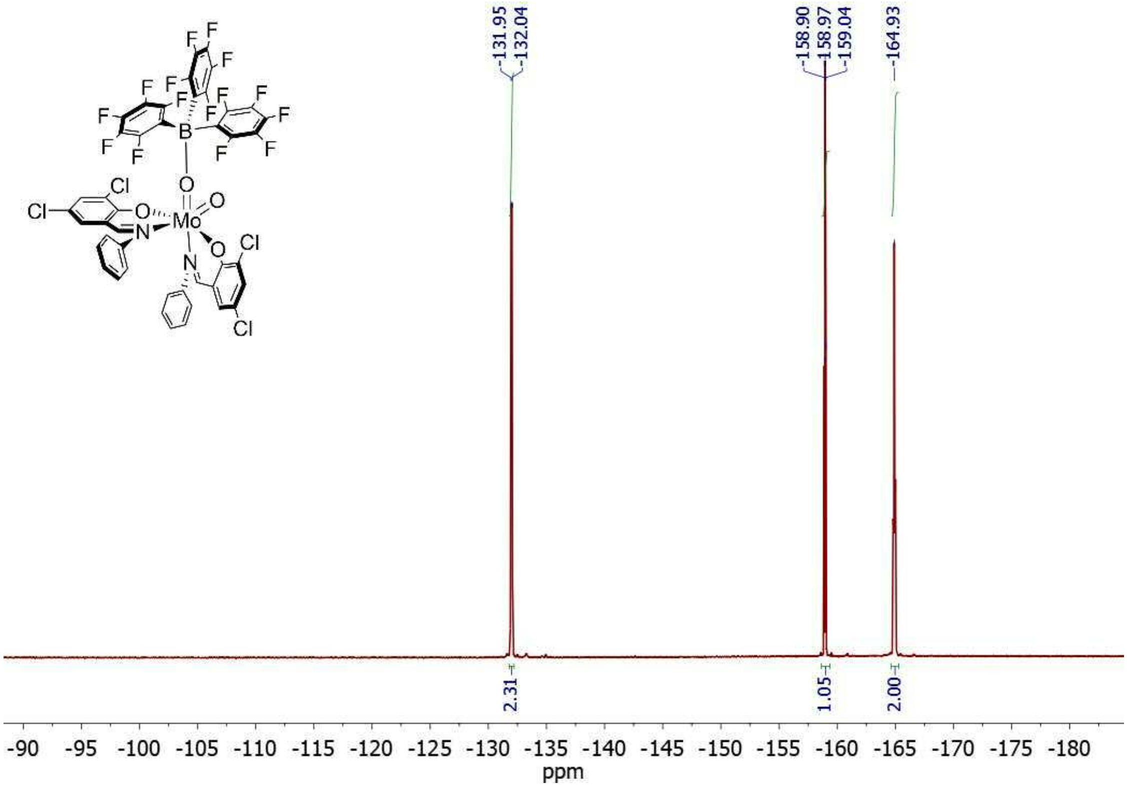

Figure S14. ^{19}F NMR spectra of complex **6** in CD_2Cl_2.

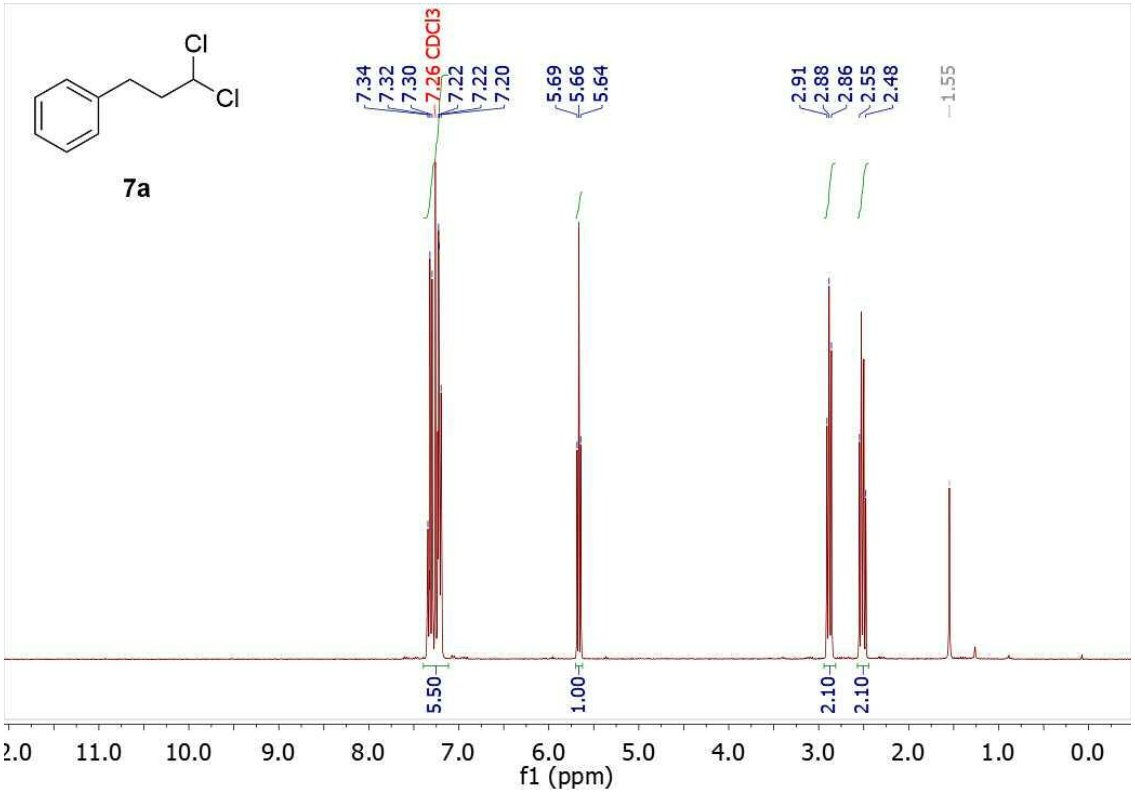

Figure S15. ^{1}H NMR spectra of **7a** in $CDCl_3$.

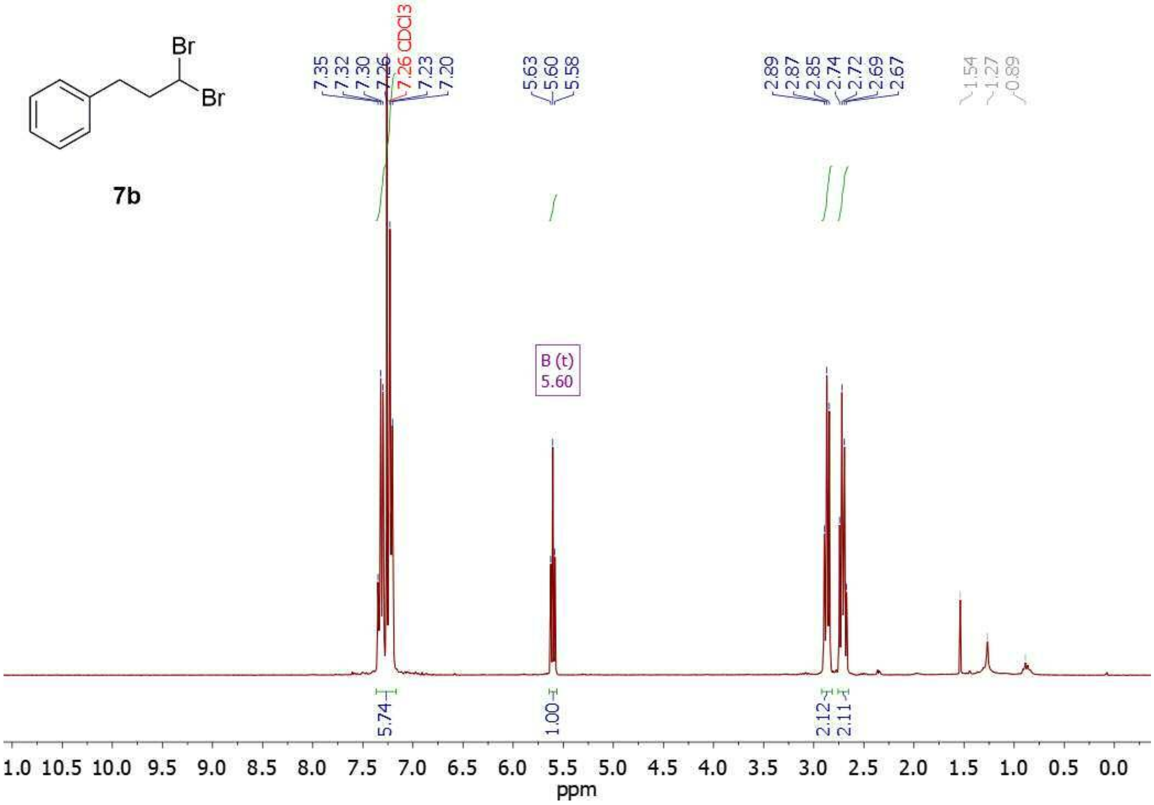

Figure S16. ^{1}H NMR spectra of **7b** in $CDCl_3$.

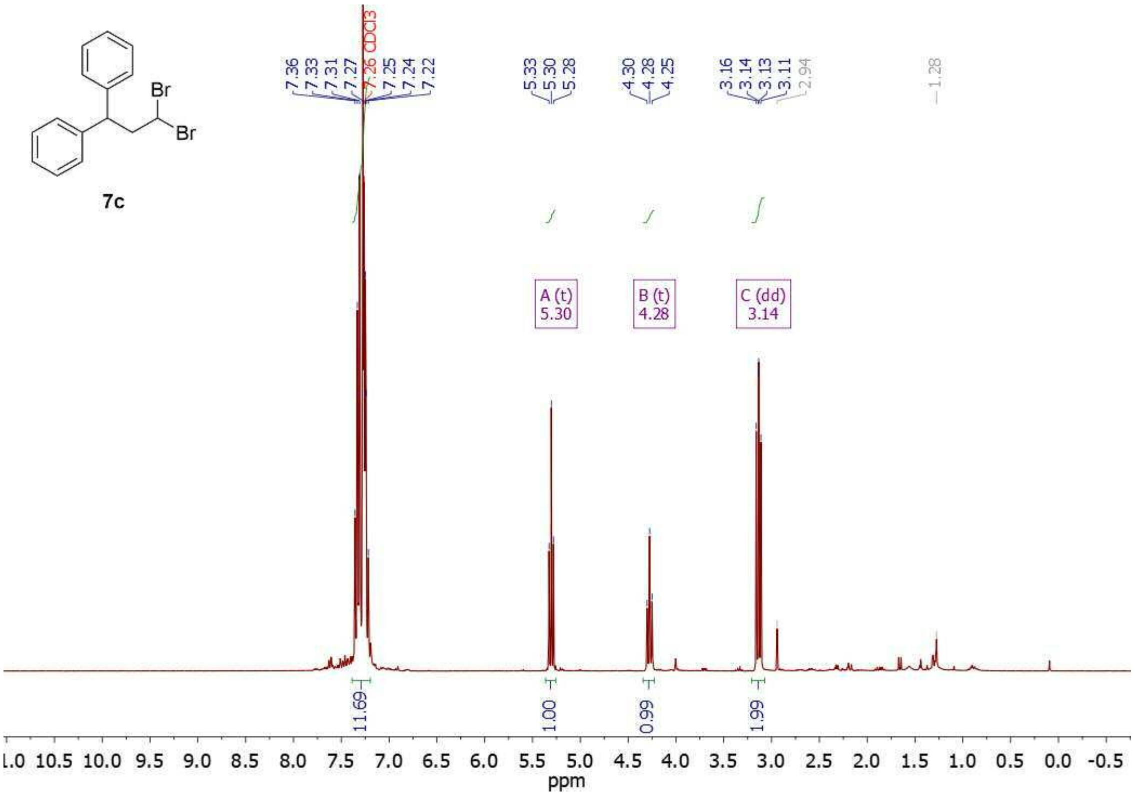

Figure S17. ^{1}H NMR spectra of **7c** in $CDCl_3$.

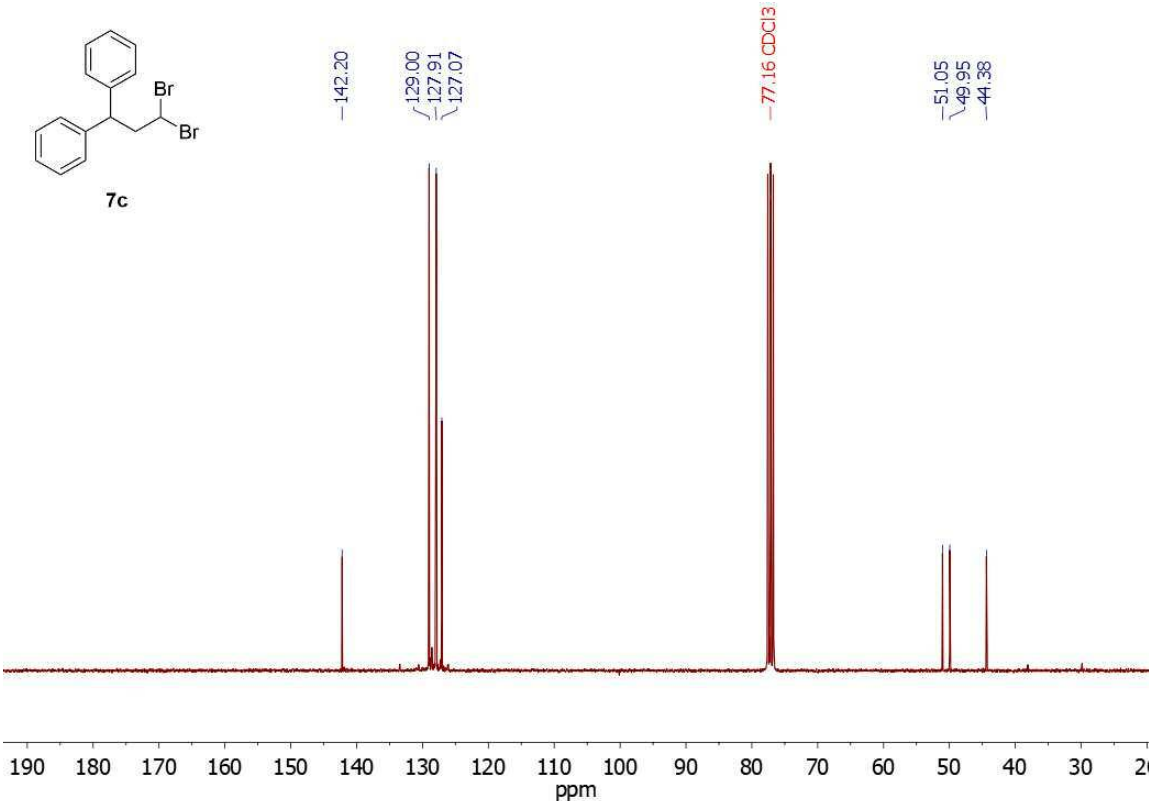

Figure S18. ^{13}C NMR spectra of **7c** in $CDCl_3$.

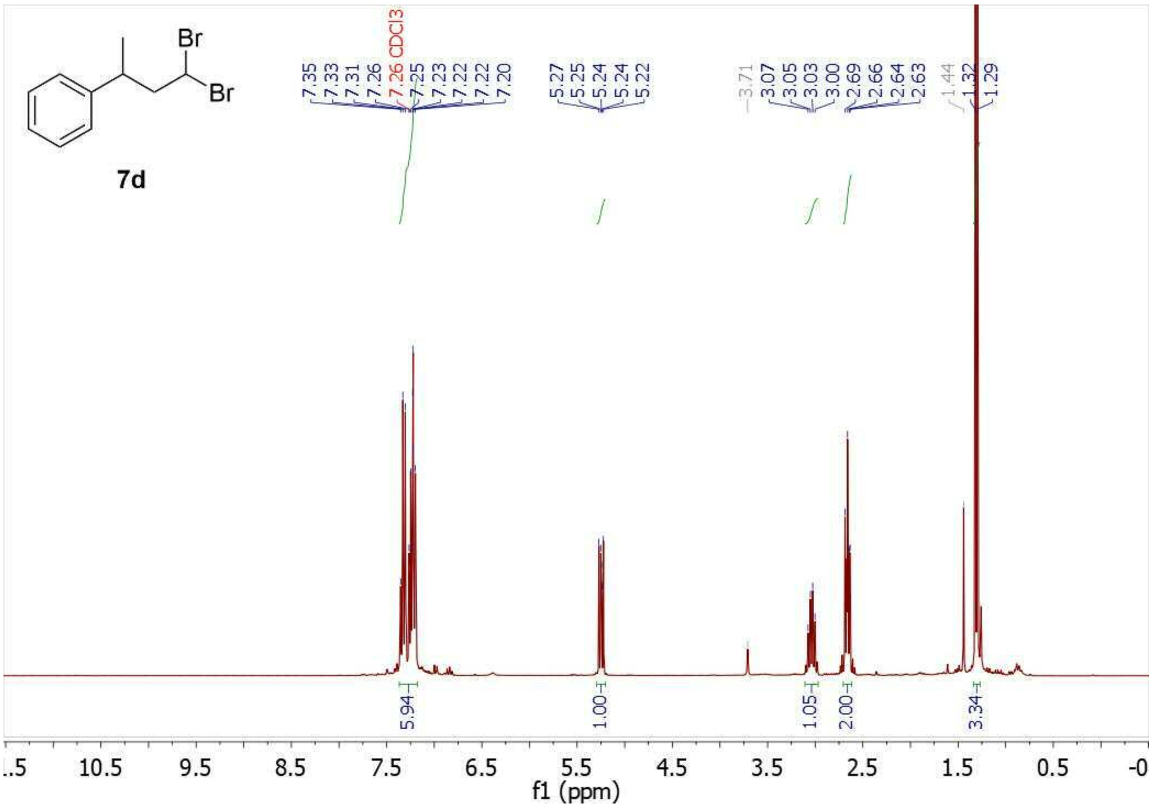

Figure S19. ^{1}H NMR spectra of **7d** in $CDCl_3$.

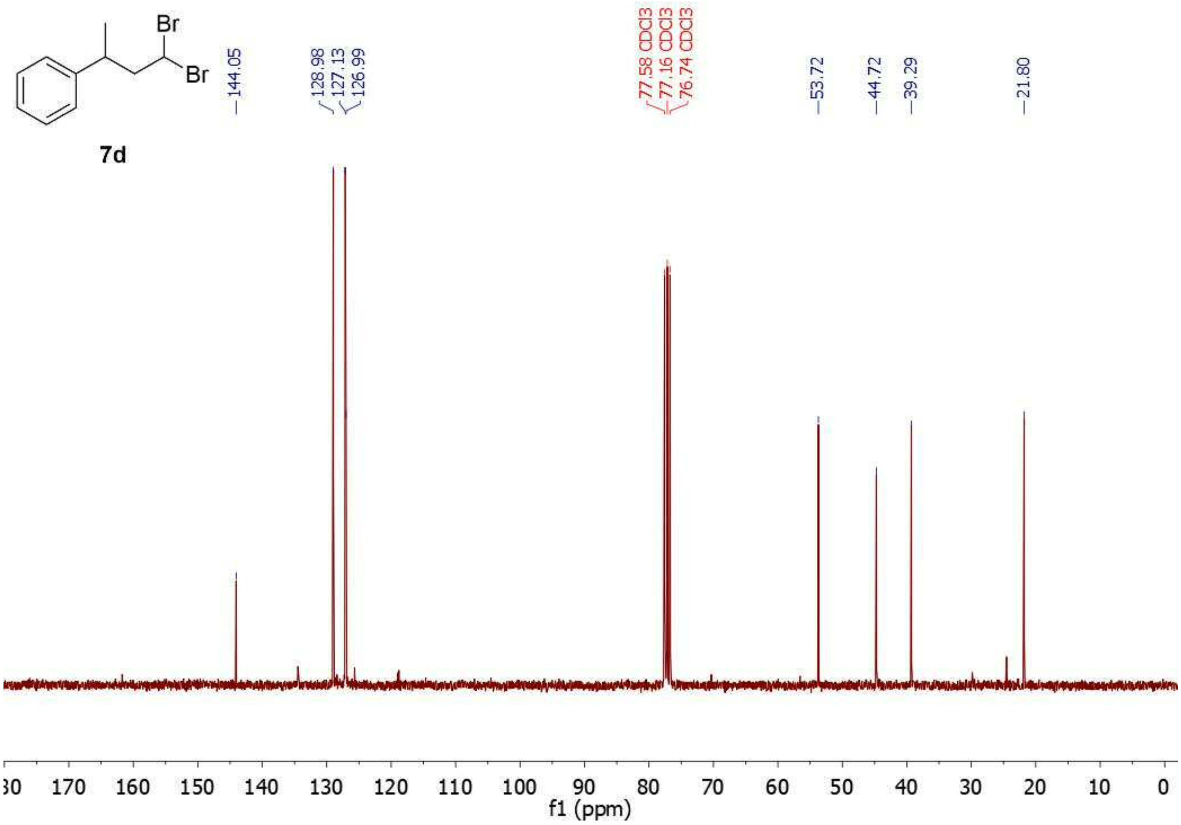

Figure S20. ^{13}C NMR spectra of **7d** in $CDCl_3$.

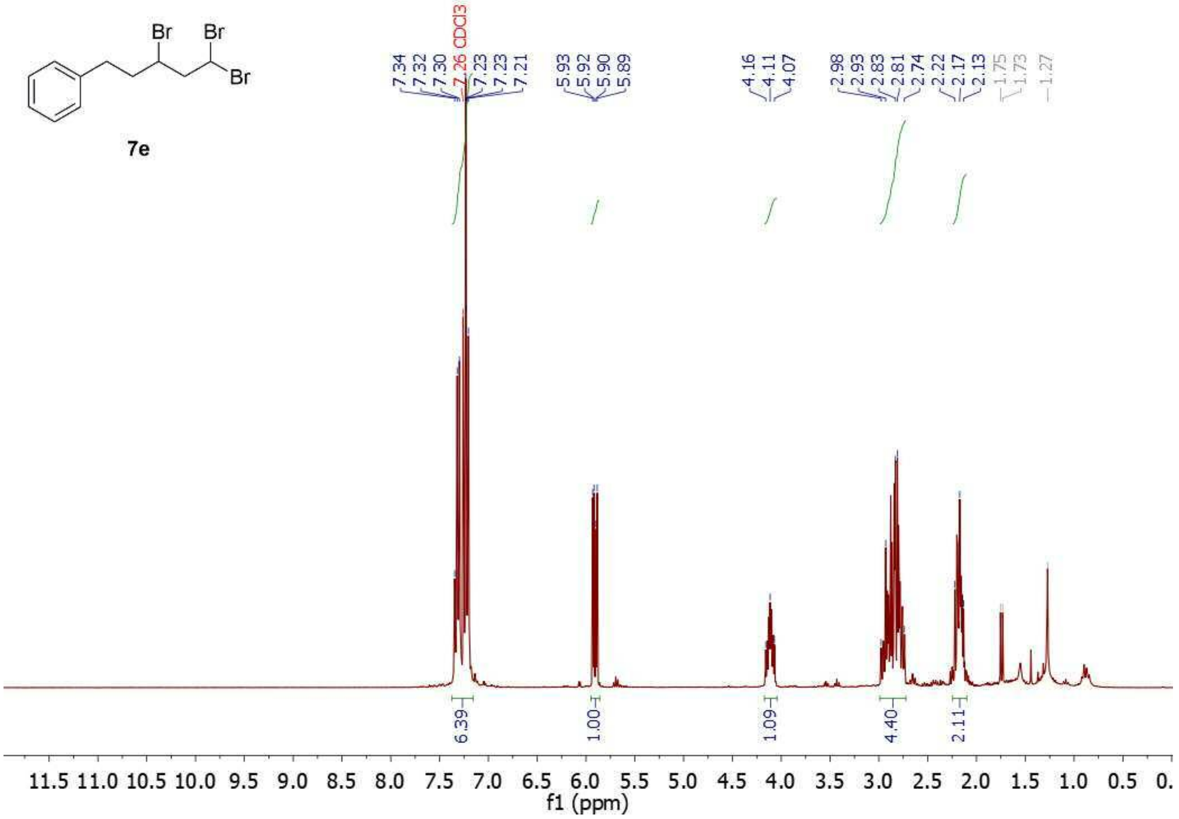

Figure S21. ^{1}H NMR spectra of **7e** in $CDCl_3$.

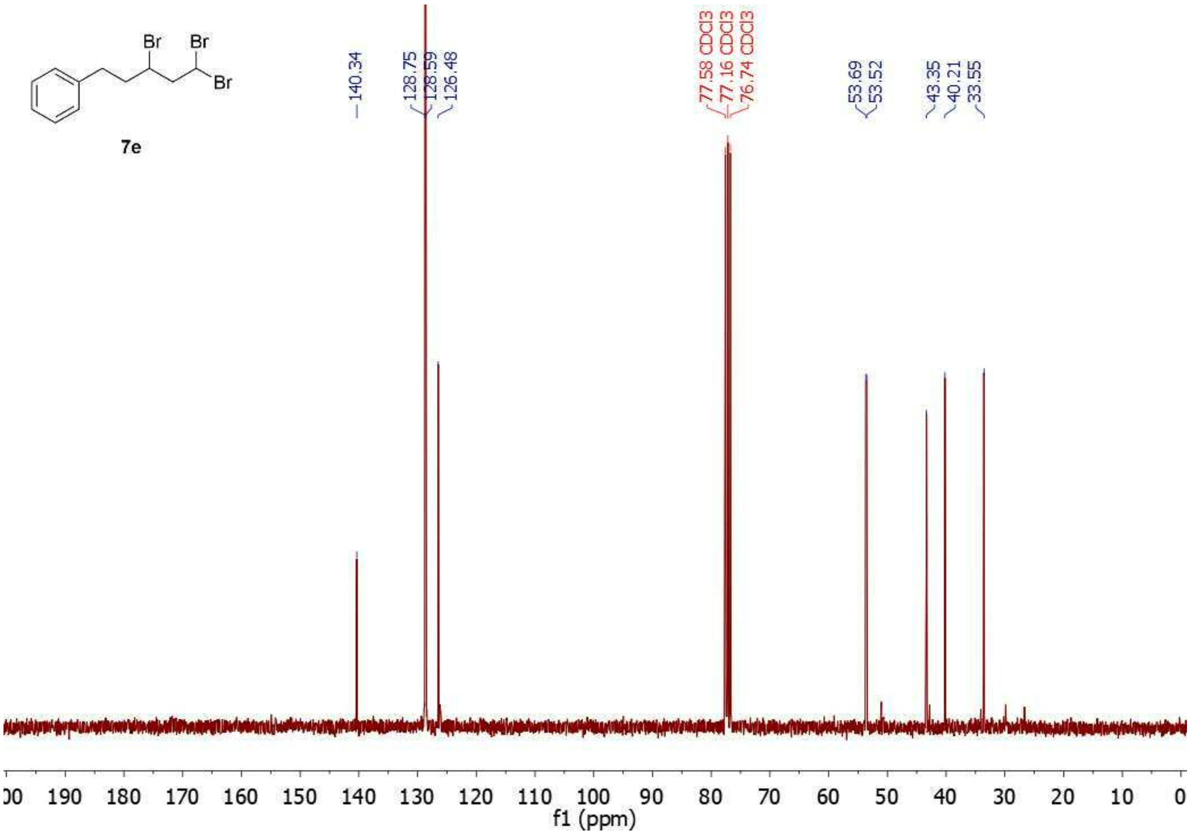

Figure S22. ^{13}C NMR spectra of **7e** in $CDCl_3$.

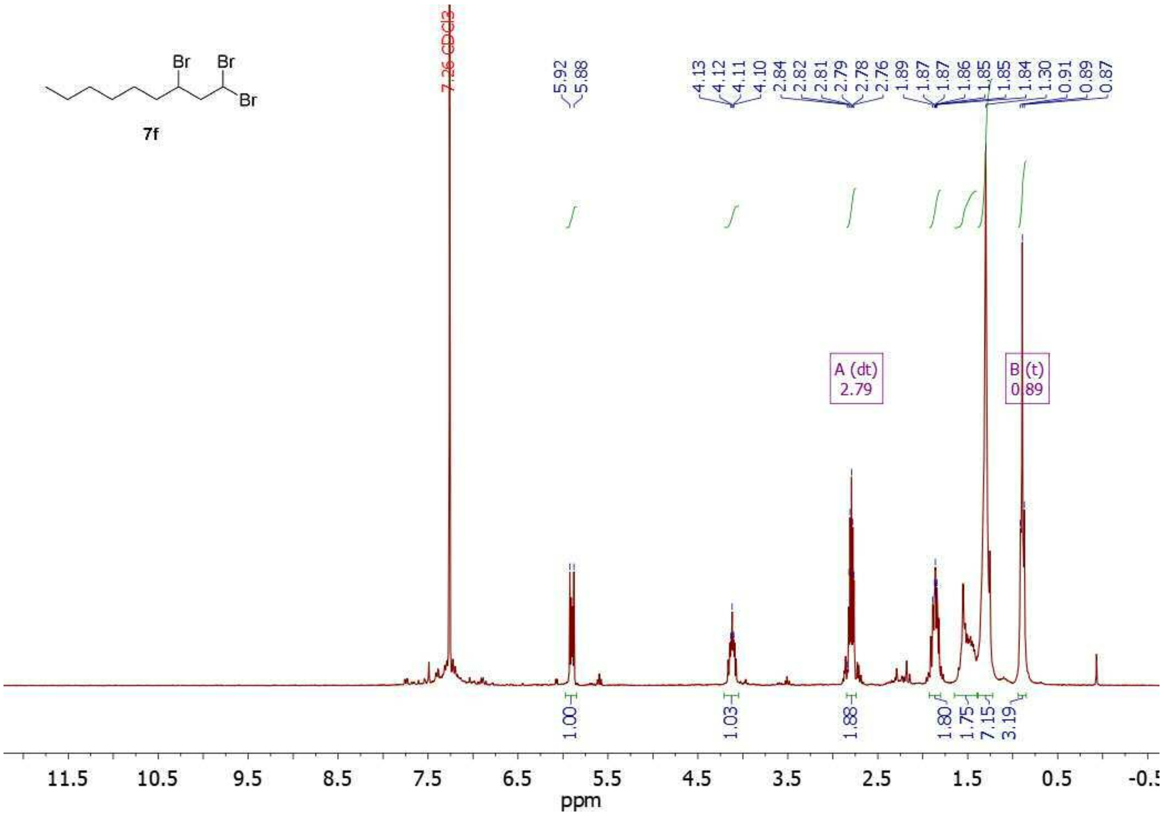

Figure S23. ^{1}H NMR spectra of **7f** in $CDCl_3$.

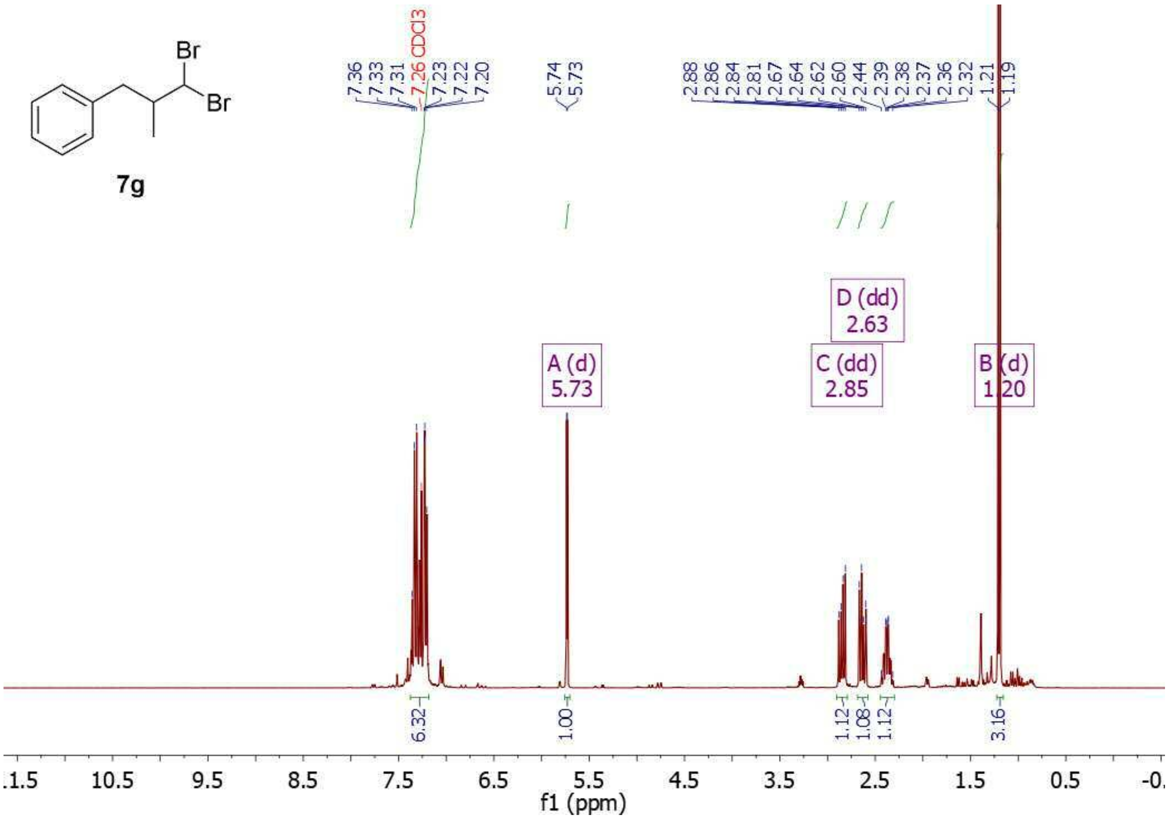

Figure S24. ^{1}H NMR spectra of **7g** in $CDCl_3$.

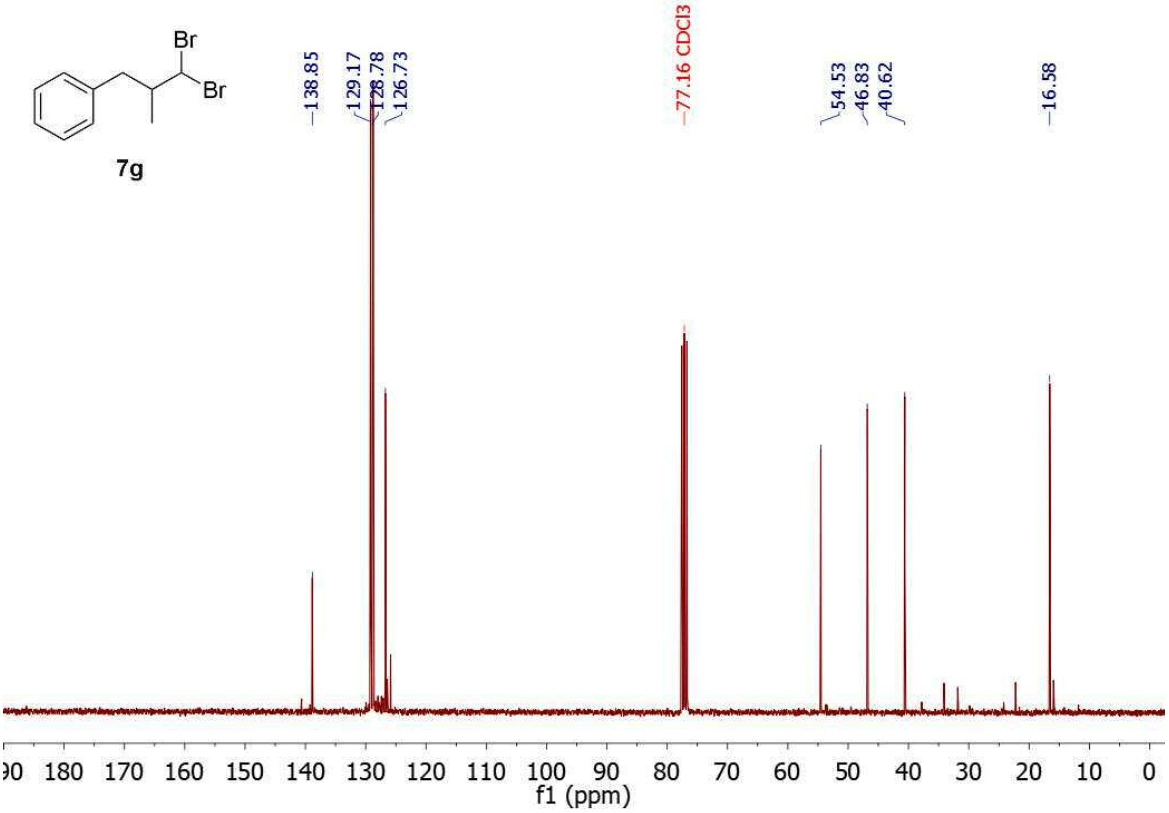

Figure S25. ^{13}C NMR spectra of **7g** in $CDCl_3$.

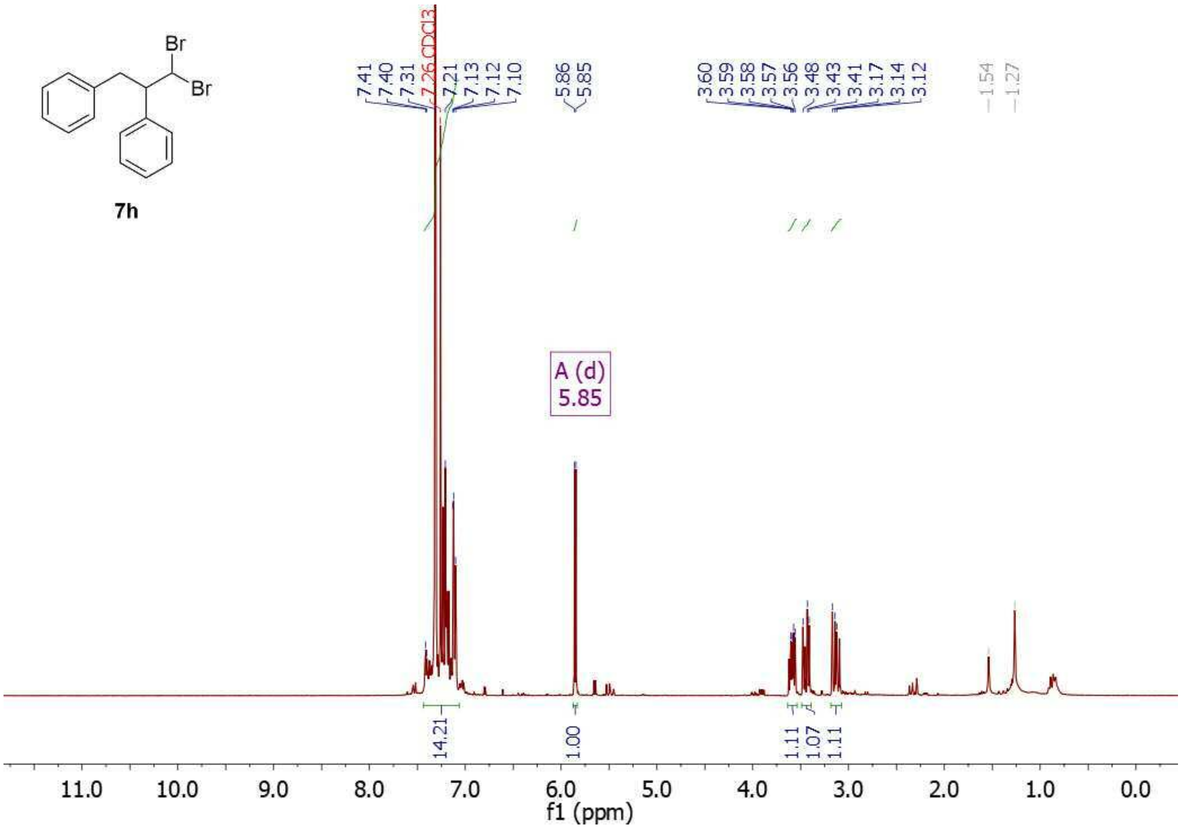

Figure S26. ^{1}H NMR spectra of **7h** in $CDCl_3$.

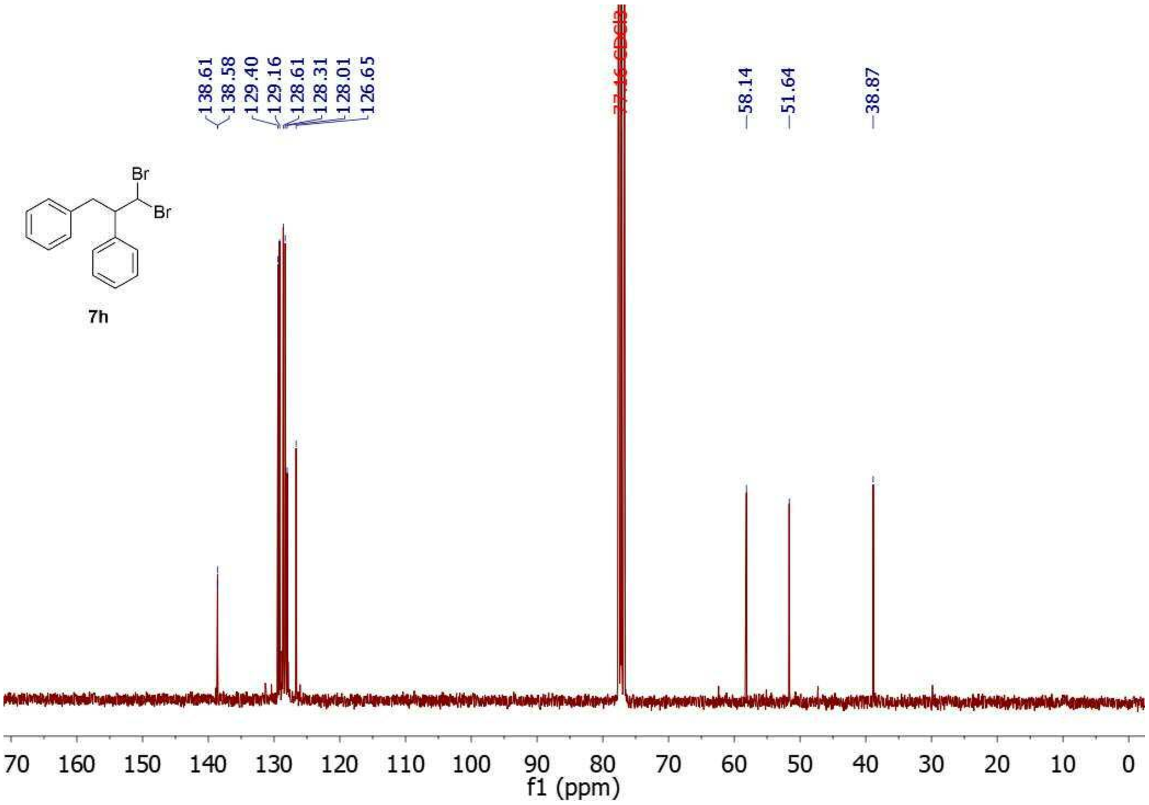

Figure S27. ^{13}C NMR spectra of **7h** in $CDCl_3$.

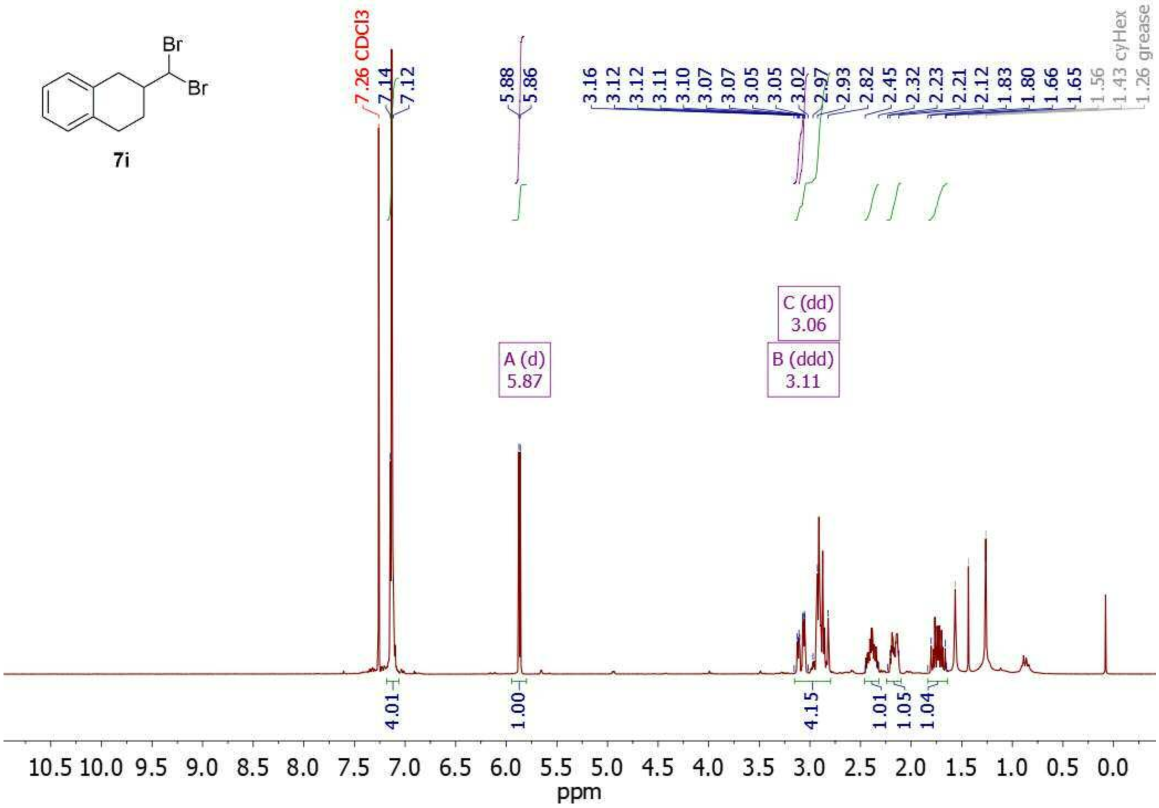

Figure S28. ^{1}H NMR spectra of **7i** in $CDCl_3$.

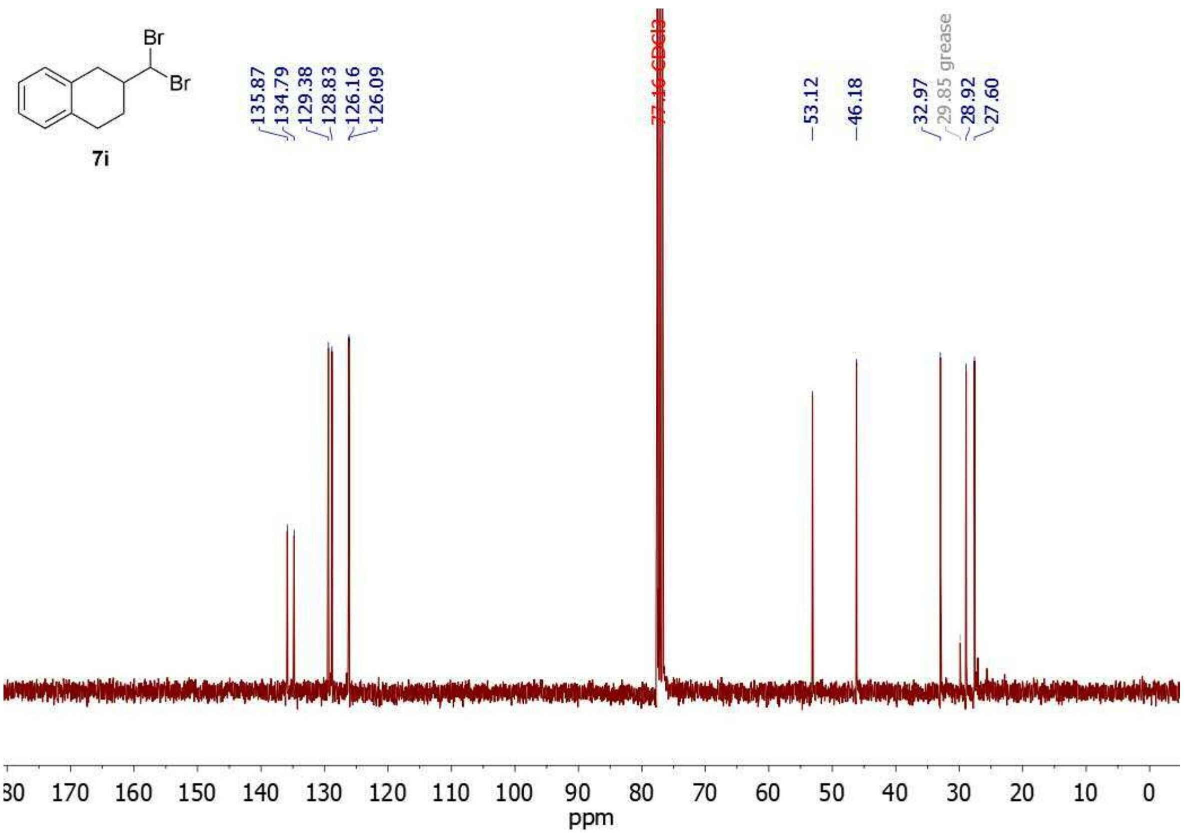

Figure S29. ^{13}C NMR spectra of **7i** in $CDCl_3$.

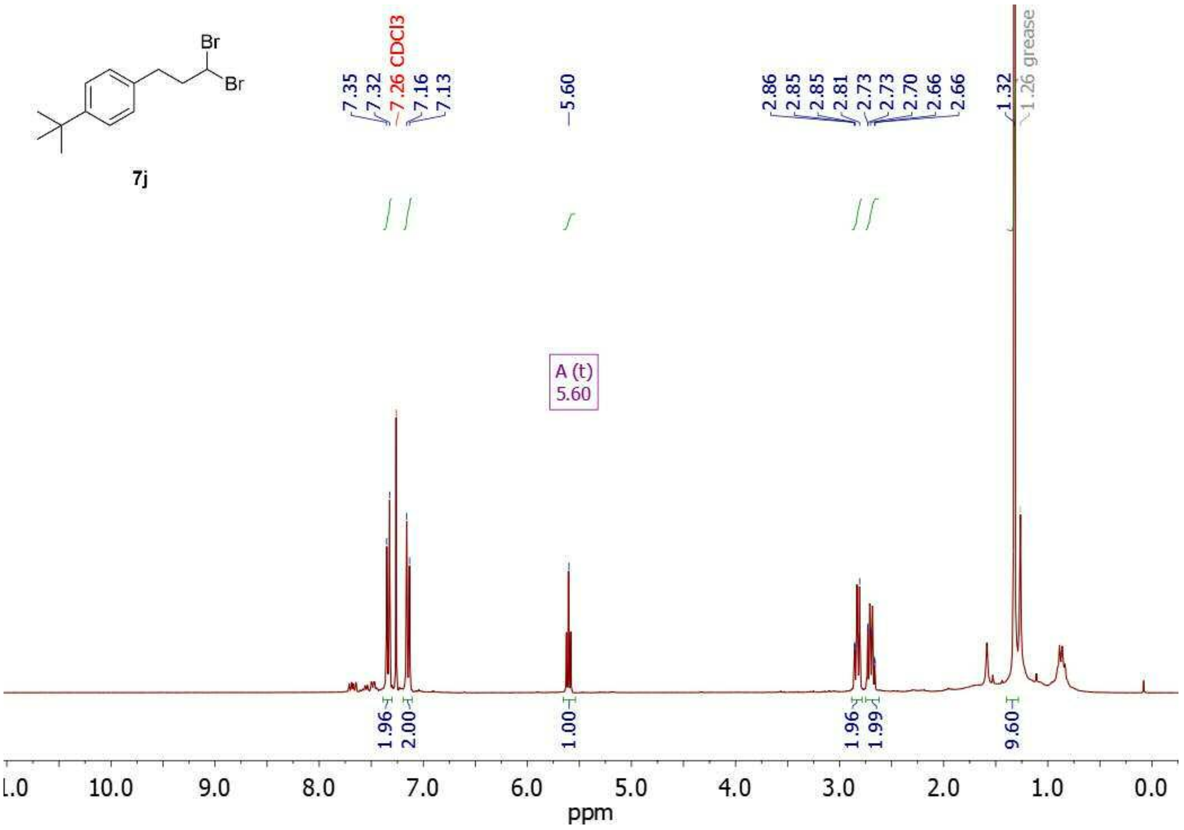

Figure S30. ^{1}H NMR spectra of **7j** in $CDCl_3$.

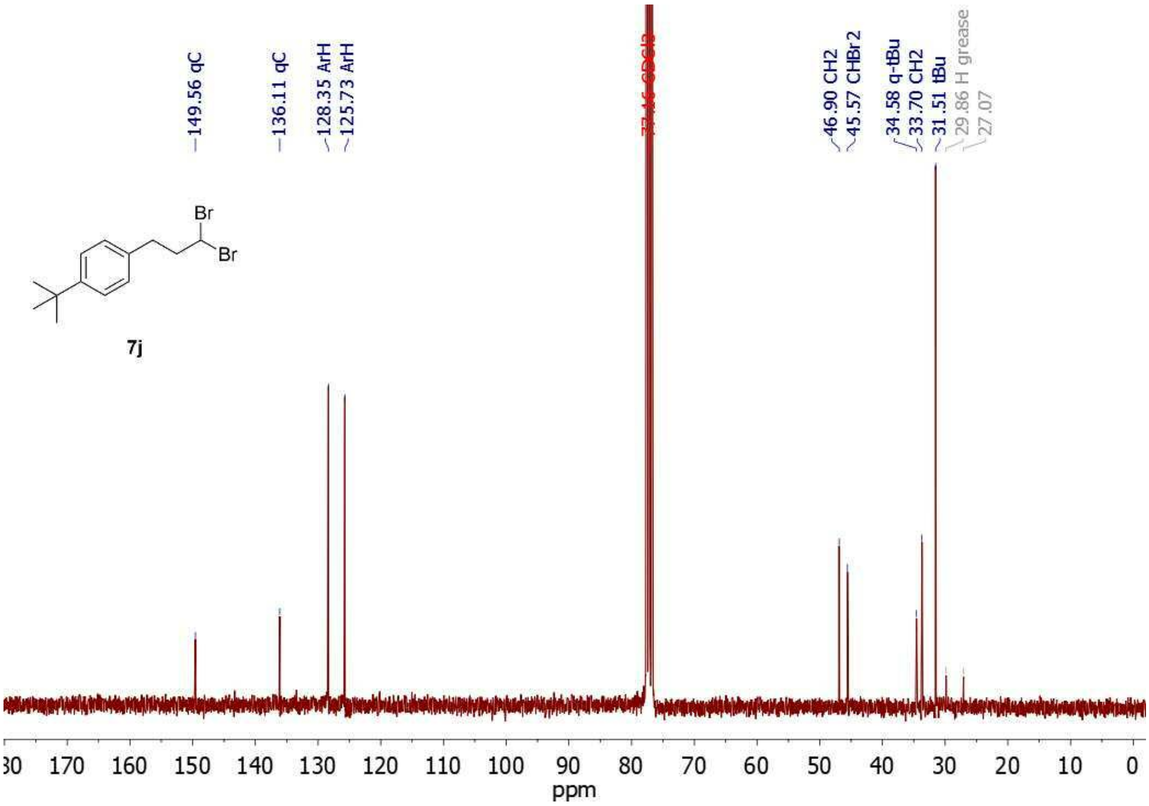

Figure S31. ^{13}C NMR spectra of **7j** in $CDCl_3$.

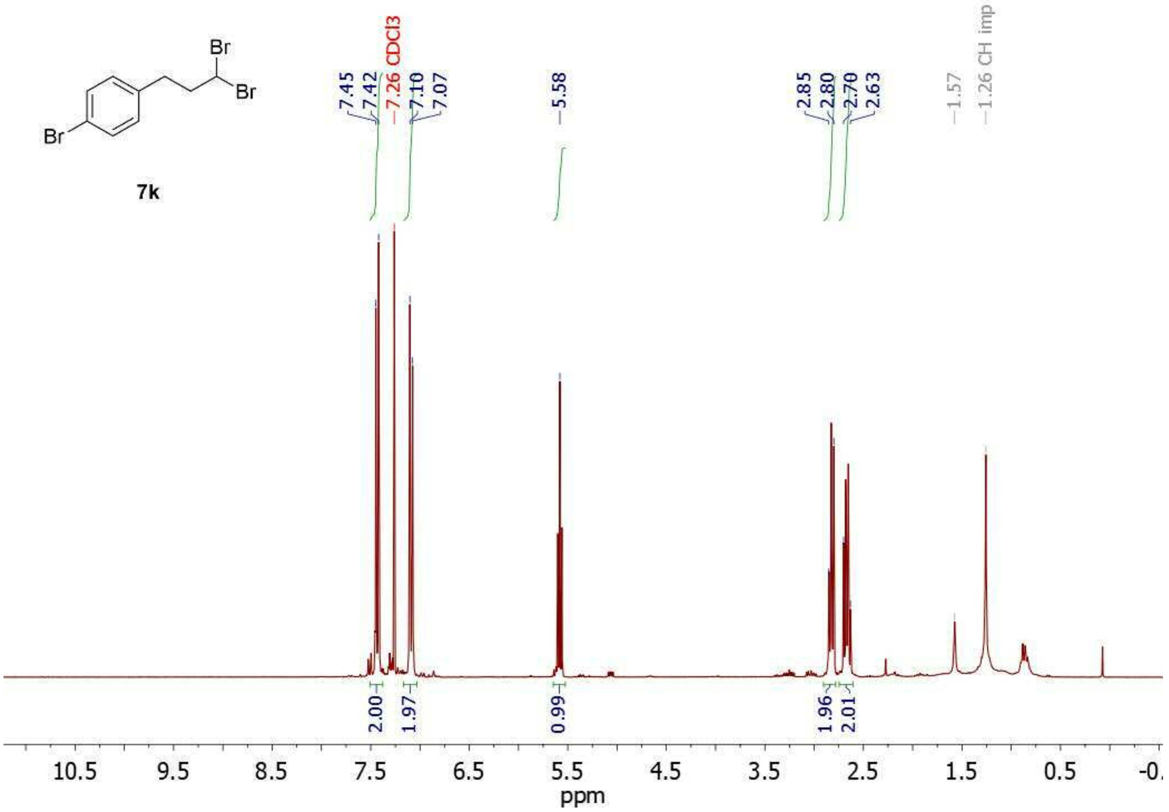

Figure S32. ^{1}H NMR spectra of **7k** in $CDCl_3$.

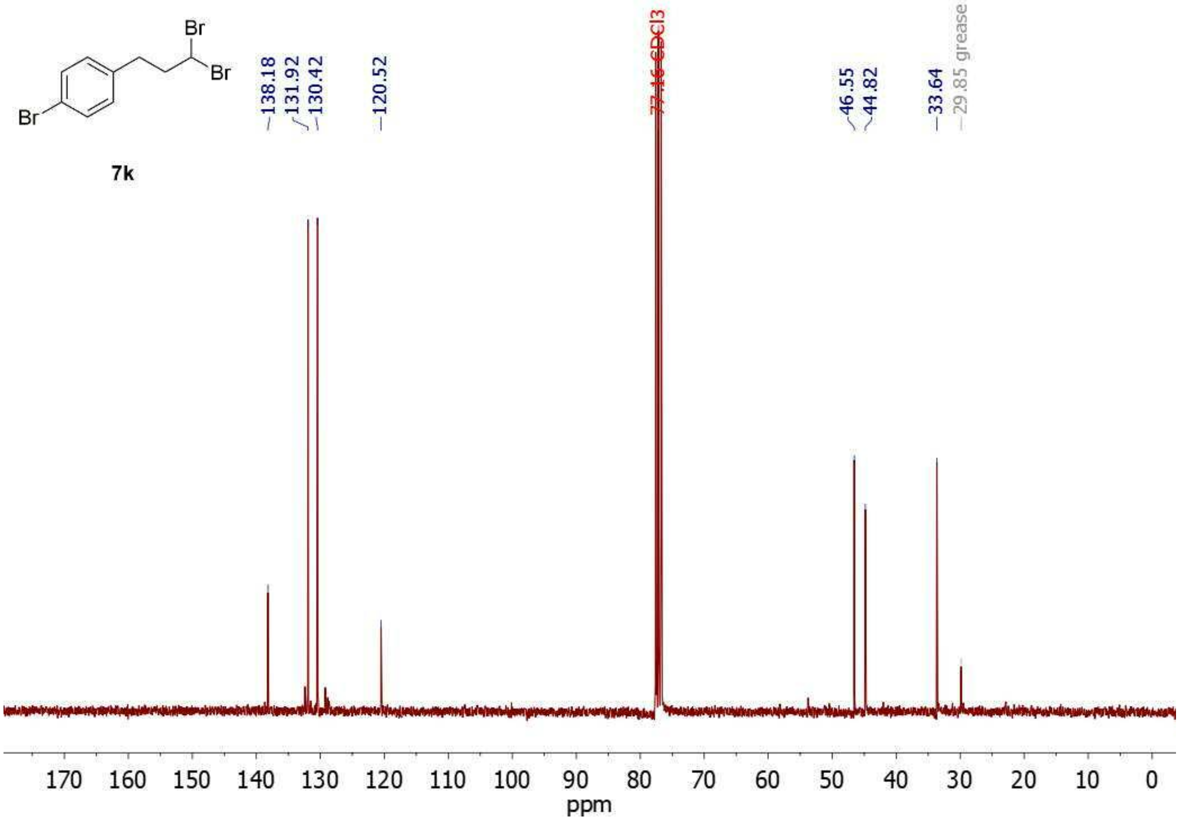

Figure S33. ^{13}C NMR spectra of **7k** in $CDCl_3$

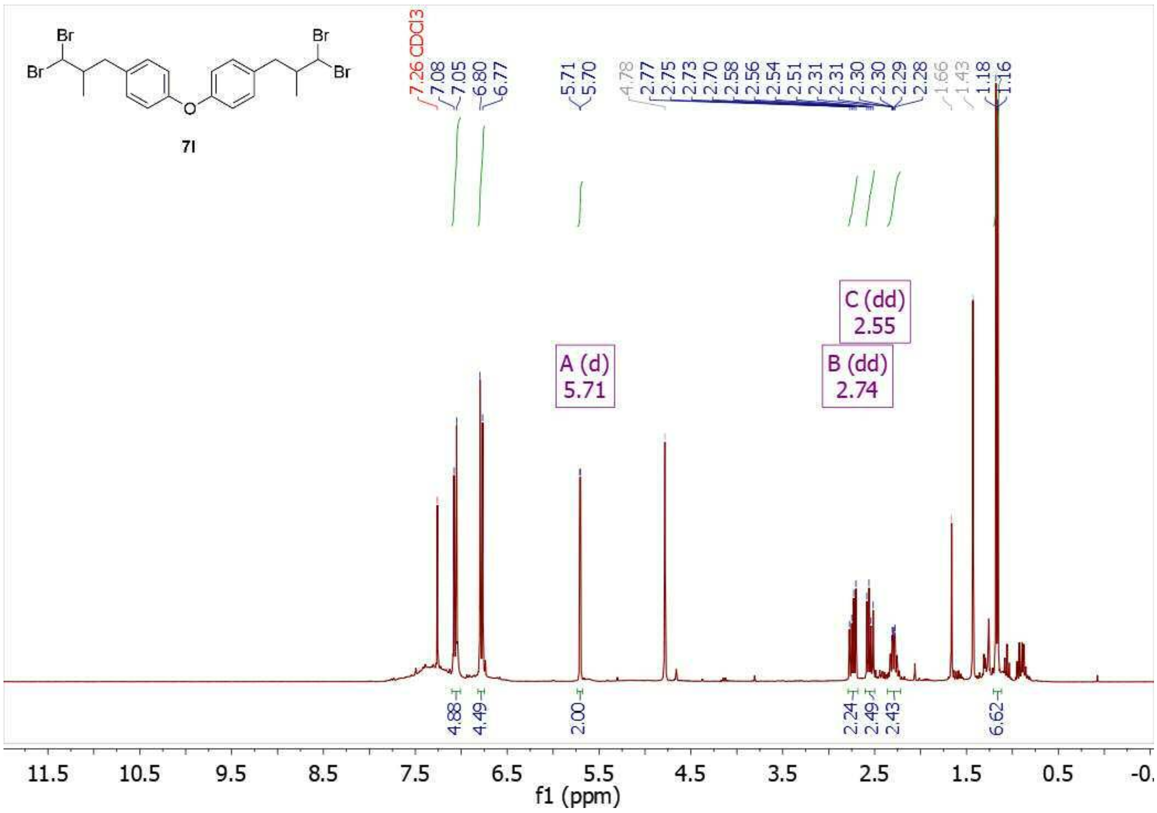

Figure S34. ^{1}H NMR spectra of **7l** in $CDCl_3$.

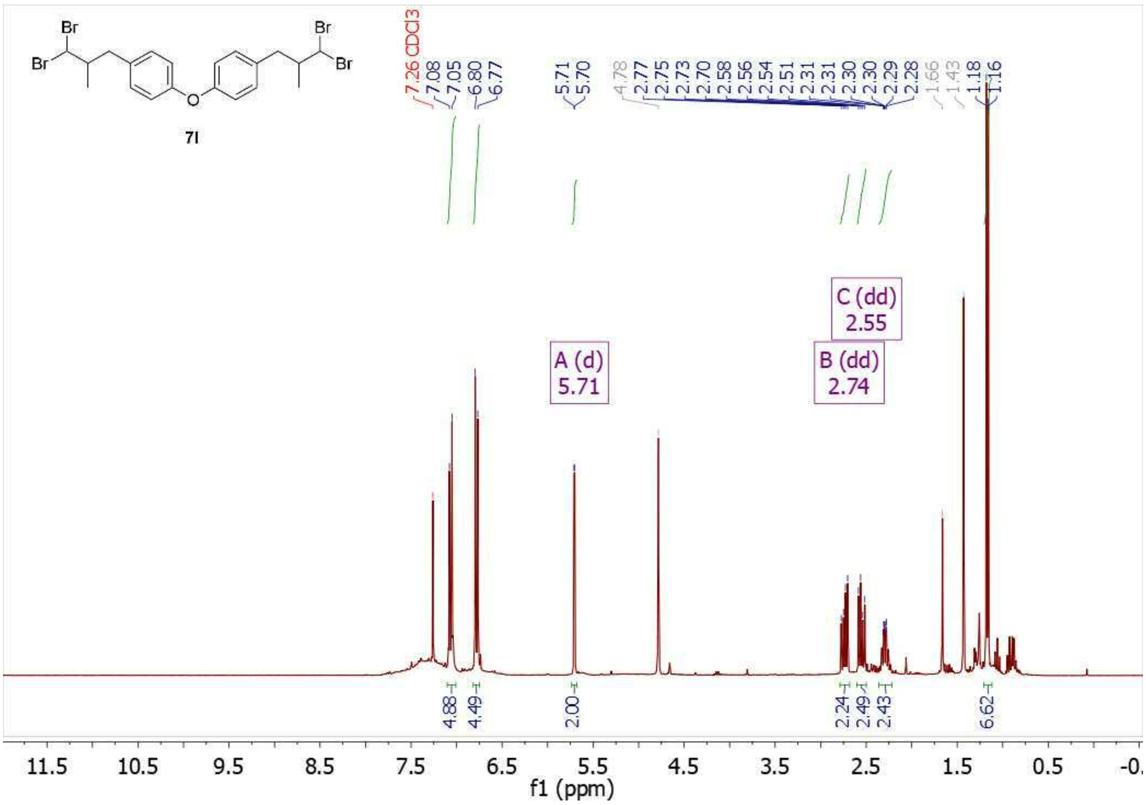

Figure S35. ^{13}C NMR spectra of **7l** in $CDCl_3$.

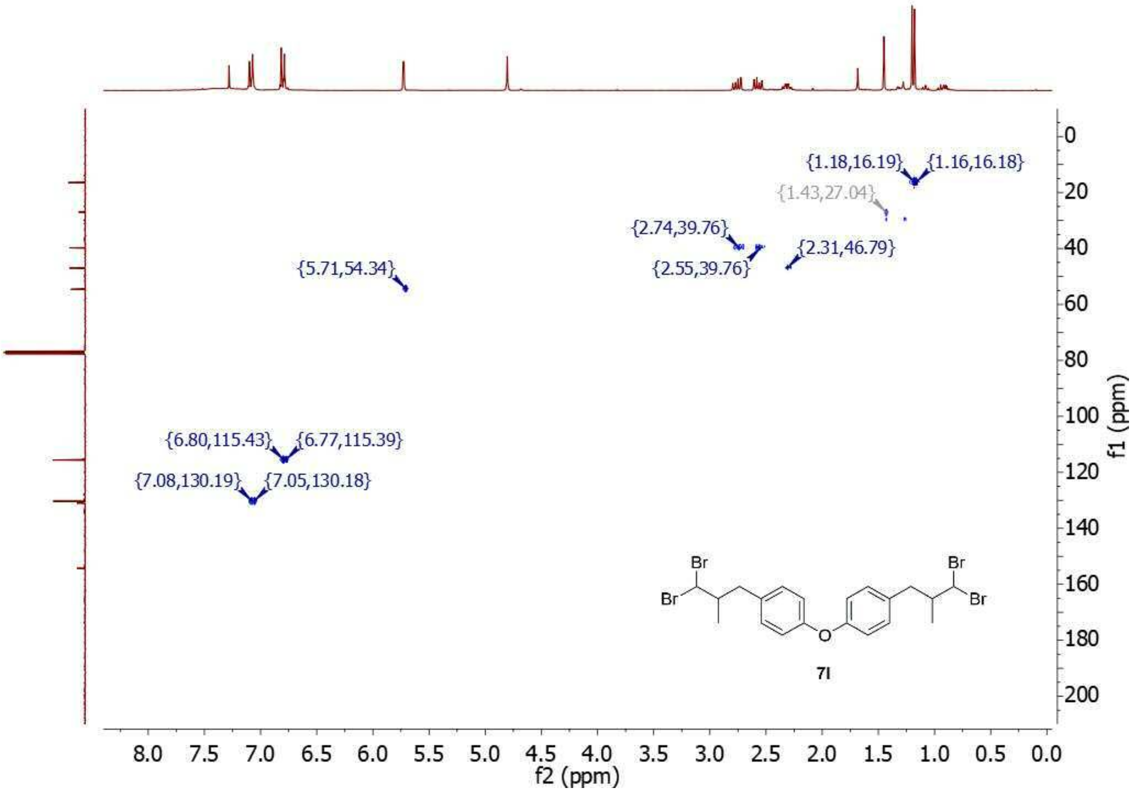

Figure S36. HSQC NMR spectra of **7l** in $CDCl_3$.

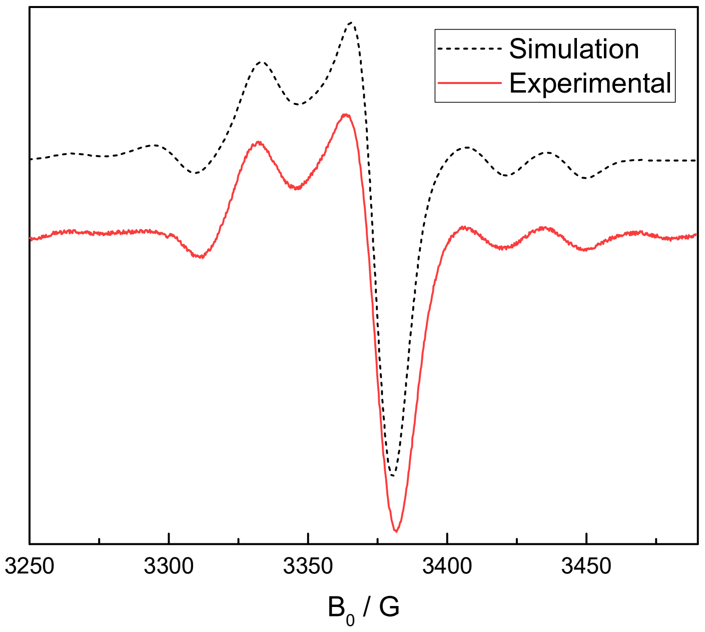

Figure S37. X-band EPR spectrum of C_6D_6 solution of the reaction of complex **4** with $PhSiH_3$ at 77K. Simulation parameters: axial g-tensor ($g_\perp$ = 2.0091, $g_\parallel$ = 2.0363), A_{Mo}(avg) = 27 G, natural abundance of ^{95}Mo (15.92%) and ^{97}Mo (9.55%).

X-band EPR spectrum of the reaction of complex **4** with $PhSiH_3$ in frozen solution exhibits axial g-tensor ($g_x = g_y = g_\perp$ = 2.0091, $g_z = g_\parallel$ = 2.0363), with typical for Mo(V) complexes g-values. This points on an axial symmetry of Mo complex. Hyperfine couplings of coordinated nitrogen atoms are not resolved due to the substantially large line width.

Crystallographic data for complexes 1-6

The X-ray data collections were performed with a Bruker AXS SMART APEX-II CCD diffractometer at 100 K with Mo-K_α radiation (λ = 0.71073 Å) from an Incoatec microfocus sealed tube equipped with a multilayer monochromator. Absorption corrections were made semi-empirically from equivalents. The structures were solved by direct methods (SHELXS-97)[1] and refined by full-matrix least-squares techniques against F^2 (SHELXL-2014/6).[2] A weighting scheme of $w = 1/[\sigma^2(F_o^2)+(aP)^2+bP]$ where $P = (F_o^2+2F_c^2)/3$ was used. The absolute configuration of **3** was established by anomalous dispersion effects in the diffraction measurements of the crystal. The non-hydrogen atoms of **1**, **3**, **4**, and **6** were refined with anisotropic displacement parameters without any constraints. The H atoms of the phenyl rings including any adjacent CH=N groups were put at the external bisectors of the C–C–C angles at C–H distances of 0.95 Å and common isotropic displacement parameters were refined for the H atoms of the same ring. The H atoms of the *tert*-butyl groups were refined with common isotropic displacement parameters for the H atoms of the same group and idealized geometries with tetrahedral angles, enabling rotations around the C–C bonds, and C–H distances of 0.98 Å. Crystallographic data for the structures of compounds **1-6** have been deposited with the Cambridge Crystallographic Data Center (CCDC 1940999 to CCDC 1941004 for **1** to **6**).

Crystal structure determination of 2. The trifluoromethyl group bonded to C203 and the *tert*-butyl group bonded to C14 were disordered over two orientations and refined with common site occupation factors of 0.766(2) and 0.234(2), respectively. In these disordered groups the C–C bonds were restrained to 1.53 Å and the same anisotropic displacement parameters were used for equivalent atoms. The acetonitrile solvent molecule disordered around an inversion center (N6) was refined as a rigid body and the equivalent bonds were restrained to have the same lengths as the solvent molecule N3. The other non-hydrogen atoms were refined with anisotropic displacement parameters without any constraints.

Crystal structure determination of 5. The pentane solvate molecule and the nearby situated trifluoromethyl group bonded to C103 and the *tert*-butyl group bonded to C24 were disordered over two orientations and refined with common site occupation factors of 0.665(3) and 0.335(3), respectively. In these disordered regions, the C–C bonds were restrained to 1.53 Å and the same anisotropic displacement parameters were used for equivalent atoms; the solvent molecule was refined as a rigid body. The other non-hydrogen atoms were refined with anisotropic displacement parameters without any constraints. The H atoms of the solvent molecule were included at calculated positions with their isotropic displacement parameters fixed to 1.2 times U_{eq} of the C atom they are bonded to.

Table S1. Crystal data and structure refinement details for **1-3**.

**Crystal data**	**1**	**2**	**3**
CIF data code	NZ398I2	NZ416	SW336
Empirical formula	$C_{42}H_{52}MoN_2O_4$	$2(C_{46}H_{48}F_{12}MoN_2O_4) \cdot 7(C_2H_3N)$	$C_{26}H_{16}Cl_4MoN_2O_4$
Formula weight	744.79	2320.98	658.15
Crystal description	block, yellow	needle, yellow	needle, yellow
Crystal size	0.32 x 0.15 x 0.11mm	0.32 x 0.15 x 0.15mm	0.23 x 0.22 x 0.17mm
Temperature	100K	100K	100K
Crystal system	monoclinic	triclinic	monoclinic
Space group	P 2_1/n	P -1	C c
Unit cell dimensions: a	11.4166(5)Å	10.9604(14)Å	15.9025(19)Å
b	22.1197(9)Å	13.0459(16)Å	14.2707(16)Å
c	15.2646(5)Å	20.527(3)Å	13.0192(15)Å
α		97.266(4)°	
β	92.020(2)°	97.247(4)°	122.466(2)°
γ		105.745(3)°	
Volume	3852.4(3)Å^3	2762.4(6)Å^3	2492.8(5)Å^3
Z	4	1	4
Calc. density	1.284Mg/m^3	1.395Mg/m^3	1.754Mg/m^3
F(000)	1568	1194	1312
Linear absorption coefficient μ	0.383mm^{-1}	0.326mm^{-1}	0.993mm^{-1}
Max. and min. transmission	1.000 and 0.783	1.000 and 0.693	1.000 and 0.757
Unit cell determination	2.56° < Θ < 27.62°	2.35° < Θ < 26.03°	2.18° < Θ < 30.36°
Reflections used	8693	9946	5708
**Data collection**			
Θ range for data collection	2.19 to 27.00°	1.64 to 26.00°	2.08 to 30.00°
Reflections collected/ unique	29139 / 8407	64974 / 10855	22403 / 6852
Significant unique reflections	6839 with I > 2σ(I)	8892 with I > 2σ(I)	5999 with I > 2σ(I)
R(int), R(sigma)	0.0431, 0.0462	0.0757, 0.0592	0.0708, 0.0710
Completeness to Θ_{max}	99.9%	99.9%	99.9%
**Refinement**			
Data/ parameters/ restraints	8407 / 470 / 0	10855 / 732 / 11	6852 / 338 / 2
Goodness-of-fit on F^2	1.018	1.060	1.031
Final R indices [I > 2σ(I)]	R1 = 0.0315, wR2 = 0.0655	R1 = 0.0414, wR2 = 0.1026	R1 = 0.0395, wR2 = 0.0776
R indices (all data)	R1 = 0.0462, wR2 = 0.0703	R1 = 0.0568, wR2 = 0.1109	R1 = 0.0521, wR2 = 0.0836
Weighting scheme param. a, b	0.0217, 2.7255	0.0564, 0.9228	0.0319, 0.2614
Largest Δ/σ in last cycle	0.003	0.002	0.001
Largest diff. peak and hole	0.385 and -0.643e/Å^3	0.801 and -0.980e/Å^3	0.975 and -0.867e/Å^3
**CCDC no.**	1940999	1941000	1941001

Table S2. Crystal data and structure refinement details for **4-6**.

**Crystal data**	**4**	**5**	**6**
CIF data code	NZ469	NZ417	NZSK14
Empirical formula	$C_{60}H_{52}BF_{15}MoN_2O_4$	$C_{64}H_{48}BF_{27}MoN_2O_4 \cdot C_5H_{12}$	$C_{44}H_{16}BCl_4F_{15}MoN_2O_4$
Formula weight	1256.78	1600.94	1170.14
Crystal description	block, red	block, red	block, red
Crystal size	0.33 x 0.28 x 0.14mm	0.34 x 0.30 x 0.27mm	0.26 x 0.23 x 0.14mm
Temperature	100K	100K	100K
Crystal system	monoclinic	monoclinic	triclinic
Space group	P 2_1/n	P 2_1/c	P -1
Unit cell dimensions: a	20.754(3)Å	13.5961(9)Å	11.9661(8)Å
b	11.0642(14)Å	19.2162(13)Å	12.4441(8)Å
c	26.237(3)Å	26.6881(17)Å	16.2689(10)Å
α			96.669(3)°
β	110.783(6)°	94.291(3)°	95.553(3)°
γ			114.585(4)°
Volume	5632.7(12)Å^3	6953.1(8)Å^3	2159.6(2)Å^3
Z	4	4	2
Calc. density	1.482Mg/m^3	1.529Mg/m^3	1.799Mg/m^3
F(000)	2560	3240	1152
Linear absorption coefficient μ	0.332mm^{-1}	0.310mm^{-1}	0.665mm^{-1}
Max. and min. transmission	1.000 and 0.837	1.000 and 0.747	1.000 and 0.825
Unit cell determination	2.45° < Θ < 30.73°	2.46° < Θ < 26.56°	2.35° < Θ < 35.80°
Reflections used	9660	9950	9960
**Data collection**			
Θ range for data collection	1.66 to 30.00°	1.50 to 26.00°	1.28 to 35.00°
Reflections collected/ unique	62872 / 16421	70117 / 13651	55444 / 19018
Significant unique reflections	13646 with I > 2σ(I)	10417 with I > 2σ(I)	16076 with I > 2σ(I)
R(int), R(sigma)	0.0321, 0.0316	0.0384, 0.0552	0.0404, 0.0409
Completeness to Θ_{max}	100.0%	99.9%	100.0%
**Refinement**			
Data/ parameters/ restraints	16421 / 768 / 0	13651 / 958 / 16	19018 / 644 / 0
Goodness-of-fit on F^2	1.026	1.024	1.031
Final R indices [I > 2σ(I)]	R1 = 0.0317, wR2 = 0.0753	R1 = 0.0532, wR2 = 0.1425	R1 = 0.0298, wR2 = 0.0708
R indices (all data)	R1 = 0.0429, wR2 = 0.0807	R1 = 0.0724, wR2 = 0.1571	R1 = 0.0408, wR2 = 0.0771
Weighting scheme param. a, b	0.0335, 3.5282	0.0940, 4.1449	0.0254, 1.2819
Largest Δ/σ in last cycle	0.001	0.002	0.005
Largest diff. peak and hole	0.635 and -0.521e/Å^3	1.034 and -1.064e/Å^3	0.742 and -0.560e/Å^3
**CCDC no.**	1941002	1941003	1941004

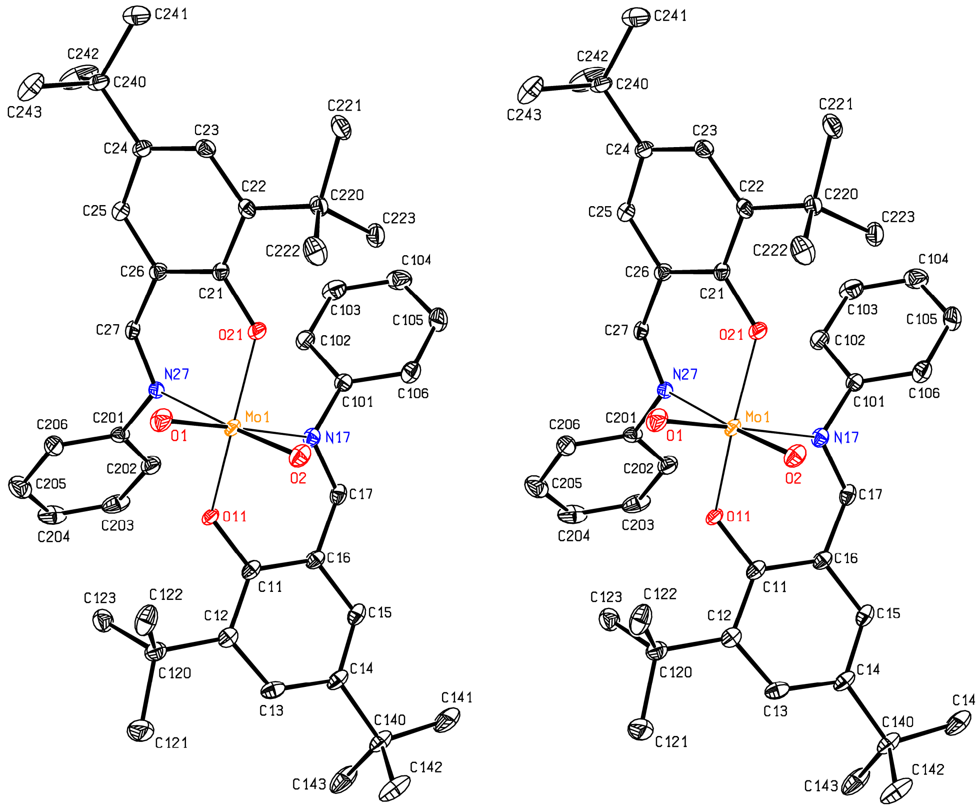

Figure S38. Stereoscopic ORTEP[3] plot of **1** showing the atomic numbering scheme. The probability ellipsoids are drawn at the 50% probability level. The H atoms were omitted for clarity.

Table S3. Selected bond lengths [Å] and angles [°] for **1**.

Mo1-O1	1.6983(15)
Mo1-O2	1.6984(13)
Mo1-O11	1.9608(13)
Mo1-O21	1.9409(13)
Mo1-N17	2.3896(16)
Mo1-N27	2.3898(15)
O11-Mo1-O21	152.82(5)
O1-Mo1-N17	169.72(6)
O2-Mo1-N27	170.77(6)
C11-O11-Mo1	128.63(13)
C17-N17-C101	115.73(17)
C17-N17-Mo1	118.29(13)
C101-N17-Mo1	123.58(12)
C21-O21-Mo1	134.13(13)
C27-N27-C201	115.01(16)
C27-N27-Mo1	118.93(12)
C201-N27-Mo1	122.51(11)

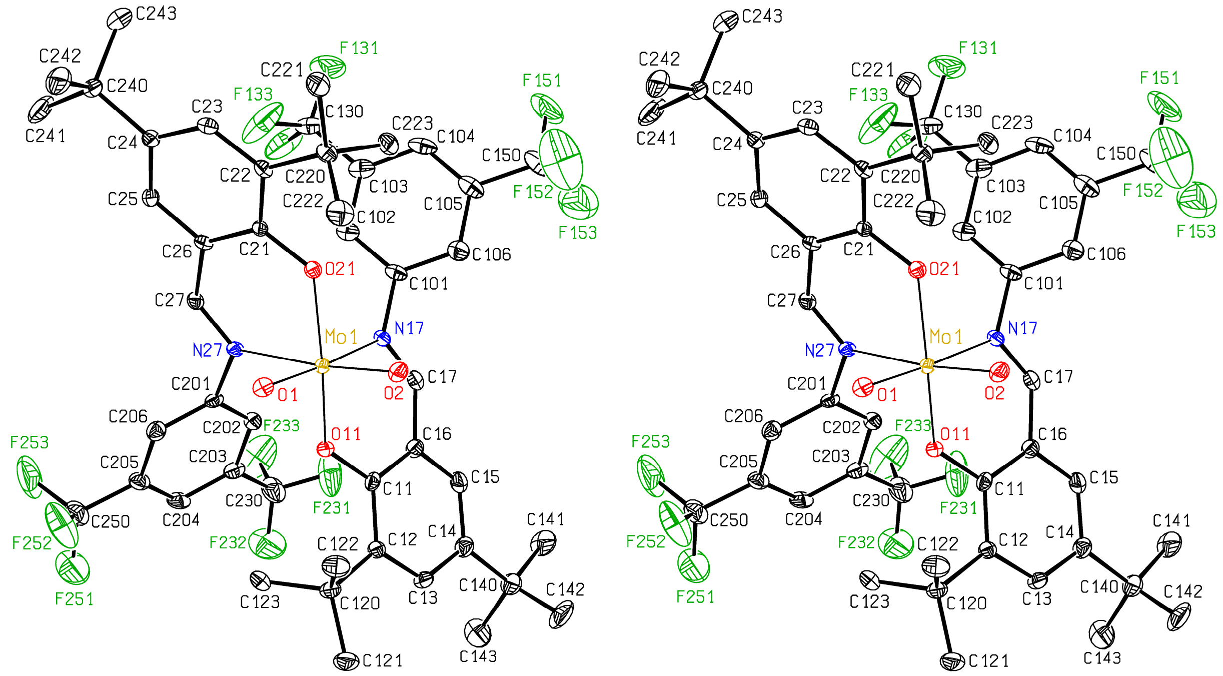

Figure S39. Stereoscopic ORTEP[3] plot of **2** showing the atomic numbering scheme. The probability ellipsoids are drawn at the 50% probability level. The H atoms and the solvent molecules were omitted for clarity.

Table S4. Selected bond lengths [Å] and angles [°] for **2**.

Mo1-O1	1.6989(17)
Mo1-O2	1.7048(17)
Mo1-O11	1.9404(16)
Mo1-O21	1.9412(16)
Mo1-N17	2.395(2)
Mo1-N27	2.358(2)
O11-Mo1-O21	154.66(7)
O1-Mo1-N17	167.87(8)
O2-Mo1-N27	167.74(8)
C11-O11-Mo1	133.83(14)
C17-N17-C101	116.0(2)
C17-N17-Mo1	121.30(17)
C101-N17-Mo1	120.20(15)
C21-O21-Mo1	133.33(14)
C27-N27-C201	115.6(2)
C27-N27-Mo1	121.74(16)
C201-N27-Mo1	120.02(14)

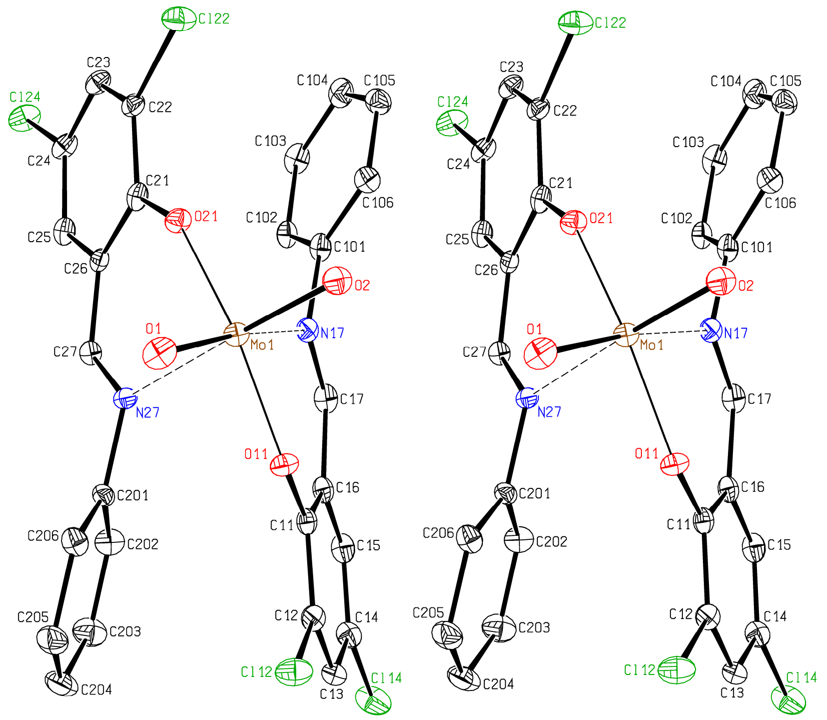

Figure S40. Stereoscopic ORTEP[3] plot of **3** showing the atomic numbering scheme. The probability ellipsoids are drawn at the 50% probability level. The H atoms were omitted for clarity.

Table S5. Selected bond lengths [Å] and angles [°] for **3**.

Mo1-O1	1.705(4)
Mo1-O2	1.712(3)
Mo1-O11	1.950(3)
Mo1-O21	1.946(4)
Mo1-N17	2.407(4)
Mo1-N27	2.382(4)
C17-N17	1.297(6)
C27-N27	1.301(6)
O11-Mo1-O21	154.52(14)
O1-Mo1-N17	167.46(16)
O2-Mo1-N27	165.53(15)
C11-O11-Mo1	139.9(3)
C17-N17-C101	116.0(4)
C17-N17-Mo1	124.1(4)
C101-N17-Mo1	119.7(3)
C21-O21-Mo1	139.0(3)
C27-N27-C201	115.5(4)
C27-N27-Mo1	124.1(3)
C201-N27-Mo1	120.1(3)

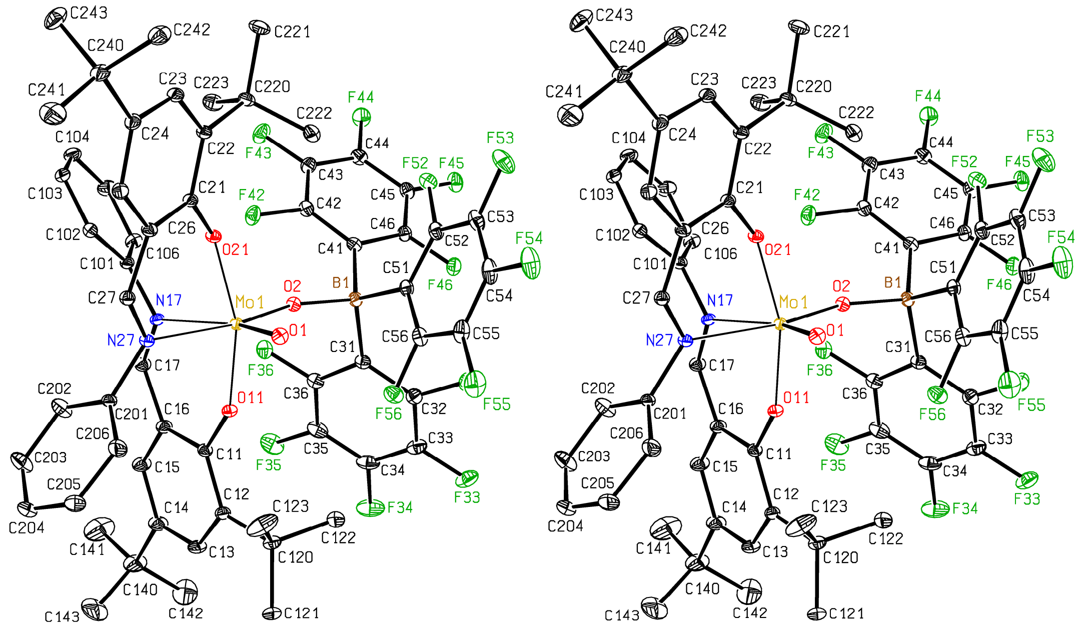

Figure S41. Stereoscopic ORTEP[3] plot of **4** showing the atomic numbering scheme. The probability ellipsoids are drawn at the 50% probability level. The H atoms were omitted for clarity.

Table S6. Selected bond lengths [Å] and angles [°] for **4**.

Mo1-O1	1.6909(11)
Mo1-O2	1.7900(10)
Mo1-O11	1.9008(10)
Mo1-O21	1.9229(10)
Mo1-N17	2.3569(12)
Mo1-N27	2.2967(13)
O2-B1	1.530(2)
B1-C31	1.642(2)
B1-C41	1.638(2)
B1-C51	1.642(2)
O1-Mo1-O2	104.47(5)
O11-Mo1-O21	160.52(5)
O1-Mo1-N17	163.63(5)
O2-Mo1-N27	169.59(4)
N17-Mo1-N27	79.02(4)
B1-O2-Mo1	159.08(9)
C11-O11-Mo1	144.84(9)
C17-N17-C101	113.70(12)
C17-N17-Mo1	123.79(10)
C101-N17-Mo1	122.16(8)
C21-O21-Mo1	135.66(9)
C27-N27-C201	112.81(12)
C27-N27-Mo1	121.60(10)
C201-N27-Mo1	125.14(9)

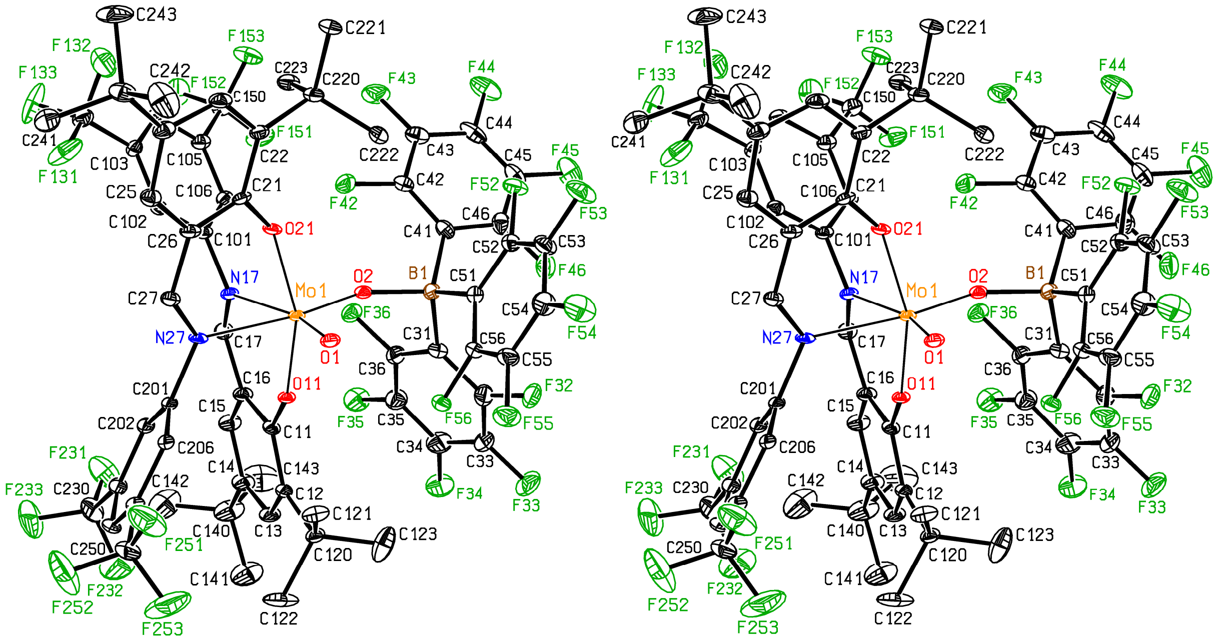

Figure S42. Stereoscopic ORTEP[3] plot of **5** showing the atomic numbering scheme. The probability ellipsoids are drawn at the 30% probability level. The H atoms and the pentane solvent molecules were omitted for clarity.

Table S7. Selected bond lengths [Å] and angles [°] for **5**.

Mo1-O1	1.680(2)
Mo1-O2	1.783(2)
Mo1-O11	1.8937(19)
Mo1-O21	1.922(2)
Mo1-N17	2.381(3)
Mo1-N27	2.312(3)
O2-B1	1.535(4)
O1-Mo1-O2	103.85(10)
O11-Mo1-O21	157.21(9)
O1-Mo1-N17	165.94(10)
O2-Mo1-N27	171.60(10)
N17-Mo1-N27	82.21(9)
B1-O2-Mo1	155.8(2)
C11-O11-Mo1	145.20(19)
C17-N17-C101	114.3(3)
C17-N17-Mo1	123.1(2)
C101-N17-Mo1	121.47(18)
C21-O21-Mo1	136.73(18)
C27-N27-C201	113.1(3)
C27-N27-Mo1	122.66(19)
C201-N27-Mo1	122.25(19)

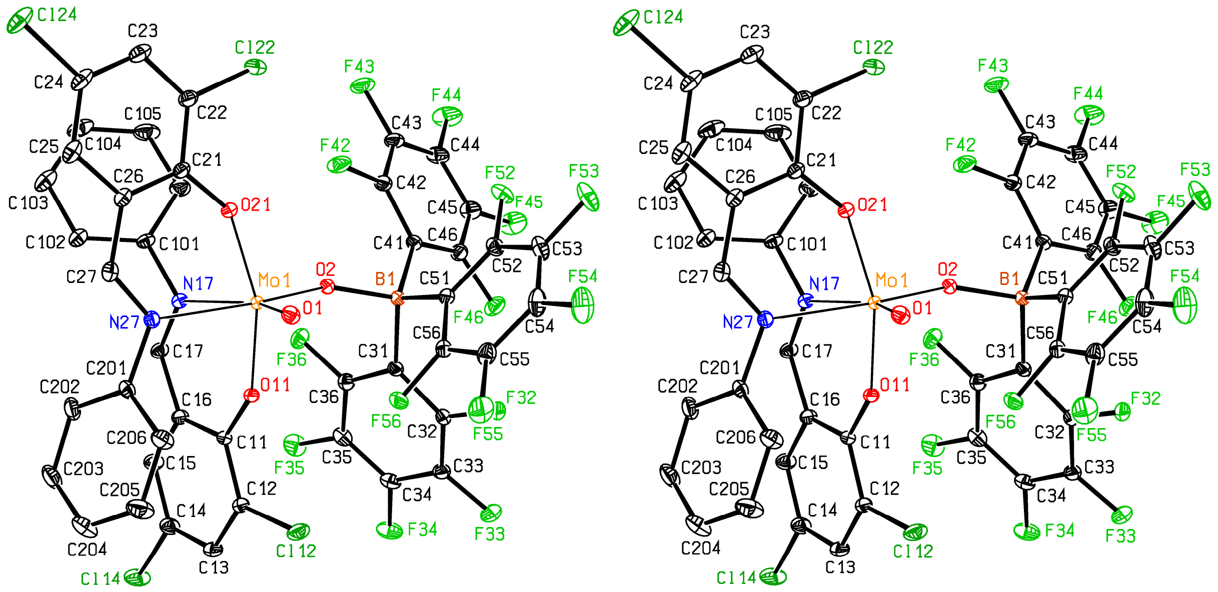

Figure S43. Stereoscopic ORTEP[3] plot of **6** showing the atomic numbering scheme. The probability ellipsoids are drawn at the 50% probability level. The H atoms were omitted for clarity.

Table S8. Selected bond lengths [Å] and angles [°] for **6**.

Mo1-O1	1.6892(9)
Mo1-O2	1.7839(8)
Mo1-O11	1.9121(9)
Mo1-O21	1.9072(9)
Mo1-N17	2.3950(10)
Mo1-N27	2.3084(10)
O2-B1	1.5371(15)
O1-Mo1-O2	104.37(4)
O11-Mo1-O21	157.99(4)
O1-Mo1-N17	159.94(4)
O2-Mo1-N27	169.94(4)
N17-Mo1-N27	153.13(8)
B1-O2-Mo1	144.80(8)
C11-O11-Mo1	114.20(10)
C17-N17-C101	126.10(8)
C17-N17-Mo1	119.09(7)
C101-N17-Mo1	141.20(8)
C21-O21-Mo1	116.44(10)
C27-N27-C201	126.51(8)
C27-N27-Mo1	117.02(7)
C201-N27-Mo1	157.99(4)

REFERENCES

[1] Sheldrick, G. M. A short history of SHELX. *Acta Cryst. A* **2008**, *64*, 112–122.

[2] Sheldrick, G. M. Crystal structure refinement with SHELXL. *Acta Cryst. C* **2015**, *71*, 3–8.

[3] Johnson, C. K. *ORTEP*; Report ORNL-3794; Oak Ridge National Laboratory, Tennessee, USA, 1965.
